# Liberalizing the killing of endangered wolves was associated with more disappearances of collared individuals in Wisconsin, USA

**DOI:** 10.1038/s41598-020-70837-x

**Published:** 2020-08-17

**Authors:** Francisco J. Santiago-Ávila, Richard J. Chappell, Adrian Treves

**Affiliations:** 1 Nelson Institute for Environmental Studies, University of Wisconsin – Madison, Madison, USA; 2 Department of Biostatistics & Medical Informatics, University of Wisconsin – Madison, Madison, USA

**Keywords:** Conservation biology, Environmental impact

## Abstract

Although poaching (illegal killing) is an important cause of death for large carnivores globally, the effect of lethal management policies on poaching is unknown for many populations. Two opposing hypotheses have been proposed: liberalizing killing may decrease poaching incidence (‘tolerance hunting’) or increase it (‘facilitated poaching’). For gray wolves in Wisconsin, USA, we evaluated how five causes of death and disappearances of monitored, adult wolves were influenced by policy changes. We found slight decreases in reported wolf poaching hazard and incidence during six liberalized killing periods, but that was outweighed by larger increases in hazard and incidence of disappearance. Although the observed increase in the hazard of disappearance cannot be definitively shown to have been caused by an increase in cryptic poaching, we discuss two additional independent lines of evidence making this the most likely explanation for changing incidence among n = 513 wolves’ deaths or disappearances during 12 replicated changes in policy. Support for the facilitated poaching hypothesis suggests the increase (11–34%) in disappearances reflects that poachers killed more wolves and concealed more evidence when the government relaxed protections for endangered wolves. We propose a refinement of the hypothesis of ‘facilitated poaching’ that narrows the cognitive and behavioral mechanisms underlying wolf-killing.

## Introduction

Globally, loss of large predators has contributed to simplification of trophic structures, lower biodiversity and degradation of ecosystem functions^1–3^. It is widely acknowledged that humans are responsible for more large carnivore deaths than any other cause^4^, although the scientific debate about the sustainability of this killing remains far from settled for many large carnivore populations^3,5,6^. Many policies for large carnivores focus on limiting or regulating human-caused mortality, and many management decisions rely on estimates of human-caused mortality and on understanding the policy effects on such mortality. Therefore, biased estimates of human-caused mortality patterns can undermine policy goals and evaluations (e.g., recolonization by endangered species, restoring ecosystem processes), and harm carnivore population recovery or stability^5–7^. More broadly, accurate estimates of policy effects on illegal killing (‘poaching’ hereafter) of wild animals can improve enforcement of laws for nature protection and adherence to national and international treaties relating to the protection or the restoration of endangered species, ecosystems, biodiversity, and interdiction of unregulated wildlife trade.

Of all direct killing by humans, poaching is the primary cause of death in many carnivore populations^5,8–11^, slowing population growth^12–14^ or hindering recolonization of historic range^8,10^. Poaching is extremely difficult to detect, measure and prevent. Given its illegal nature, poachers often conceal evidence from management authorities tasked with monitoring marked animals. When authorities find the body of a poached animal they might detect that the individual was poached, but many wild animals die undetected. Measurement uncertainty rises from low but non-zero in the latter case to very high when poachers conceal evidence or when marked animals elude monitoring by those authorities. ‘Cryptic poaching’^10^ refers to this type of unreported, concealed illegal killing. Several studies have estimated it as rivaling or exceeding the subset of reported poaching detected and measured by authorities^9,10,15^. Therefore, in places where poaching is often cryptic, official estimates of mortality process and pattern are systematically biased to under-estimate the risk of poaching, unless analysts adequately account for uncertainty. Such accounting has been facilitated by a variety of statistical techniques developed since 2011^10^.

Traditional methods for estimating mortality hazard (i.e., the instantaneous probability of an event such as poaching occurring), incidence (i.e., probability of an event such as poaching occurring in the presence of other mortality sources), and for partitioning those rate parameters among various human and non-human causes typically require data from marked individuals (e.g., collared animals recaptured dead or alive). Yet these traditional methods assume that the marked individuals that are never recaptured or recovered (disappeared hereafter) had suffered from similar causes of death as those recaptured. That assumption proved highly inaccurate for wolves, because systematic measurement biases caused by disappearances and other forms of uncertainty disproportionately affect poaching rather than other causes of death. Failure to account for these biases has led to under-estimating poaching, misidentifying the major cause of death and failing to intervene effectively against wolf-poaching^9,16^. Liberg et al.^10^, to our knowledge, was the first to correct for this underestimation and quantify cryptic poaching. Their analysis of Scandinavian wolf deaths estimated poaching at half of total mortality (51%), with two-thirds attributed to cryptic poaching. Similarly, a later estimate for Wisconsin wolves put the proportion of cryptic poaching at 50%^5^. Efforts to correct poaching estimates for four endangered wolf populations in the contiguous USA found traditional methods overestimated the relative risk of legal killing by 5–16% and underestimated it for poaching by 17–44%^9^. That study also concluded that poaching (observed and cryptic) was the major cause of death for all studied wolf populations.

Quantifying poaching hazard, incidence, and patterns (including its cryptic variant), and how these might be affected by policy, can improve the design of management interventions and thereby hasten restoration and conservation. However, the scientific literature has just begun evaluating the influence of policies on poaching hazard and incidence. A long-held assumption has been that some predator control (e.g.: special permits for killing or hunting seasons) might increase tolerance for controversial species and thus reduce poaching; a claim first argued in a legal brief by the U.S. government in 2006 (Humane Society of US v. Kempthorne, docket DC 06-1279) and articulated as a scientific hypothesis in^17^, and later developed and renamed ‘tolerance hunting’^18–20^. In Wisconsin, USA a series of studies have taken up the question using mortality data. One early study examined reported poaching variation^21^ to hypothesize that frustration with inconsistent management may lead to increased poaching, and colleagues modeled wolf demographic parameters in relation to policy changes (for others, see^22^). However, these studies provide weak inference due to several shortcomings: reliance on correlative analyses, failure to consider cryptic poaching, plus unresolved concerns about modelling of density-dependence and its potential confounding effects of various changes in monitoring methods entangled with so-called ‘recovery periods’^18,23^.

A parallel and independent analysis of the Wisconsin and Michigan wolf populations found that periods with policy that liberalized wolf-killing were followed by significant decreases in potential population growth rates independent of the number of wolves killed legally^12,14^. The authors inferred increases in poaching during six periods of policy that liberalized wolf-killing had caused several decreases in growth rates and that the resumption of more protective policies caused several increases in growth rates. These authors suggested what we now call the ‘facilitated poaching’ hypothesis, which proposes that would-be poachers respond to the policy changes as a signal to increase their activities, possibly associated with cognitive processes relating to values (e.g., lower value of wolves in the eyes of would-be poachers), social norms (e.g., greater acceptability of poaching, or less enforcement against poaching), or perceived control (e.g., would-be poachers perceive themselves helping authorities to kill wolves). This hypothesis is supported by four quantitative surveys of residents of Wisconsin from 2001 to 2013 and two qualitative focus groups from 2011 to 2012, which revealed increased inclinations to poach after Wisconsin wolf policies liberalized killing^24,25^. Three critiques of the ‘facilitated poaching’ hypothesis were published, and one critique of the ‘frustration’ hypothesis, so the scientific debate is lively but it remains based on indirect evidence and weak to moderate strengths of inference^26^.

Subsequent research linking wolf mortality to population growth rates in Finland found poaching rates increased as a response to increases in wolf population size^8^. Follow-up research by the same team found the total number of legally hunted wolves at the local scale and the country scale decreased the probability of poaching, while increases in the number of permits issued to kill wolves (the ‘bag limit’) increased the probability of poaching^27^. The authors hypothesized that the declines in probability of poaching, given more wolves killed through legal hunting, might reflect a decrease in the number of individual wolves exposed to poaching because they were instead legally killed prior to potential poaching, essentially “just cleaning up the numbers”^27^. However, their analyses did not statistically account for the uncertainties in causes of death and disappearance.

No study explicitly modeled the durations and periods of policy that individual wolves were exposed to legal killing^12,28^. That would allow for the estimation of mortality hazard and incidence (from various causes) for individual wolves that *experienced* the policy over time. With individual-level estimates of hazard and incidence for marked individual wolves, we can more confidently draw inference about population-level effects on the growth rate and patterns of poaching. Nor did these studies model the effect of legal killing on wolf disappearances (those animals ‘lost-to-follow-up’; LTF). LTF animals could not have been killed by legal means or by conspicuous causes, otherwise their carcasses would have been recovered^5,9^. Thus, LTF could conceal a component of cryptic poaching, in addition to those collared individuals that moved out of radio-telemetry range or those who died from natural causes but whose radio-transmitters suffered mechanical failure beforehand.

Here we test the hypothesis that poaching (both observed and cryptic) of adult wolves in Wisconsin, USA was influenced by changes in government policies via effects on individual wolf deaths and disappearances (from 1979 to 2012, 513 collared adults), which we modeled by mortality hazard and incidence in a competing risks framework. Widely used in the biomedical literature for the estimation of risk and prognosis for health interventions, competing risk analyses allowed us to estimate both hazards and incidences of various causes of death or disappearance in relation to wolves’ exposure time to policy. Therefore, competing risk analyses illuminate one fate (‘endpoint’ hereafter) among many, to understand the effects of policy for individual wolves, for all endpoints, especially LTF and its cryptic poaching component. Following recommendations for the most rigorous approach to competing risk analysis^29–32^, we report results on all endpoint-specific hazards and CIFs and synthesize findings from both. In interpreting and discussing the results of our analyses, specifically point estimates and compatibility intervals, we follow the recommendations of researchers who argued for expanding discussion beyond traditional, arbitrary thresholds of ‘statistical significance’^33^. Instead, we provide point estimates and compatibility intervals (i.e., ‘confidence intervals’) for our MAIN imputation scenario. We present and discuss the distributions of parameters of interest as well as simulation scenarios for 26 wolves with incomplete data (see “Materials and methods” section). In our discussion, we focus on the resulting point estimates as the most compatible values given our data and assumptions. We then discuss the implications of our model assumptions and uncertainty in our data, in particular for those results relevant to policy effects on mortality hazards and incidences.

Our results suggest that reduced protections under the Endangered Species Act (ESA) for wolves in the form of policies allowing selective liberalized killing may increase wolf mortality risk and incidence beyond the wolves legally killed. Given the ubiquity of large carnivore poaching, our research and methods can improve the effectiveness of many jurisdictions’ policies on environmental crimes, endangered species, and protections for wild animals.

## Results

The six periods (Supplementary Table S2) during which policy that liberalized wolf-killing were associated with various significant changes in endpoints for collared adult wolves**,** whether one examined hazards from Cox models, subhazards from Fine-Gray (FG) competing risk models, or their cumulative incidence functions (CIFs).

### Policy and covariate effects on endpoint hazards

The 6 policy periods with liberalized killing (Supplementary Table S2) were 10–33% more hazardous for wolves to be lost-to-follow up (LTF) than policy periods with full protection. Liberalized killing periods were also more hazardous for legal killing, not surprisingly. Liberalized killing periods were less hazardous for monitored wolves reported poached than periods of full protection. We compare those three effects directly below, in light of existing theory.

Liberalized killing periods were more hazardous for two causes of death, the nonhuman and uncertain endpoints, and less hazardous for collisions, than periods of full protection. Winters were at least twice as hazardous as summers for the three most common endpoints (LTF, poached and nonhuman).

#### Lost to follow-up (LTF)

**Figure 1:**
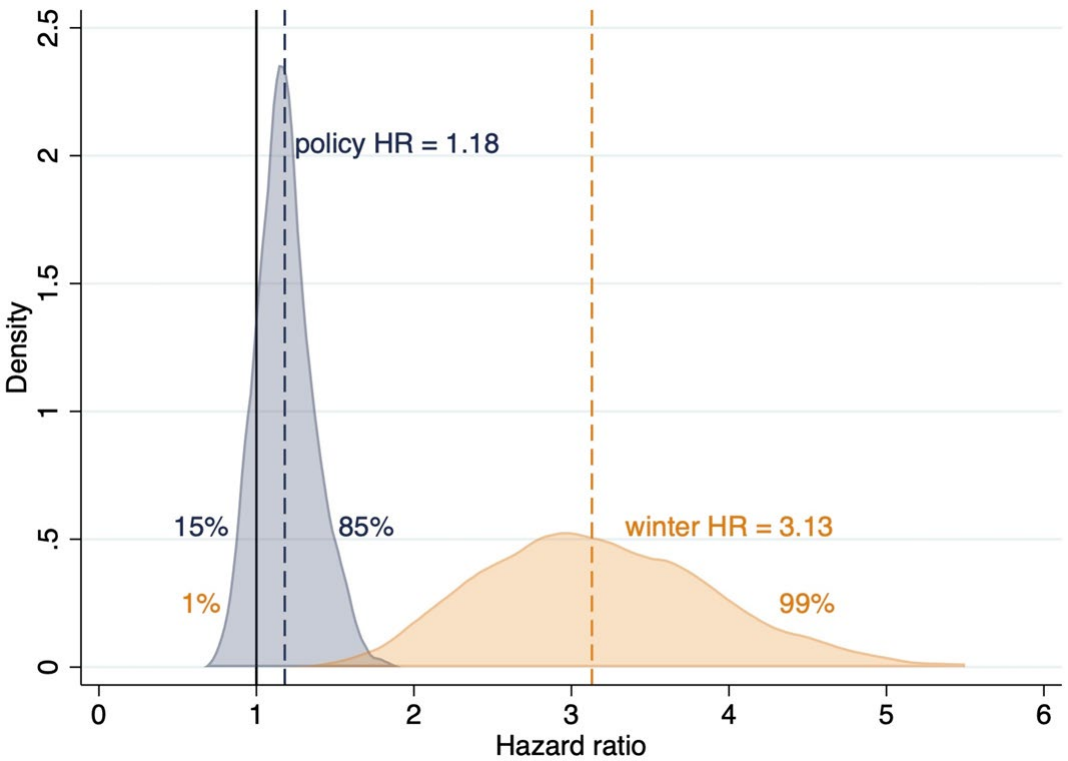
Hazard ratio (HR) of wolves lost to follow-up (LTF, n = 243 in MAIN* scenario) during liberalized killing policy periods (blue) relative to periods of full protection and during winter (orange) relative to summer. Bell curves illustrate the HR distributions with the same color of dashed lines and text as the bell curves to which they correspond for HR point estimates (n = 513). The vertical black solid line at HR = 1 (no effect) is provided for comparison to dashed lines indicating HR point estimates for covariates. Probabilities (%) of a HR of < 1 (left side) or > 1 (right side) are shown with color-coded text for each HR distribution. *We built LTF (or censored) endpoint imputation models (IMs) in three scenarios for 26 collared wolves with missing endpoints (5.1% of collared wolves, see “Materials and methods” section and Supplemental Text). Our MAIN imputation scenario resulted in 12 of the 26 wolves going LTF (average *T* = 947 days), which is consistent with the expected proportion from the aggregate data in which 46% had an LTF endpoint. Results for the LOW and HIGH scenarios are presented in Supplementary Table S7 and narrowed the bounds of the LTF CI.

Liberalized killing periods were 18% (HR 1.18) more hazardous than periods of full protection, yet compatible with a relatively narrow 13% decrease to a 60% increase in hazard (HR 95% CI 0.87–1.60). The resulting LTF HR distribution suggests an 85% probability of an increase in LTF hazard from the liberalized killing policy period (Fig. 1, Table 1). These estimates depend on imputing the LTF (or ‘censored’) endpoint for 26 collared wolves whose records were incomplete in 2012 (Supplementary Tables S3, S4). We report the conservative MAIN imputation scenario in Fig. 1 (see Supplementary Tables S5, S6 for model diagnostics), which is consistent with the distribution of LTF in the overall sample, but also offer two alternative scenarios (LOW and HIGH) that generate HR estimates resulting in narrower bounds, spanning 10–33% increases in hazard for LTF endpoints (Supplementary Table S7).

**Table 1:** Hazard ratio (HR) point estimates from the stratified joint Cox Model 5 (M5) for n = 513 monitored wolves (for MAIN* LTF imputation scenario), by endpoint.

Variable	Endpoint											
	Lost to follow-up (LTF)		Reported poached		Legal		Nonhuman		Collision		Uncertain	
	HR	95% CI	HR	95% CI	HR	95% CI	HR	95% CI	HR	95% CI	HR	95% CI
Liberalized killing periods (lib_kill)	1.18	0.87–1.60	0.81	0.48–1.35	1.57	0.60–4.07	1.09	0.64–1.87	0.43	0.14–1.36	1.36	0.55–3.34
Winter periods (winter)	3.13	1.99–4.93	4.7	2.47–8.91	1	–	2.03	1.08–3.81	0.48	0.20–1.13	1	–
**Census periods (method_change)**												
Census method 1	1	–	1	–	1	–	1	–	1	–	1	–
Census method 2	1	–	0.35	0.16–0.76	1	–	1	–	1	–	1	–
Census method 3			1	–	1	–	1	–	1	–	1	–
**Time-varying coefficient (tvc)**												
Liberalized killing periods (lib_kill tvc) (change per year)	1	–	1	–	2.07	1–4.29	1	–	1	–	1	–
Winter periods (winter tvc) (change per year)	0.69	0.48–0.69	1	–	1	–	1	–	1	–	1	–

We present HRs and compatibility intervals (95% CI) for all covariate-endpoint interactions. Model selection criteria revealed that M5 was the best model (Supplementary Figs. S1, S2, Tables S5, S6 for model diagnostics).

For LTF endpoints, winters were 213% more hazardous than summers, with a broad range 99–393% (Fig. 1, Table 1). The estimated LTF HR of 3.13 at baseline with a time-varying coefficient of 0.69/year means that winter was very hazardous after initial collaring, but then decreased substantially (3.13 × 0.69 = 2.16 and 3.13 × 0.69 × 0.69 = 1.49), to a 116% and 49% increase in hazard (relative to summer) at 1 and 2 years after collaring, respectively.

#### Reported poached

**Figure 2:**
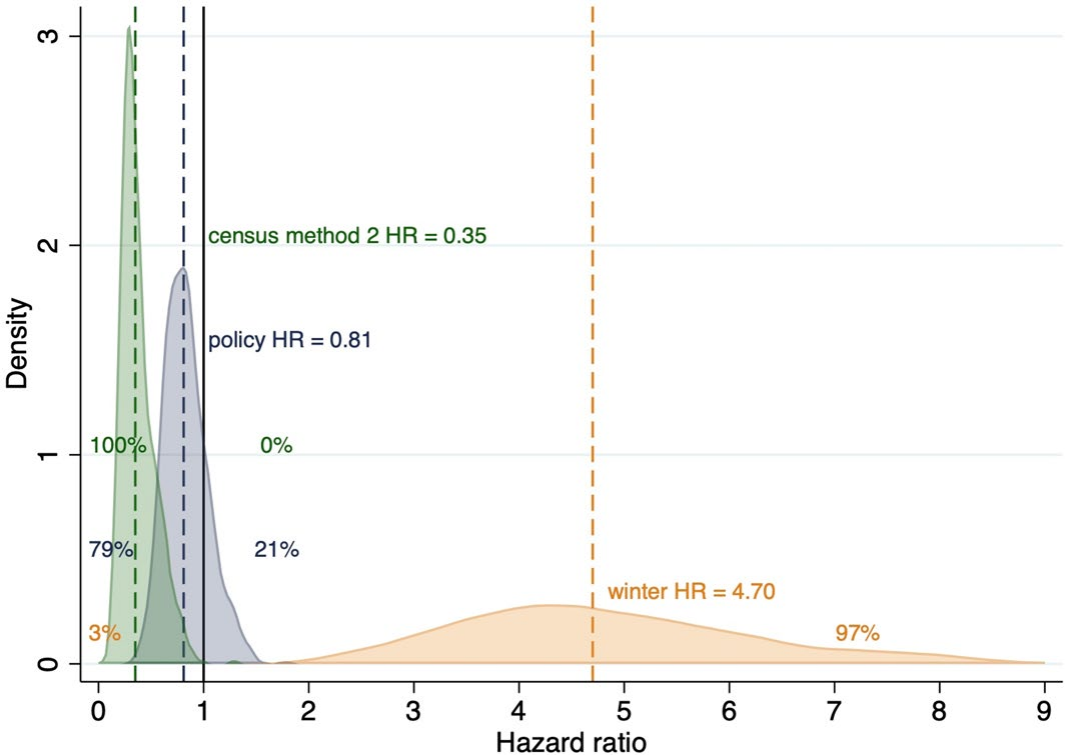
Hazard ratio (HR) of wolves reported poached (n = 88) during liberalized killing policy periods (blue) relative to periods of full protection; winter (orange) relative to summer; census period 2 (1995–2000) relative to census period 1 (1979–1994). Bell curves, vertical lines, text and color coding as in Fig. 1.

Liberalized killing periods were 19% less hazardous for the endpoint of reported poaching (Table 1), yet a 52% decrease to a 35% increase in hazard were also compatible with the data (Table 1, Fig. 2). The resulting poached HR distribution suggests a 79% probability of a decrease in the hazard from the liberalized killing policy signal (Fig. 2). Winters were 370% more hazardous for the endpoint reported poached, with broad compatible estimates spanning increases between 147 and 791% (Table 1, Fig. 2).

We found an effect of census period on reported poaching; a 65% decrease in hazard during census period 2 1995–2000 (relative to census period 1, 1979–1994), with narrow compatible estimates spanning 84–24% decrease in hazard (Table 1, Fig. 1).

#### Legal killing

As the policy intended, liberalized killing periods were 57% more hazardous for the endpoint *legal* than periods of full protection, with compatible estimates spanning a broad range of 40% decrease to a 307% increase. The estimated *legal* HR of 1.57 at baseline with a time-varying coefficient of 2.07/year means that liberalized killing policies were associated with substantial increase over monitoring time (1.57 × 2.07 = 3.25 and 1.57 × 2.07 × 2.07 = 6.73), to a 225% and 573% increase in hazard (relative to full protection periods) at 1 and 2 years after collaring, respectively. The broad range of compatibility estimates indicates variability in the time it took for marked wolves to die from this cause, in policy periods ranging from x to y days of liberalized killing.

#### Nonhuman, collisions and uncertain

Liberalized killing periods were 9% more hazardous for the endpoint of dying by nonhuman cause (57% decrease for collisions; 36% increase for uncertain), with a narrow range of 36% decrease to an 87% increase also compatible with our data (a broad range of − 86% to + 36% for collisions; and a broad range of − 45% to + 234% for uncertain). Winters were associated with an increase in the nonhuman endpoint HR of 103%, with compatible estimates spanning increases of 8–281% (Table 1).

Next, we evaluated how the same covariates as above affected the incidence of each endpoint, using FG models to consider the effect of competing risks on a single endpoint. In FG models of incidence, common endpoints gain importance and rarer endpoints lose importance proportional to their prevalence in the population. By comparison with hazard ratios that do not consider monitored wolves experiencing other endpoints, subhazard ratios consider all endpoints in the dataset up to the time point in question.

### Policy and covariate effects on endpoint incidences in a competing risk framework

FG models revealed liberalized killing periods were associated with an increase in incidence of LTF of 11–34% relative to periods with full protections (Table 2, Supplementary Table S7). As intended by the policy, the former periods were associated with an increase in the incidence of legal killing. By contrast, liberalized killing periods were associated with a 24% decrease in incidence of reported poaching. FG models also suggest an increase in incidence of nonhuman and uncertain endpoints, along with a decrease in the incidence of death by collision.

**Table 2:** Subhazard ratio (SHR) point estimates from FG models for 513 monitored wolves for MAIN imputation scenario, by endpoint.

Variable	Endpoint											
	Lost to follow-up (LTF)		Reported Poached		Legal		Nonhuman		Collision		Uncertain	
	SHR	95% CI	SHR	95% CI	SHR	95% CI	SHR	95% CI	SHR	95% CI	SHR	95% CI
Liberalized killing periods (lib_kill)	1.19	0.86–1.65	0.76	0.44–1.31	1.57	0.59–4.12	1.17	0.67–2.03	0.42	0.13–1.37	1.32	0.55–3.16
Winter periods (winter)	2.89	1.89–4.42	3.36	1.88–6.00	1	–	2.04	1.17–3.54	0.53	0.25–1.14	0.39	0.16–0.96
**Census periods (method_change)**												
Census method 1	1	–	1	–	1	–	1	–	1	–	1	–
Census method 2	1	–	0.37	0.17–0.81	1	–	1	–	1	–	1	–
Census method 3	1	–	1	–	1	–	1	–	1	–	1	–
**Time-varying coefficient (tvc)**												
Liberalized killing periods (lib_kill tvc) (change per year)	1	–	1	–	2.07	1–6.17	1	–	1	–	1	–
Winter periods (winter tvc) (change per year)	0.69	0.48–0.69	1	–	1	–	1	–	1	–	1	–

We present SHRs and compatibility intervals (95% CI) for all covariate-endpoint interactions.

#### LTF

Along with a suggested increase in LTF hazard (Table 1), disappearances of wolves were 19% more likely during policy periods with liberalized killing, relative to periods of full protections (i.e., the proportion of wolves over time going LTF increases), with compatible estimates from the MAIN imputation scenario spanning a 14% decrease to a 65% increase in incidence (Table 2). That range of compatible SHR values was narrowed by our imputation scenarios for the 26 wolves with missing data to a narrower 11%-34% increase in LTF incidence (Supplementary Table S7).

LTF incidence also increased by 189% during winter (relative to summer), with a nroad compatibility interval suggesting increases of 89–342%. The model also detects a non-proportional change (winter tvc) amounting to a 31% decrease in LTF incidence during winter periods for every year of monitoring a given marked wolf (Table 2).

#### Reported poached

Liberalized killing periods were associated with a decrease of 24% in the incidence of reported poaching, with broad compatible estimates spanning a 56% decrease to a 31% increase in incidence (Table 2). Winters were associated with an increase in incidence of 236%, with compatible estimates spanning a broad range of 88–500% (Table 2). During census period 2 (1995–2000), the incidence of reported poaching decreased by 63%, with compatible estimates spanning a narrow range of decreases of 83–29% (Table 2).

#### Legal killing

Consistent with the increase in hazard and the objective of the policy change, liberalized killing periods were associated with a 57% increase in the incidence of legal killing, with compatible estimates spanning a broad range of 41% decrease to a 312% increase. Along with this main policy effect, we obtain a similar (to the HR) non-proportional change amounting to a 107% increase in incidence during those policy periods for every year of monitoring a given wolf (Table 2).

#### Nonhuman, collision and uncertain

Liberalized killing periods were associated with a 17% increase in the incidence of death by nonhuman cause (58% decrease for collisions; 32% increase for uncertain), with broad compatible estimates spanning a 33% decrease to an 103% increase (− 87% to + 37% for collisions; − 45% to + 216% for uncertain). Winters seemed to increase the incidence of a nonhuman endpoint by 104%, with compatible estimates spanning increases of 17–254% (Table 2). The FG model for uncertain suggests an additional (to that of the Cox model) effect of census period associated with a 61% decrease in incidence of this endpoint, with compatible estimates spanning a narrow range of 84–4% decrease in incidence (Table 2).

**Figure 3:**
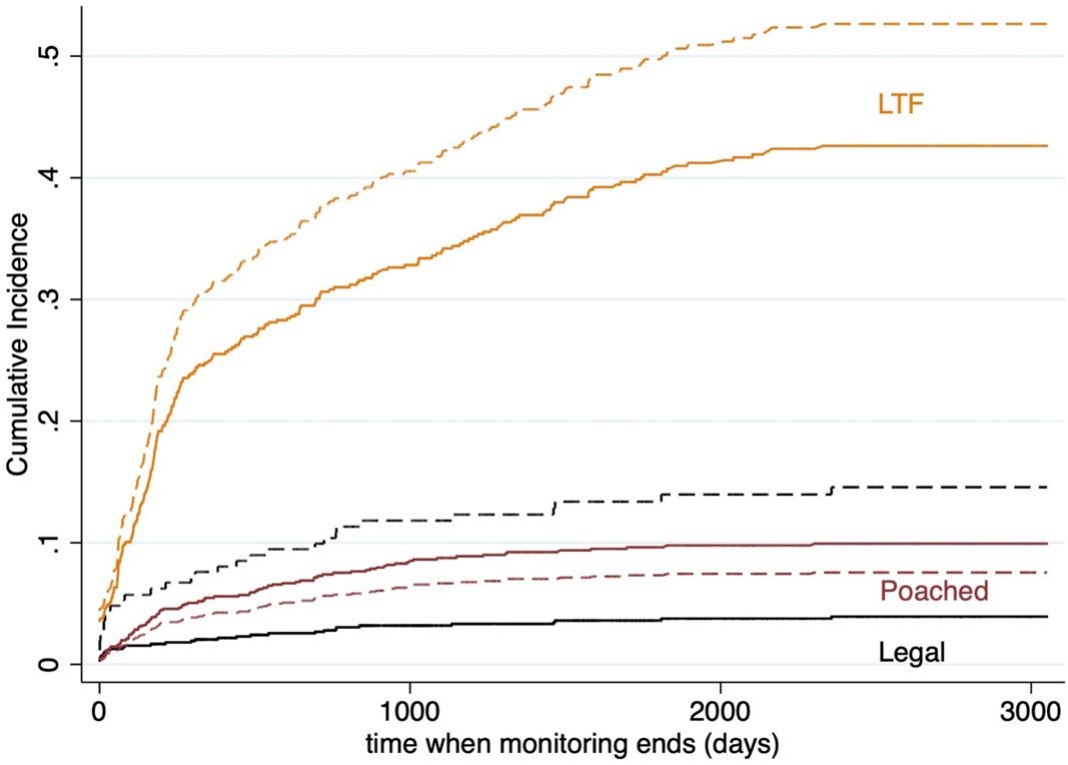
Cumulative incidence functions (CIFs) for 513 monitored wolves. Lines show separate endpoints for lost-to-follow-up, LTF (n = 243, orange), reported poached (n = 88, maroon), and legal kills (n = 32, black) in two periods, derived from Fine-Gray models for MAIN imputation scenario. For each endpoint, we illustrate the cumulative incidence for liberalized killing periods (dashed lines) and periods of full protection (solid lines). We derived CIF curves according to the M5 stratified joint Cox model (Supplementary Figs. S3–S8) and from each endpoint-specific semi-parametric FG models (Supplementary Tables S8, S9 for LTF and legal SHRs used for estimating FG CIFs, and Supplementary Figs. S9–S14) and non-parametric FG models (Supplementary Figs. S15–S20). Visual comparison of the three sets of CIF curves for each policy period-endpoint combination suggests consistent results between Cox and FG CIFs for most endpoints as well as compliance with FG model assumptions (i.e., proportionality of endpoint subhazards) for all endpoints except nonhuman (Supplementary Fig. S5).

We focus on the FG-derived CIFs (Fig. 3) because these consider the prevalence of endpoints in the population at which scale the hypotheses make predictions. The most relevant endpoints (LTF and poached) CIFs suggest liberalized killing periods were associated with an increase in the cumulative incidence of LTF of approximately 0.10 relative to full protection periods (Fig. 3; range 0.05–0.15 for LOW–HIGH imputation scenarios [Supplementary Tables S8, S9 Supplementary Figs. S21, S22]). This increase in LTF incidence is comparable to that of legal killing because of the high prevalence of LTF (i.e., the total number of LTF endpoints in the wolf population). Moreover, the increase in incidence of LTF associated with liberalized killing periods was 5 times larger than the associated decrease in the cumulative incidence of wolves reported poached during the same periods (0.02–0.03 decrease; Fig. 3).

## Discussion

Traditional time-to-event models in wildlife science discard or censor data from marked animals that disappeared (no collar or carcass recovered) as uninformative. This approach fails to account for the high certainty attached to rates of legal causes of death compared to the low certainty about rates of other causes that are not well reported, including the least documented form termed cryptic poaching. Instead, the assumption that individuals that are lost-to-monitoring suffer from similar hazards and endpoints as monitored individuals, or survive through migration or dispersal, produce systematic biases. These biases may underestimate mortality and its anthropogenic component, but more perniciously, these biases may obscure or mislead during the evaluation of any policy effects on mortality hazard and incidence. Our competing risk analyses illuminate how to evaluate policy effects on mortality without introducing the aforementioned assumptions leading to overwhelming biases.

The distribution of the LTF hazard ratio HR suggests a liberalized killing policy signal was associated with an 85% likelihood of increasing the risk of disappearance (Fig. 1) for monitored adults in Wisconsin. The situation is partially reversed for reported poaching, with the liberalized killing policy signal being associated with a 79% probability of a smaller decrease in hazard of being reported poached (Fig. 2). Neither of these changes reached statistical significance (Table 1), yet apparent similarities in these HR distributions are misleading because the incidence of disappearances (LTF) is so much greater than the incidence of observed poaching. We discuss the limitations of our study but when considering all the evidence, we infer that the policy of liberalizing wolf killing in Wisconsin from 2003 onward resulted in more cryptic poaching.

We found that periods with policies that liberalized wolf-killing were most compatible with increases in the hazard (10–33%) and more importantly, in the incidence (11–34%) of disappearances (LTF) among monitored wolves. The same periods were associated with decreases in the hazard (19%) and incidence (24%) of reported poaching of monitored wolves, along with an association with census method never before reported (discussed further below). Given the low number of observations as well as illustrated CIFs (Supplementary Figs. S17–S19), we are unable to discern any policy effects for the collision, uncertain, and nonhuman endpoints.

The suggested decline in reported poaching does not compensate for the LTF increase because LTF had a much higher prevalence in the dataset (n = 243; 47% compared to n = 88; 17.2% for reported poached). The importance of the relative prevalence of LTF and reported poaching is shown in cumulative incidence curves in Fig. 3. Indeed, the increase in cumulative incidence of LTF associated with liberalized killing policies seems comparable only to that of legal killing. Indeed, legal killing crossed HR = 1 (95% CI 0.60–4.07; Table 1), indicating that variance and uncertainty in something we know increased in those periods does not weaken the inference that hazard increased for legal killing. Rather, the compatibility interval indicates the variability in time it took for a monitored wolf to be killed and how many survived such periods. Moreover, we would argue the shape of the HR distributions (Figs. 1, 2) are important; both are sharply peaked and with considerably less uncertainty than our seasonal covariate.

The LTF endpoint is certainly an aggregation of three components: (1) individuals that had moved out of range of aerial radio-telemetry (i.e., long-distance migration), (2) collars that stopped transmitting (i.e., mechanical failures), and (3) unreported poached individuals (‘cryptic poaching’)^5^. Thus, the increase in LTF incidence associated with liberalized killing policies could result from increases in any of these components. We discuss at length each component below but we foreshadow our inference that the major component is cryptic poaching simply because there is no known mechanism through which a policy would cause wolves to migrate out of state or cause mechanical failure of collars. Based on our point estimates and resulting CIFs, our findings are consistent with the hypothesis that ‘culling increases poaching’^12^, compatible with growing evidence of cryptic poaching of predators around the world, and inconsistent with the U.S. federal government’s claim in federal court that liberalizing wolf-killing would reduce poaching and protect the endangered wolves we studied here.

A plausible hypothesis for how liberalized killing periods may increase the incidence of emigration of monitored wolves would be through legal killing possibly causing disruption of wolf behavioral and social dynamics^34–36^, leading to breeding pair dissolution or pack disbanding and perhaps increasing the number of dispersers leaving the state before the next monitoring period. However, the scientific evidence on the effects of anthropogenic mortality on wolf dispersal to date suggests the opposite effect; that low-to-moderate levels of anthropogenic mortality may instead be compensated by increased immigration from adjacent populations, and increased pup survival^15,37^. In their analysis of the effects of anthropogenic mortality on the wolf population in northern Alaska, Adams et al.^15^ conclude that immigration was the main mechanism allowing otherwise unsustainable killing to continue for several years. Consistent with this, 7 times more radio-collared wolves entered Wisconsin from neighboring Michigan than went in the reverse direction, especially during the years with liberalized wolf-killing policies^5^. If liberalizing wolf-killing prompted more emigration by Wisconsin wolves, one might expect more vehicle collisions also. We found the opposite (Table 2). Therefore, emigration out of state seems an unlikely mechanism for the increase in LTF incidence.

Regarding collar failure, we are unable to suggest a possible mechanism associating the incidence of collar failure with liberalized killing periods for several reasons. First, given the technological advances related to collaring and monitoring between 1979 and 2003 (when liberalized killing periods begin; Supplementary Table S2) it seems unlikely that the incidence (or risk) of collar failure would be higher during these later time periods relative to periods of full protections. Moreover, if increased collar failure was a possible mechanism we would expect the incidence (or risk) of collar failures to increase as a function of monitoring time instead of the observed proportional increase in incidence during liberalized killing periods relative to full protection periods. To this we add that policy periods were implemented multiple times during those later time periods (Supplementary Table S2), which makes a confounding effect of collar failure implausible. Regarding the potential for lower temperatures to reduce battery life and affect LTF in winter, looking at both seasonal effects would seem to suggest cold temperatures having two contradictory effects on battery/mechanical failure: (1) a decrease in battery life relative to summer (main winter HR/SHR), but (2) also a decrease in this difference over time (illustrated through the season time-varying effect). Further, the magnitude of the seasonal effect on LTF, an increased risk of 213% in winter relative to summer, seems large enough to implicate a mechanism other than battery life.

The ‘facilitated poaching’ hypothesis proposes a human cognitive mechanism through which liberalized killing policies affect human behavior. The policy signal might decrease the value of wolves for potential poachers or increase the acceptability of poaching by their associates. Social survey research reported rising inclinations to poach wolves in Wisconsin three times after the implementation of liberalized killing policies^24,25,38^, consistent with the ‘facilitated poaching’ hypothesis. In the three studies cited, certainty about the policy effect compared to confounding effects increased as the intervals between resampling respondents decreased with each successive study.

The increases in incidence of monitored wolf disappearance (LTF) during liberalized killing periods suggest how the LTF component of cryptic poaching may obscure (at least part of) the additional mortality necessary to explain the slow-down in the population’s annual growth rate from 2003 to 2011^22^, and is consistent with increases in mortality over and above legal killing during said policy periods that were inferred to be responsible for population growth slow-downs^12,14^. Thus, the ‘facilitated poaching’ hypothesis seems the most plausible explanation for the rise in incidence of disappearances among Wisconsin’s monitored wolves from 2003 to 2012. If this is indeed the case, LTF hazard rose because wolves faced an increased rate of cryptic poaching and incidence rose because the proportion of cryptically poached wolves increased.

The decrease we found in the incidence of reported poaching during liberalized killing policy periods might be interpreted as consistent with the ‘tolerance hunting’ hypothesis, which suggests that some lethal predator control may increase tolerance for the species and thus reduce poaching^17,18^. However, we cannot distinguish changes in reporting from changes in poaching with these data. In any case, looking only at the policy effect on reported poaching dismisses cryptic poaching, especially in light of evidence that most poaching goes unreported and thus underestimated in the Wisconsin wolf population and others^5,9,10^. Given the slight decreases in cumulative incidence of reported poaching that we found (approximately 0.02–0.03) and the more robust increases in LTF incidence (range 0.05–0.15 across our scenarios), it would suffice for just a portion of the suggested increase in LTF incidence to be attributable to cryptic poaching to (over)compensate for any decreases in reported poaching. For example, limiting the observed LTF incidence increase to the proportion of LTF wolves later found by means other than telemetry and found to have been poached (33%; a conservative minimum estimate) would still amount to a 0.02–0.05 incidence increase in cryptic poaching. Thus, our results undermine any claims of reductions in *total* (i.e., observed and cryptic) poaching from liberalized killing policies. (i.e., ‘tolerance hunting’), contra Olson et al.^21,39^.

Additionally, our results for reported poaching also seem consistent with another hypothesized relationship between legal killing and reported poaching from research in Finland. The notion of “cleaning up the numbers”^27^, predicted a decline in reported poaching after increases in liberalized killing simply because fewer wolves are ;eft alive to be exposed to poaching. There seems to be no need to attribute this decline to human cognitive mechanisms (i.e., tolerance); wolves are simply killed legally at a higher rate (higher hazard and incidence) than they are reported poached during these periods (Tables 1, 2, Fig. 3). Indeed, we found that monitoring time (wolf collar transmitting) was associated with an increase in both the hazard and incidence of wolves being killed by government agents during liberalized killing periods. This result should not be ignored by decision-makers because it implies an (unplanned) accelerating incidence of legal killing during prolonged periods of liberalized killing. That is, once government agents are allowed to kill wolves, the likelihood of complaints or wolf deaths increased over time. Our present results seem to implicate human behavior in such a pattern, but further research is needed.

The ‘facilitated poaching’ explanation implies several non-mutually exclusive hypothetical mechanisms by which would-be poachers might respond to a policy signal to increase cryptic poaching. For instance, (a) would-be poachers feel emboldened by a perceived relaxation of anti-poaching laws during liberalized killing periods. This mechanism seems unlikely given cryptic poaching rose 2.5–7.5 times more than reported poaching decreased (Fig. 3). Alternatively (b), would-be poachers perceive a shift to social norms favoring their activities. This too seems unlikely given cryptic poaching remains covert while tolerance for poaching would seem to favor more overt poaching. Finally, (c) would-be poachers interpret wolves as having lower value because the government killing wolves suggests the population is too large. The latter hypothetical mechanism seems viable still and can be measured by classic valuation surveys, even perhaps conducted on the broad public rather than having to find would-be poachers to survey.

Our results also inform the literature on the effect of relaxing protections on environmental crimes. If we are correct in arguing the ‘facilitated poaching’ hypothesis is behind the suggested increases in cryptic poaching, then it seems that legalizing the killing of large nonhuman animals may drive their killing underground and perhaps motivate it, a hypothesis that has found support in research on the elephant ivory trade^40^. The increase in incidence of cryptic poaching we infer without an increase in reported poaching favors the idea that poachers remained averse to the risk posed by the state’s authority to curb poaching (such as with increased enforcement or reestablishment of full federal protections). Here the observed decline in reported poaching incidence in census years 1995–2000 bears mention. Those years were associated with a change in methods to triple or more the number of wolf census-takers each winter^41^. Increasing human presence could have reduced either poaching activity or reporting (although there was no quantification of telemetry effort). The decline in unknown causes of death during the latter census period tends to support a view that additional volunteer census-takers each winter found more wolf carcasses—without any associated change in LTF during that same period. The role of census method requires further study therefore.

Given the scientific evidence suggesting continued declines in tolerance for wolves after the legalization of wolf-hunting in 2012^38^, we hypothesize that cryptic poaching hazard and incidence may have increased after our study period. However, our study, results and scientific inferences are subject to various limitations. Our results are conditioned by any bias inherent in the WDNR data used, such as missing data for 26 wolves that we had to simulate and, in particular, measurement error for date of endpoint. The LTF endpoint is particularly susceptible to measurement error because wolves go LTF between monitoring intervals of weeks or months of unsuccessful monitoring, in most cases without reliable evidence with which to provide estimates of an actual LTF date. Time to event of LTF is the critical parameter in our analyses, not the number of wolves that went LTF, but regardless, the high proportion (119/231 = 52%) of wolves experiencing LTF during the first period of full protection (1979–31 March 2003, Supplementary Table S2) dismisses the concern that the absolute number of collared wolves in later periods potentially confounded our results. Our study is also limited by the lack of individual-level variables (e.g., sex, breeding or dispersal status) that may affect wolf mortality. Moreover, the lack of randomization in the observational study is a weakness. Although we have adjusted for all reasonable and available predictors, the observed effects could still be at least partially attributed to residual confounding variables. However, these results are still interesting and useful for hypothesis generation in the absence of a randomized trial on liberalized wolf killing policy. Until randomized experiments are conducted, ours and other investigators’ inferences are limited by being based on correlative associations, although our analyses enjoyed the benefits to inference that come from longitudinal analyses of long time series covering multiple policy changes.

## Materials and methods

### Data sources

Our dataset includes all collared wolves monitored by telemetry (virtually all VHF radio-transmitters) in Wisconsin (WI) between 1979 and April 2012, published previously in full detail^5^. The dataset includes 486 wolves fitted with collars by the Wisconsin Department of Natural Resources (WDNR) or its agents, plus 27 collared wolves initially captured in the neighboring state of Michigan, which later migrated to Wisconsin (for a total n = 513 individuals).

Our dataset includes 257 wolves that were reported by the WDNR as ‘lost-to-follow-up’ (LTF) because they were not detected via repeated aerial telemetry. LTF may occur for various reasons: (a) individuals that have moved permanently out of telemetry range (i.e., migrants), (b) collars that stopped transmitting because of battery depletion or mechanical failure, and (c) unreported poaching followed by destruction of the transmitter (cryptic poaching). The WDNR suspended telemetry monitoring and assigned an LTF to a wolf if their personnel were unable to detect the collar signal after several months of statewide aerial or ground telemetry. However, the WDNR did not quantify telemetry effort. Dead wolves (n = 242) were recovered by the mortality signals emitted from their collars, after legal killing by management agents, or after private citizens reported a dead wolf between monitoring flights^41,42^. Some LTF wolves were subsequently recovered by means other than telemetry, such as reporting by private citizens. For these cases we used the estimated date of LTF for the endpoint (i.e., death from various causes or disappearance). For fuller treatment of disappearances, detection, and causes of individual wolf death see^5^.

### Estimating conditional hazards

Our analyses exploit the survival history of monitored wolves, measured in days from date of collaring until date of endpoint (i.e., date of death, last monitoring date, or end of our analysis period on April 15, 2012) for each monitored individual.

We modeled endpoint-specific hazard and subhazard in a competing risk framework, which are extensions of survival (or ‘time-to-event’) analyses. Survival analyses estimate ‘time-to-event’ functions, which describe the probability of observing a time interval (T) to an endpoint (‘event’) within a specified analysis time (*t*) that a subject was observed, such that $S\left(t\right)=P(T>t)$ . Alternatively, these techniques allow for calculating the hazard function, ${h}_{k}(t)$, or the instantaneous rate of occurrence of a particular endpoint *k* conditional on not experiencing any endpoint until time *t*^30,43,44^. We also used the (conditional) hazard functions for all endpoints to estimate the probability of any endpoint up to a particular time *T*, i.e., the incidence over time for particular endpoints, such as LTF or death by vehicle collision, nonhuman cause, etc.

Semi-parametric, Cox proportional hazard models estimate how the endpoint-specific ${h}_{k}(t)$ changes as a function of survival time and a set of hypothetical covariates; $S\left(t\right)={e}^{-{h}_{k}(t,x,\beta )}$, where *x* is a vector of covariates acting on the hazard, and *β* is a vector of their respective parameter estimates. The estimation of covariate effects on the endpoint-specific hazard is modeled as ${h}_{k}\left(t\right)= {h}_{0k}(t){e}^{({\beta }_{1}{x}_{1}+\dots +{\beta }_{j}{x}_{j})}$, where ${h}_{0k}(t)$ is an unestimated baseline hazard function (i.e., semi-parametric) and ${\beta }_{j}$ represent estimates of hazard ratios (HRs) for each covariate ${x}_{j}$ (HR < 1 represents a reduction in hazard and HR > 1 an increase in hazard).

The estimated HRs, ${\beta }_{j}$, are assumed proportional throughout the analysis time, *t*, (only differ multiplicatively between categorical covariate levels). Furthermore, we include time-varying effects on hazards and incidences by including interactions between covariates and monitoring time (in days) (see “Model covariates” section)^43–45^. These models allow us to estimate covariate effects on the rate of occurrence of an endpoint looking only at those wolves reaching that endpoint (so that the presence of other endpoints would not affect these estimates). Inference from hazards is limited in the presence of other endpoints competing to bring about the end of monitoring because interaction between endpoint hazards is unaccounted for. Interactions between endpoints are crucial for our tests of hypotheses that relate legal killing to poaching (i.e., illegal killing, both reported poaching and cryptic poaching through the LTF endpoint) at an individual level.

### Estimating unconditional incidences

Competing risk analyses extend standard survival analysis by considering multiple endpoints simultaneously (e.g.: multiple causes of death or disappearance). These models are useful for estimating the incidence of a particular endpoint while accounting for the potential occurrence of all other competing endpoints (e.g., the incidence of wolf-poaching in the presence of other causes of death or LTF). In a competing risk framework, individuals can potentially experience one of multiple mutually exclusive endpoints at each interval *T*. Because only one endpoint can occur first, we refer to the endpoints as ‘competing’ over time, and to the respective probabilities over time as ‘competing risks’.

Rather than estimating the endpoint-specific HRs, as in the Cox model explained above, competing risk analyses estimate the cumulative incidence function (CIF) for each endpoint, defined by the failure probability $Prob(T\le t,D=k)$; the cumulative probability of endpoint *k* occurring over time in the presence of other competing endpoints^30,31,46^. Competing risk analysis accounts for the CIF of any endpoint being a function of all endpoint-specific hazards, ${h}_{k}(t)$, reflecting the rate of occurrence of that endpoint as well as how it is influenced by others^32^.

Although CIFs can be derived by using all endpoint-specific HRs derived from Cox models, such a procedure cannot estimate the magnitude of the relative difference between covariate CIFs for each endpoint. Using Fine-Gray (FG) models instead of Cox models allows us to estimate differences in CIFs for a given endpoint conditional on covariates^31,47^. FG models are also semi-parametric (i.e., the baseline subhazard function is not estimated) and assume proportionality of subhazard functions, defined as the risk of failure at time *t* from endpoint *k* in subjects that have yet to reach an endpoint or have experienced any other endpoint^30,31,47^. Therefore, FG models estimate the subhazard functions of endpoint-specific CIFs using similar regression techniques as the Cox model (but on the subhazard rather than the hazard thus yielding SHR rather than HR for ratios that compare to a standard), but parameter interpretation changes. Subhazards are interpreted as relative incidence in the presence of other endpoints^29–31^.

In sum, endpoint-specific Cox models and their HRs allow us to test the hypothesis that liberalized wolf-killing affected the rate of occurrence of any endpoint; for example, if liberalized killing increased or decreased the rate of occurrence of reported poaching or LTF. By contrast, the FG models and their SHRs allow us to account for the simultaneous presence of all competing endpoints to test if and how much liberalized killing affected the probability and incidence of reported poaching or LTF, in addition to the potential simultaneous effects of other covariates described after data preparation. CIFs allow us to visualize those effects on incidence while considering the prevalence of each endpoint in the population.

### Data preparation

For wolves monitored until death, our endpoints classify the cause of death by 5 mutually exclusive causes of death similar to^5^: “collision” (trauma caused by vehicles; n = 24, 4.7%), “legal” (lethal control by management agencies; n = 32, 6.2%), “poached” (illegal human-caused killing; n = 88, 17.2%), “nonhuman” (causes unrelated to people, e.g.: other wolves or diseases; n = 77, 15.0%) and “uncertain” (uncertain cause but the wolf carcass was recovered, i.e.: difficult to discern in necropsy; n = 21, 4.1%). We added a sixth distinct category of LTF endpoint (n = 231, 45.0%, and see Supplementary Data S1) and we address 40 collared wolves missing endpoint dates (7.7%) below.

We defined the date of endpoint either as the recorded date of death for wolves monitored by telemetry until death (n = 242, 47.2% of sample) or as the date of last telemetry contact for LTF wolves (n = 231, 45.0%). Some of the LTF wolves were found dead later (n = 51), through means other than telemetry (e.g., visual detection), which might bias to a later date of ‘death’, if carcasses were found long after the actual date of death which was not uncommon^5^. Given the sensitivity of time-to-event models to the accuracy of endpoint dates and because most (n = 206, 78% of the LTF subsample) were never detected again, our step to restrict the record histories of LTF wolves to the last date of monitoring is an important yet imperfect improvement in measurement precision.

Accounting for all individuals at risk of experiencing an endpoint at any particular time *T* (the ‘risk set’) is essential for obtaining unbiased estimates of HR, SHR, and CIF^43,44,48^. Omitting a class of individuals (e.g., LTF) strongly biased risk estimates for four populations of wolves, and in the Wisconsin wolf population specifically, as summarized above^5,9^.

### Model covariates

We included three time-dependent categorical covariates in our models. Time-dependent covariates are variables that change value due to external events at a known date, either for individual wolves or all wolves. For example, we modeled policy period as time-dependent by changing the covariate value at the dates of policy change for a particular individual’s history of monitoring. To assign categorical values of the time-dependent covariates to each monitored wolf, we split each history at each specified date of change in covariate value. We refer to the splits for a monitored wolf as ‘spells’, because they refer to briefer time periods within an individual’s total monitoring time *T*. So, the time-dependent categorical covariates have a duration that overlaps the monitoring period for collared wolves during that period, but the wolves have individual spells that might be less than or equal to the duration (see example in Supplementary Table S1).

Our main covariate of interest is policy that liberalized wolf-killing (*lib_kill* where 1 = liberalized killing, 0 = full protection). Gray wolves experienced full protection under the ESA from 1979 to March 31, 2003. From April 1, 2003, wolves in WI and MI were subject to 11 alternating sequential, non-overlapping periods in which wolf-killing policies were first liberalized and then restricted for varied durations (Supplementary Table S2)^5,12,28^. Although WDNR or its agents occasionally killed a wolf during full protection periods, in capture-related accidents or after verified threats to human safety, these were rare and few. By contrast, liberalized killing periods were characterized by an announcement of policy change that allowed managers or private landowners to kill wolves for perceived or verified losses of domestic animals. Liberalized killing periods included:

- ‘Downlisting’ to threatened status (one period starting April 1, 2003; 670 days, Supplementary Table S2)—allows for lethal control in defense of human property or safety as well as for population management or conservation purposes under ESA section Rule 4(d).
- Issuing of sub-permits for “take” (“to harass, harm, pursue, hunt, shoot, wound, kill, trap, capture, or collect, or to attempt to engage in any such conduct” [ESA]) of wolves by managers and sometimes private landowners (periods within 2005 and 2006; 263 days, Supplementary Table S2) under ESA sections 9 and 10.
- ‘Delisting’, or removing ESA protections entirely (periods of 2007, 2009 and 2012; 701 days, Supplementary Table S2).

Choosing to end our study on April 14, 2012 presented several advantages. First, the WDNR summarized wolf census data and population reports for the preceding year on April 15th. Second, we could compare our results to prior work^12,21,49^. Third, the April 2012 passage of Act 169 enacting the first wolf-hunting seasons since wolf bounties were terminated in the 1950s^50^ was a qualitatively different policy signal than those of the liberalized killing periods (Supplementary Table S2).

Our second binary covariate, *winter,* produced spells for October–March (‘1’, winter) and April-September (‘0’, summer). Our inclusion of this variable is warranted by robust independent evidence of seasonal differences in both overall and endpoint-specific mortality^21,51,52^. Most LTF endpoints occurred during winter months (143/231 = 62% of LTF wolves, with n = 40 wolves censored).

Our third covariate had three levels for periods with different methods of censusing wolves (*method_change*). In the winter of 1994–1995 the wolf census methods changed, and did so again sometime between summer 2000 and winter 2003–2004, with changes in monitoring techniques and protocols for data handling^18,23^. Those changes affected effort and training of wolf census-takers, so might have affected the detection and monitoring effort for collared wolves also. Although there is some ambiguity in the literature over the exact dates of these changes, we opted for the following splits based on year of endpoint: 1979–1994 (‘1’), 1995–2000 (‘2’) and 2001–2012 (‘3’).

### Imputation for 2012 records without endpoint data

We right-censored the interval for individuals that did not experience an endpoint during the analysis period (start of monitoring until April 14, 2012), meaning they are considered as part of the risk set from collaring until the end of the analysis period. Our dataset includes 40 wolves without attributed mortality of disappearance data, because we could not find their endpoint (i.e., cause of death or disappearance) in public records after December 31st, 2011 (see supplementary data files for WDNR monitoring records for 2012 and 2013). Although 14 of those 40 wolves were later found dead in mortality reports between May 2012 and October 2013 (Supplementary Data S2), those reports did not reveal the last date of monitoring but rather a lengthy interval without a record of monitoring followed by discovery of the dead animal. Therefore, we conservatively censored those 14 wolves at April 14, 2012 to consider them as within the risk set (monitored) for the corresponding time intervals, yet without experiencing an endpoint during that time. For the other 26 censored wolves that vanished from public records after December 31st, 2011, our repeated efforts to obtain data were not fulfilled by the WDNR. We submitted four separate requests to the WDNR (1 open records request, 1 state Natural Heritage Inventory request, a personal request to research staff who have published analyses with those data, and we enlisted the aid of the lieutenant governor and governor’s offices to request those data) for all collared wolves monitored in the state in 2012. Therefore, we simulated their endpoints in three scenarios described below.

We imputed either an LTF or censored status to the *n* = 26 wolves with missing endpoints based on the rationale that if any of these monitored wolves had suffered a death rather than a disappearance, their deaths should have appeared in mortality records spanning January 1, 2012 (when missing records for these wolves begin) to October 31, 2013, as happened with the 14 wolves with missing endpoint but found in subsequent mortality reports and therefore censored. Thus, the two remaining possibilities are that these wolves were either LTF or survived our analysis period and beyond October 31, 2013 which means they must be included in the risk set but be censored for endpoint analyses because they do not fit our 6 categories of endpoint.

For our simulation scenarios, we developed a series of FG imputation models (IMs) with LTF as the endpoint of interest using the above covariates for the full, original dataset (i.e., with all 40 wolves with missing data classified as ‘censored’ on April 14, 2012). We then used the most appropriate FG model (accounting for Akaike’s Information Criterion (AIC), Bayesian Information Criterion (BIC), log-likelihood (LL), parsimony and proportionality assumptions) to predict the probability of LTF incidence by April 14, 2012 for each of the 26 wolves. Because we assumed all 26 wolves were alive on April 14, 2012 (i.e., each is imputed their maximum *survival* time) for all models, whereas they might actually have disappeared earlier in 2012, our approach is conservative because it likely underestimates the relative incidence of LTF.

To calculate each of the 26 wolves’ probability of LTF, we first calculated the baseline CIF for the best IM and multiplied it by the exponentiated *lib_kill* and *winter* coefficients in Model 2 to obtain a probability of LTF for each wolf during winter periods with liberalized killing, as wolves experienced during the period beginning January 28, 2012 until April 14, 2012 (Supplementary Table S2). Then we ran 1,000 simulations for each wolf going LTF, using a Bernoulli distribution with the LTF probability for each wolf as the probability of success (‘LTF’). For our MAIN imputation scenario, each wolf was imputed an LTF endpoint (on April 14, 2012) if the simulated occurrence of the LTF endpoint was higher than the probability of LTF predicted from the FG model (used as an imputation threshold), ${p}_{i,SIM}\left(ltf\right)>{p}_{i,FG}(ltf)$, otherwise we censored that wolf. To analyze sensitivity to the MAIN scenario, we also developed HIGH and LOW scenarios following a similar imputation process (Supplementary Data S3). For the HIGH imputation scenario, we increased the threshold probability for going LTF by half the difference between ${p}_{i,FG}(ltf)$ and 1; ${p}_{i,HI}\left(ltf\right)={p}_{i,FG}\left(ltf\right)+(1-{p}_{i,FG}\left(ltf\right)/2$. For the LOW imputation scenario, we decreased the threshold probability for going LTF by half of ${p}_{i,FG}(ltf)$; ${p}_{i,LO}\left(ltf\right)={p}_{i,FG}\left(ltf\right)-{p}_{i,FG}(ltf)/2$. The LOW and HIGH scenarios provided bounds on the point estimates of relative hazard and incidence for the simulated LTF process in the MAIN scenario.

### Statistical tests

To model all endpoint-specific HRs, we employed Lunn & McNeil’s (1995 Method B) data augmentation method. Namely, we augmented the data by our 6 endpoint categories and employed stratified joint Cox multiple regression (on endpoint) with interactions between covariates and each endpoint. Our initial model included all interactions. We then discarded the weakest first to follow model selection procedures while retaining the policy variable in all models (7 models total, Supplementary Table S5). The approach provides us with covariate HRs for all endpoints and we use those HRs for estimating the CIFs by policy period for each endpoint. We model HR distributions of covariates for our poaching and LTF by exponentiating a normal distribution parameterized with the covariate coefficients and standard deviations obtained from their respective Cox models.

We also ran separate FG univariate and multivariate models, which mirrored the best stratified joint Cox model, to estimate FG CIFs for each endpoint. We compared CIFs visually to identify the most appropriate CIF model estimate (Cox or FG), following^53^.

Given the complete survival history of each individual wolf was split into multiple spells, we clustered all our regression analyses using a unique identifier for each wolf, following methods in^54^. Clustering on wolf identity accounts for auto-correlation (e.g., all spells are analyzed within-subjects) and avoids pseudo-replication of observations. We evaluated compliance with the proportionality assumptions for each model through the inclusion of time-varying coefficients (tvc). A tvc is an interaction of each parameter with analysis time which models changes in that parameter’s effect over time; i.e., non-proportionality. Endpoint-specific models with significant non-proportionality in a covariate (tvc) cannot provide predictions of risk or incidence due to computational limitations. We further verified proportionality using Schoenfeld residuals^43,44,48^, which should show a random pattern against time as evidence of compliance with the PH assumption. We selected the best regression models considering AIC, BIC, LL, parsimony, and compliance with model assumptions. When we set aside a best model because of non-proportionality, we present and discuss the best model but our CIF calculations use parameters from the same Cox or FG model without the tvc. We visually assessed goodness-of-fit for each selected endpoint-specific Cox model by Cox-Snell residual plots, which should show the Nelson-Aalen cumulative hazard closely following the line of Cox-Snell residuals if the model is a good fit. We conducted all statistical analyses in Stata 15 (StatCorp, College Station, TX, 2015; see supplementary materials for statistical code).

## Supplementary information

Supplementary Dataset 1.

Supplementary Dataset 2.

Supplementary Dataset 3.

Supplementary Information

## Data Availability

All data and statistical code is available in the main text, supplementary materials or from [INSTITUTIONAL DATA REPOSITORY].
