# Supplementary material for: Liberalizing the killing of endangered wolves was associated with more disappearances of collared individuals in Wisconsin, USA
Source: Sci Rep. 2020 Aug 17;10:13881. doi: 10.1038/s41598-020-70837-x (PMC7431570; doi:10.1038/s41598-020-70837-x)
Supplement: Supplementary file 4 — Supplementary Information [file 41598_2020_70837_MOESM4_ESM.docx]

Liberalizing the killing of endangered wolves was associated with more disappearances of collared individuals in Wisconsin, USA

Francisco J. Santiago-Ávila^1*^, Richard J. Chappell^2^, Adrian Treves^1^

^1^ Nelson Institute for Environmental Studies, University of Wisconsin - Madison

^2^ Department of Biostatistics & Medical Informatics, University of Wisconsin – Madison

*corresponding author: [santiagoavil@wisc.edu](mailto:santiagoavil@wisc.edu)

Supplementary Text: Models and scenarios of disappearances of collared wolves

The initial step in our analysis consisted of constructing Fine-Gray (FG) models for imputing either an LTF or censored endpoint to the *n* = 26 wolves with missing endpoint (see Materials and Methods). We constructed five FG imputation models (IMs) of the lost-to-follow up (LTF) subhazard. Most IMs (4 out of 5) suggested increases in relative incidence of LTF during *lib_kill* periods. IM4 and IM5 were the best performing, although IM5 was more parsimonious (Supplementary Table S3). IM5 shows a slight decrease (1.2%) in the relative incidence, or subhazard ratio (SHR), of LTF during liberalized killing periods (*lib_kill*=1) and a large increase (211%) in the relative incidence of LTF during *winter* (=1), along with a time-varying coefficient (tvc) accounting for a non-proportional decrease of the LTF subhazard during *winter* periods. IM4 shows comparable results for the *winter* covariate and tvc, yet with a considerable relative increase in LTF incidence of 23.3% during liberalized killing periods (*lib_kill*=1) periods and a minimal non-proportional relative decrease in incidence during the same periods. IMs 5 and 4 seemed to perform similarly when considering all model statistics, yet their SHR for *lib_kill* were considerably different. Given this and computational limitations of modelling non-proportionality, we used IM2 (a version of either IM4 or IM5 without the tvc variables) to estimate the probability of LTF for individual wolves (Supplementary Table S4). IM2’s *lib_kill* SHR (1.023) provides us with a conservative estimate of the policy effect (the SHR estimate is much closer to IM5 than IM4), while still accounting for the *winter* covariate, solely for the purpose of simulating the 26 collared wolves missing endpoints.

Using IM2, LTF probabilities in our simulations of endpoints for 26 missing wolves, our MAIN imputation scenario resulted in 12 of the 26 wolves going LTF (average *T* = 947 days), which is consistent with the expected proportion from the aggregate data in which 46% had an LTF endpoint. The HIGH scenario resulted in 20 LTF wolves (20/26 = 77%; average *T* = 640 days) and the LOW scenario resulted in 7 LTF wolves (7/26 = 27%; average *T* = 723 days; Data S3). DNR Endangered Resources report #143, the most precise and complete report made public, reports 20 wolf disappearances yet still lacks evidence for date of LTF and still lacks wolves' endpoints, and therefore our LOW scenario (20 wolves LTF between April 15, 2011 – April 14, 2012) is realistic if no other wolf went LTF but our MAIN scenario seems most likely and our HIG scenario is plausible too (25 and 32 LTF wolves between April 15, 2011 – April 14, 2012, respectively).

Supplementary Figs. S1 to S22


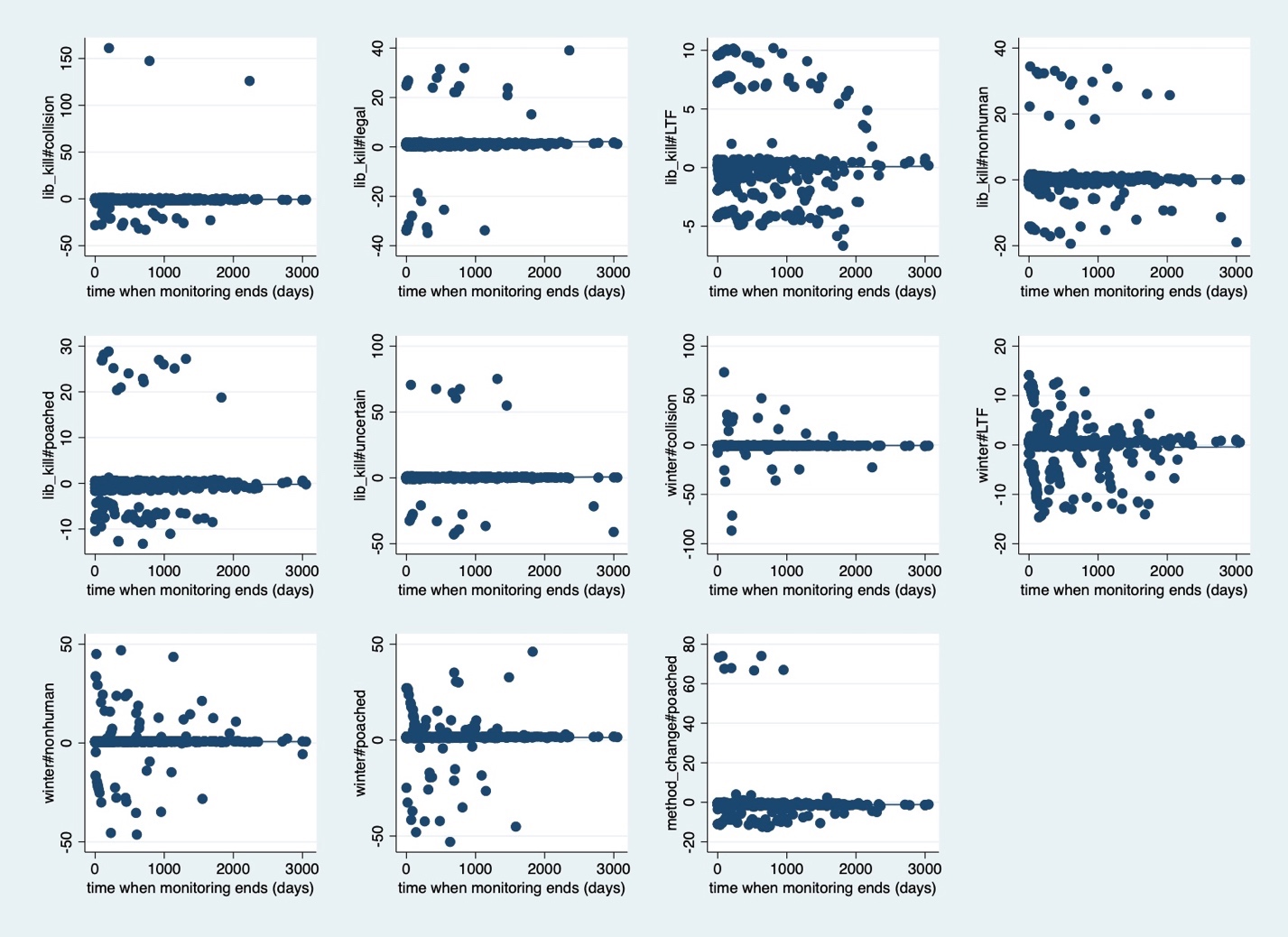


**Supplementary Fig. S1**. Schoenfeld residual scatterplots for each covariate-endpoint (*covariate#endpoint*) combination in the best (M5) stratified joint Cox model.


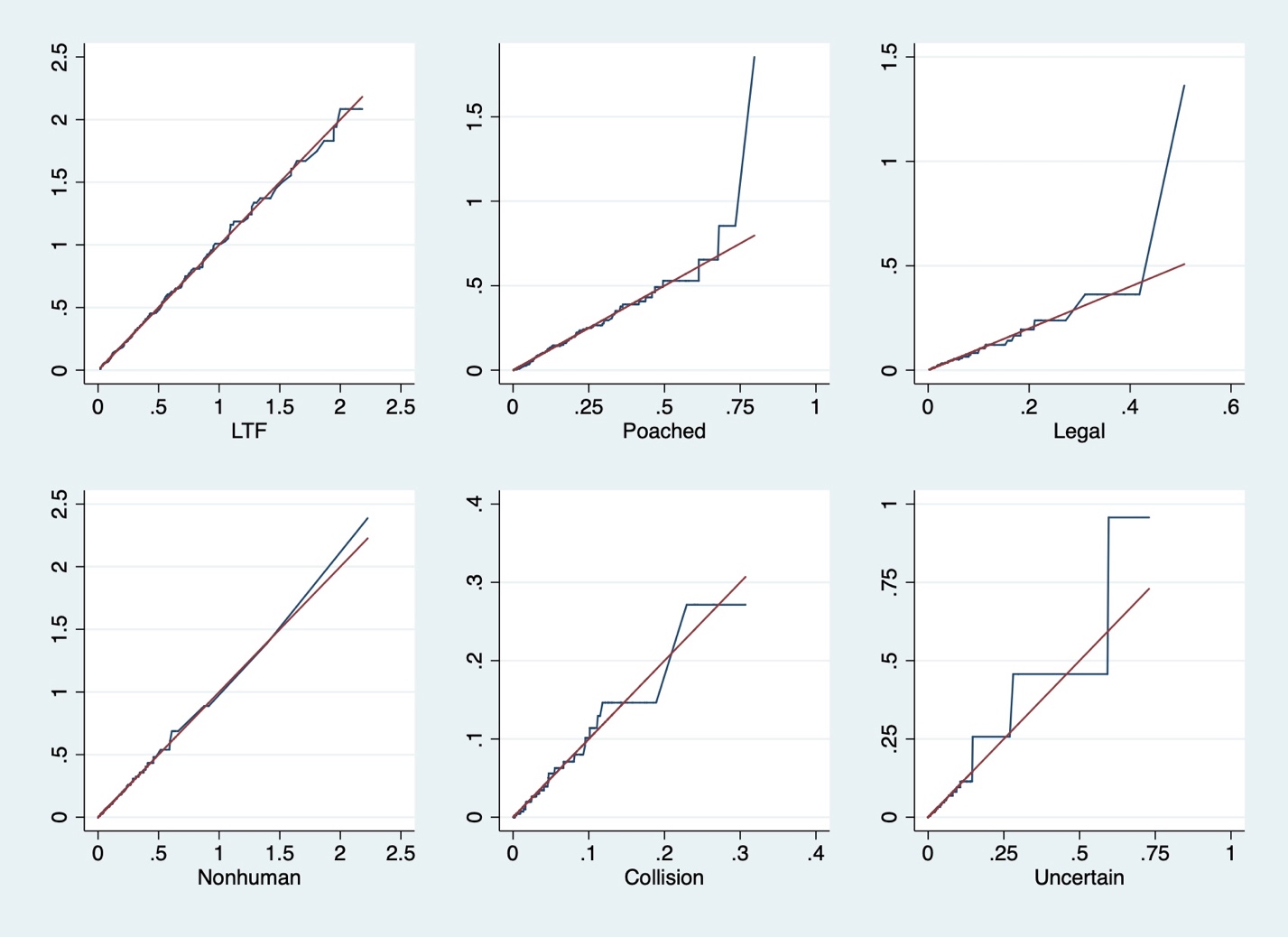


Supplementary Fig. S2. Goodness of fit, by endpoint, for MAIN simulation scenario. Cox-Snell generalized residuals (red lines, on x axis) used for evaluating stratified joint Cox model M5. The Nelson-Aalen cumulative hazard (blue lines, on y axis) follows closely the 45˚ (red) line of Cox-Snell residuals for the most prevalent endpoints (LTF, poached, nonhuman), suggesting good model fit. Lines of less prevalent endpoints (uncertain, collision, legal) show overall consistency with Cox-Snell residuals although with some divergence at large values (fairly common when censoring data).
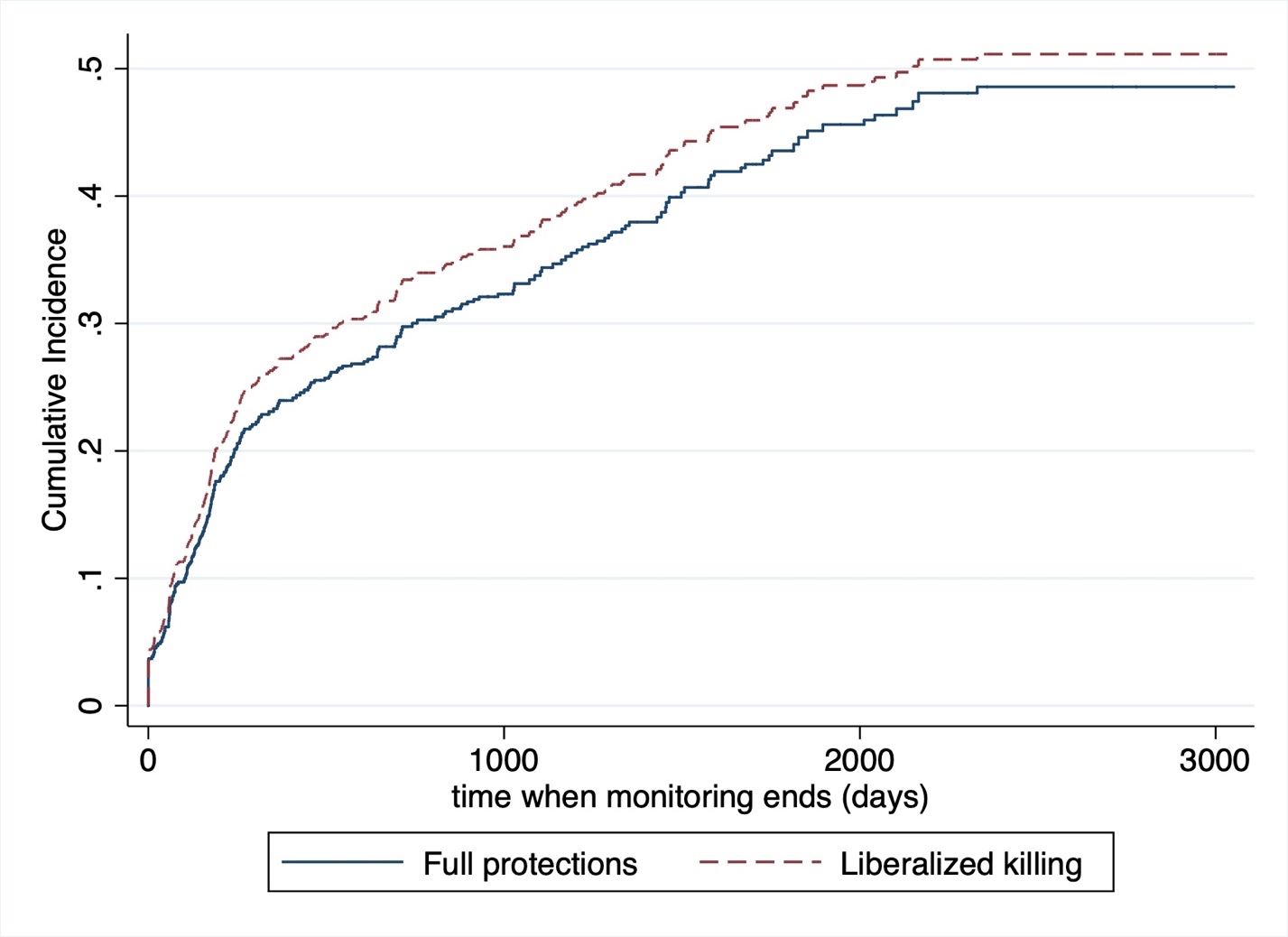


Supplementary Fig. S3. Cumulative incidence functions (CIFs) for the *LTF* endpoint (n=243) by policy period, derived from the stratified joint Cox model M5, for MAIN simulation scenario (see *Supplemental Text*). We illustrate the cumulative incidence (y axis, proportion of individuals reaching end of monitoring [endpoint]) over time (x axis) for periods of full protections (navy, solid lines) and periods of liberalized killing (maroon, dashed lines).


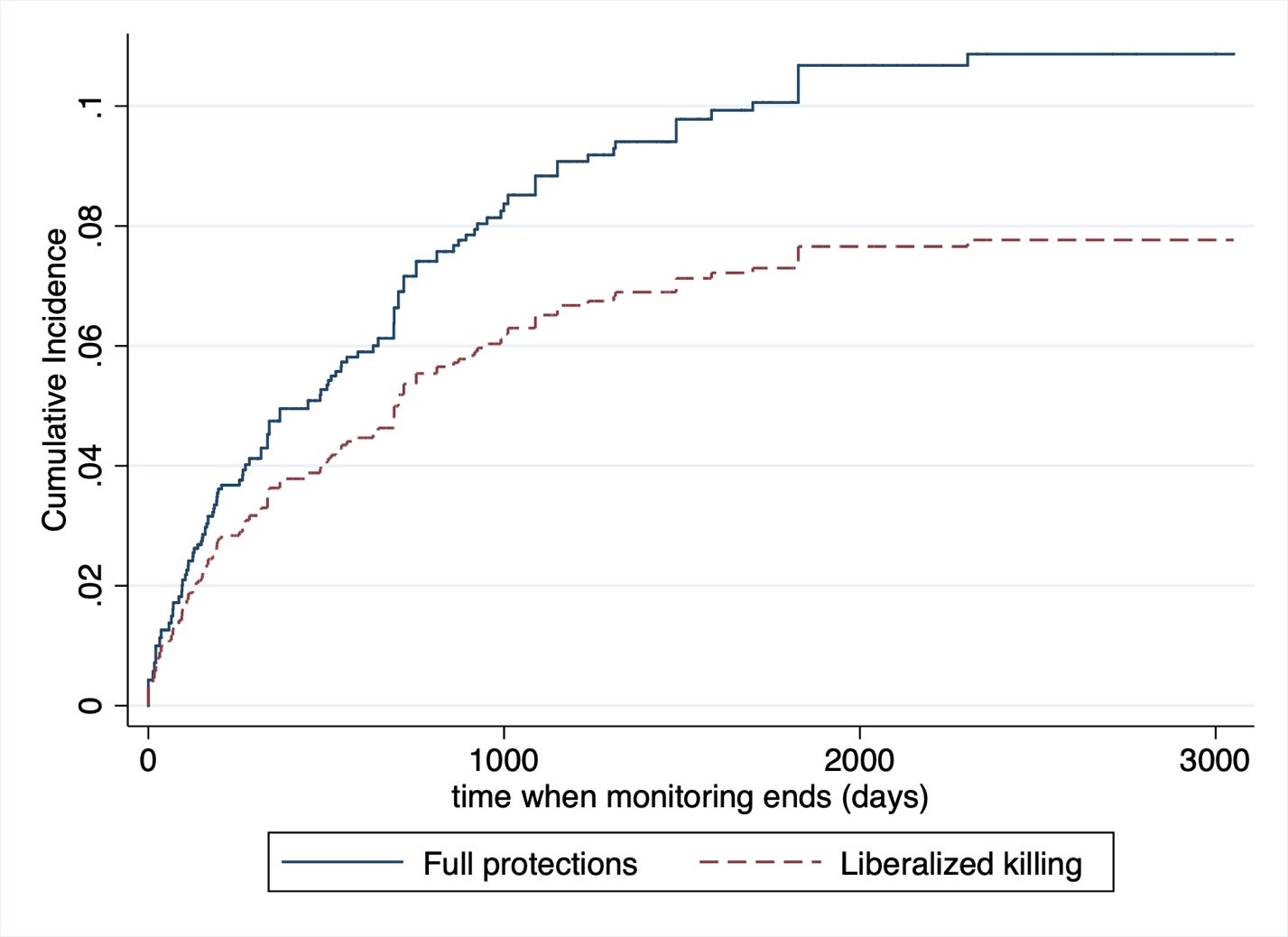


Supplementary Fig. S4. Cumulative incidence functions (CIFs) for the *reported poached* endpoint (n=88) by policy period, derived from the stratified joint Cox model M5. We illustrate the cumulative incidence (y axis, proportion of individuals reaching end of monitoring [endpoint]) over time (x axis) for periods of full protections (navy, solid lines) and periods of liberalized killing (maroon, dashed lines).


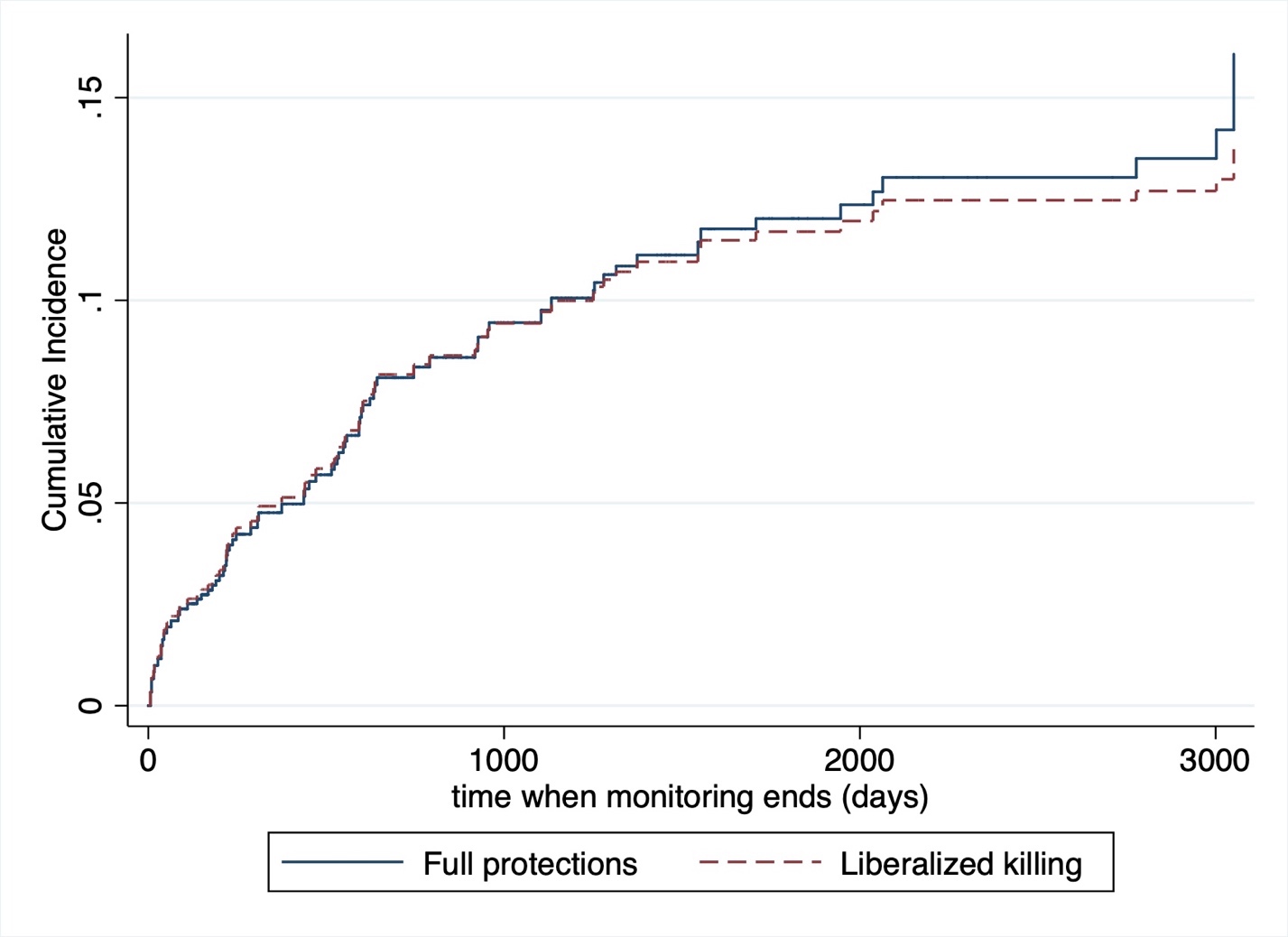


Supplementary Fig. S5. Cumulative incidence functions (CIFs) for the *nonhuman* endpoint (n=77) by policy period, derived from the stratified joint Cox model M5. We illustrate the cumulative incidence (y axis, proportion of individuals reaching end of monitoring [endpoint]) over time (x axis) for periods of full protections (navy, solid lines) and periods of liberalized killing (maroon, dashed lines).


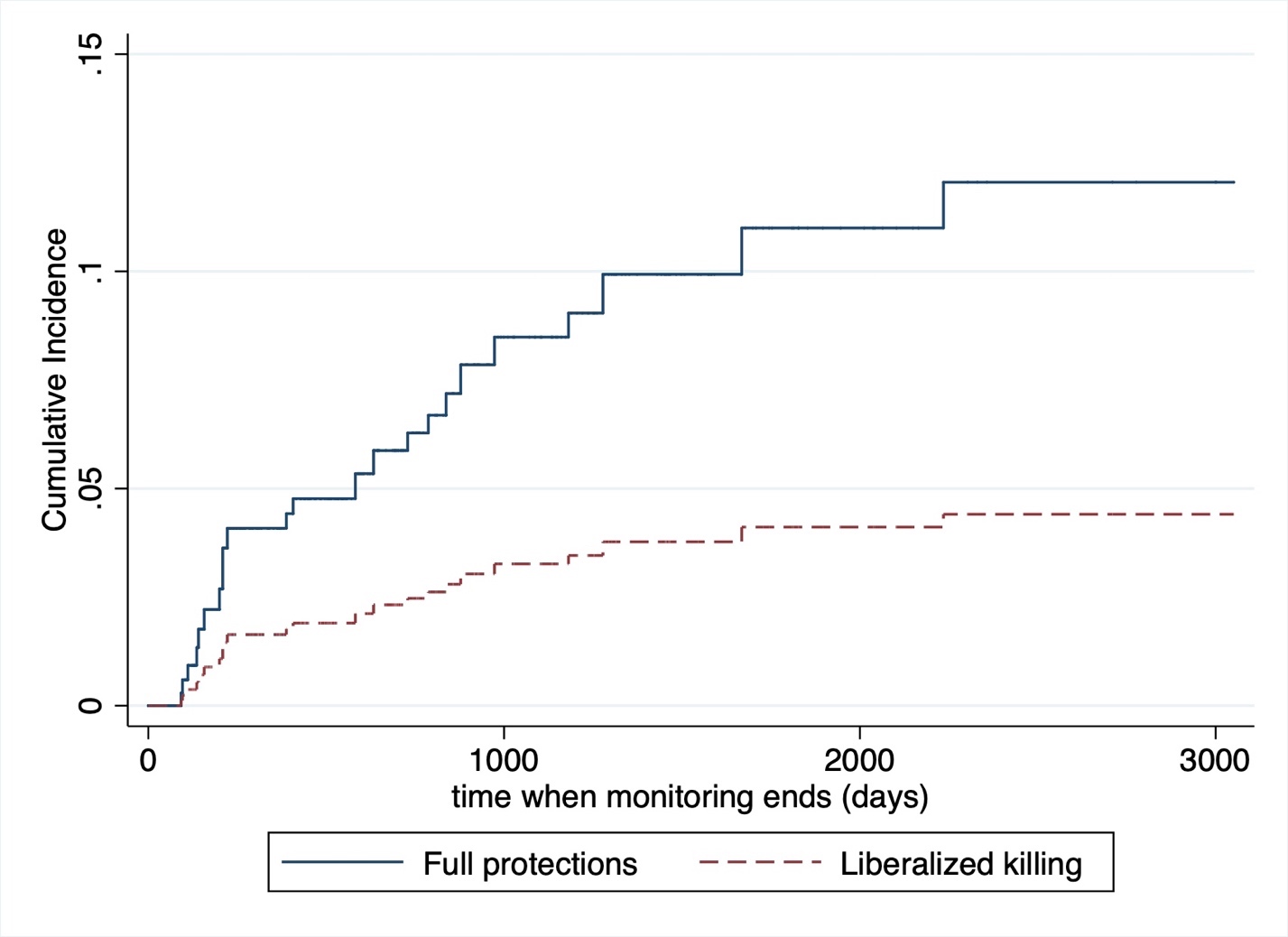


Supplementary Fig. S6. Cumulative incidence functions (CIFs) for the *collision* endpoint (n=24) by policy period, derived from the stratified joint Cox model M5. We illustrate the cumulative incidence (y axis, proportion of individuals reaching end of monitoring [endpoint]) over time (x axis) for periods of full protections (navy, solid lines) and periods of liberalized killing (maroon, dashed lines).


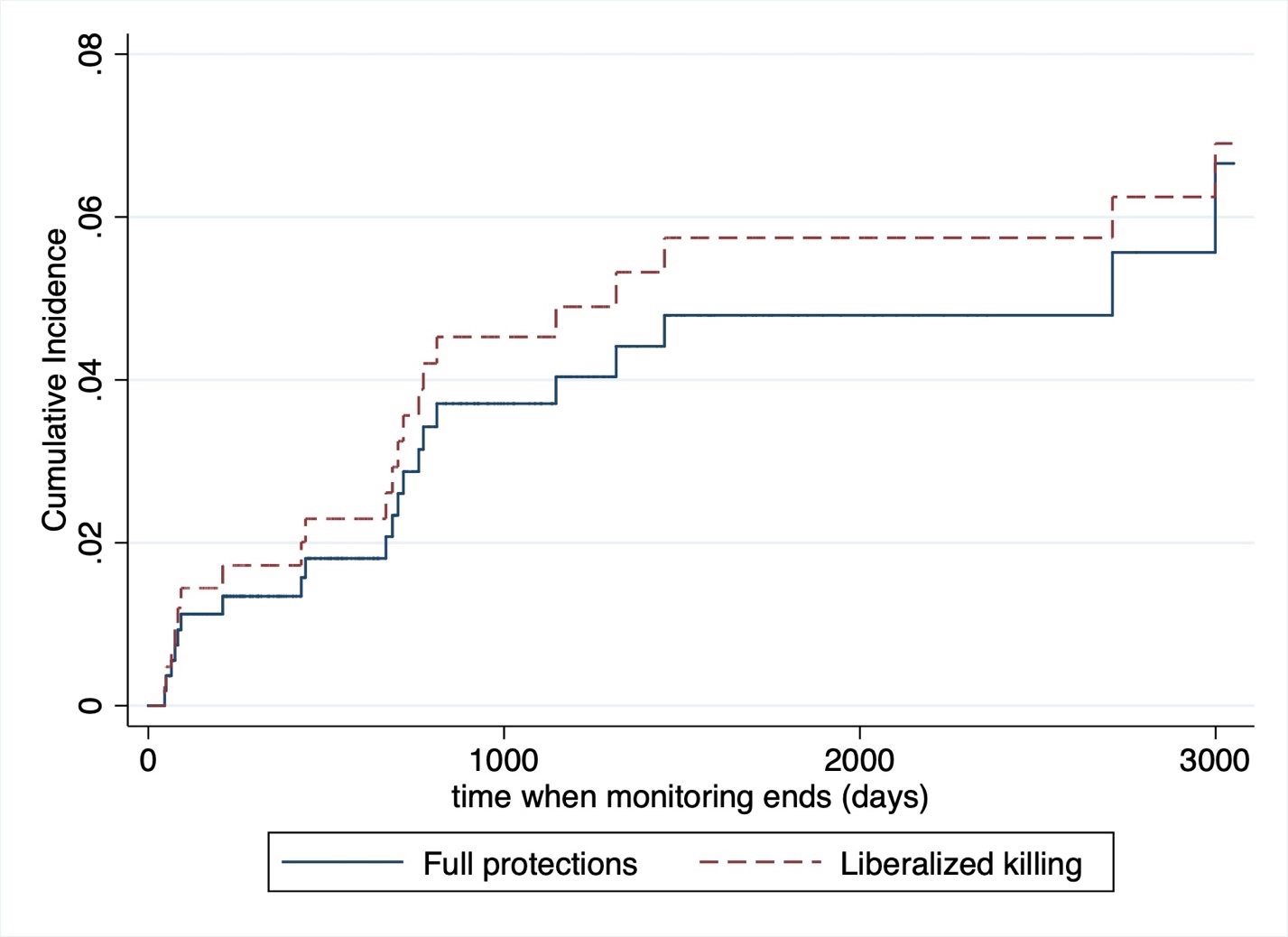


Supplementary Fig. S7. Cumulative incidence functions (CIFs) for the *uncertain* endpoint (n=21) by policy period, derived from the stratified joint Cox model M5. We illustrate the cumulative incidence (y axis, proportion of individuals reaching end of monitoring [endpoint]) over time (x axis) for periods of full protections (navy, solid lines) and periods of liberalized killing (maroon, dashed lines).


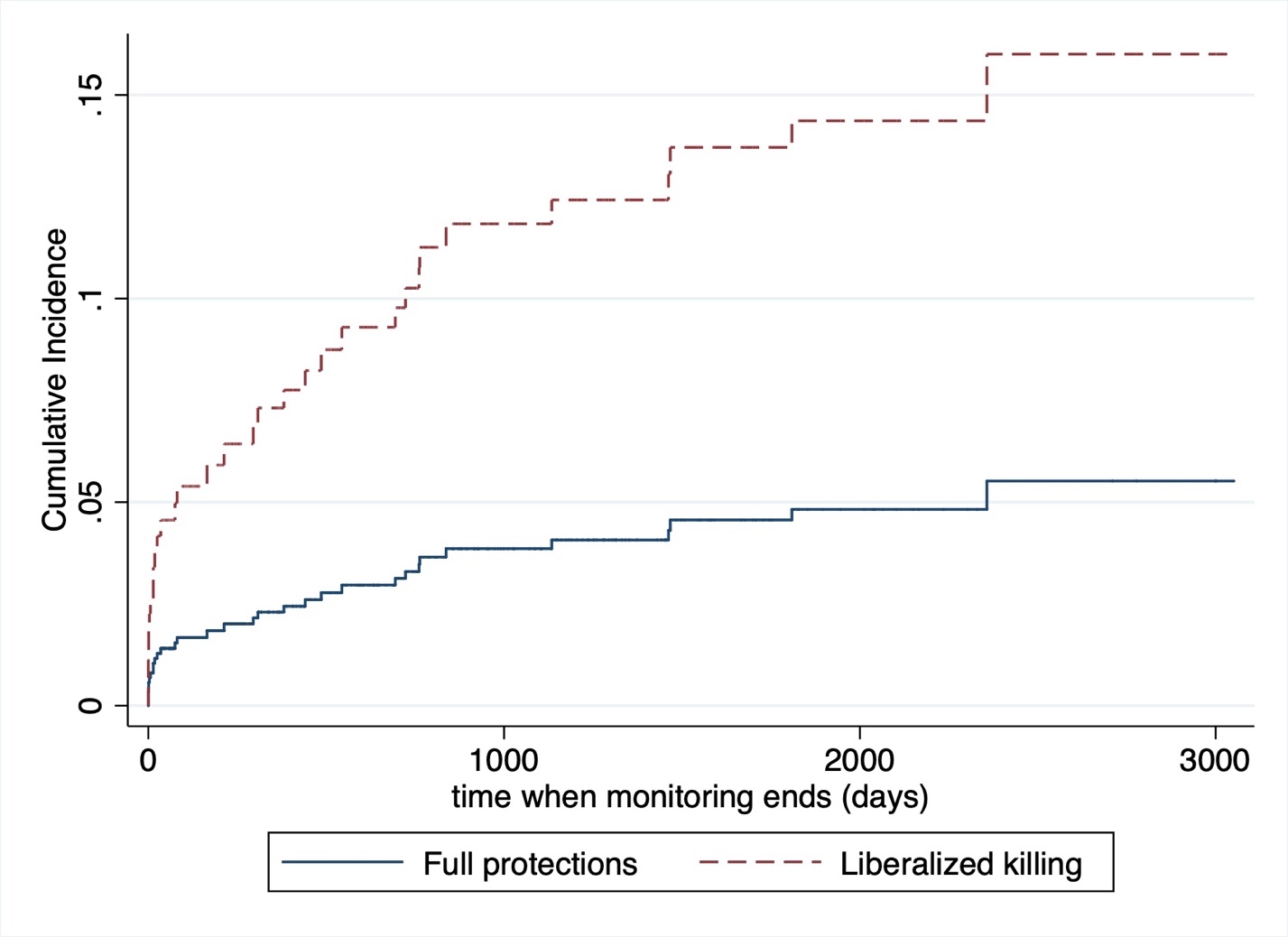


Supplementary Fig. S8. Cumulative incidence functions (CIFs) for the *legal killing* endpoint (n=32) by policy period, derived from the stratified joint Cox model M5. We illustrate the cumulative incidence (y axis, proportion of individuals reaching end of monitoring [endpoint]) over time (x axis) for periods of full protections (navy, solid lines) and periods of liberalized killing (maroon, dashed lines).


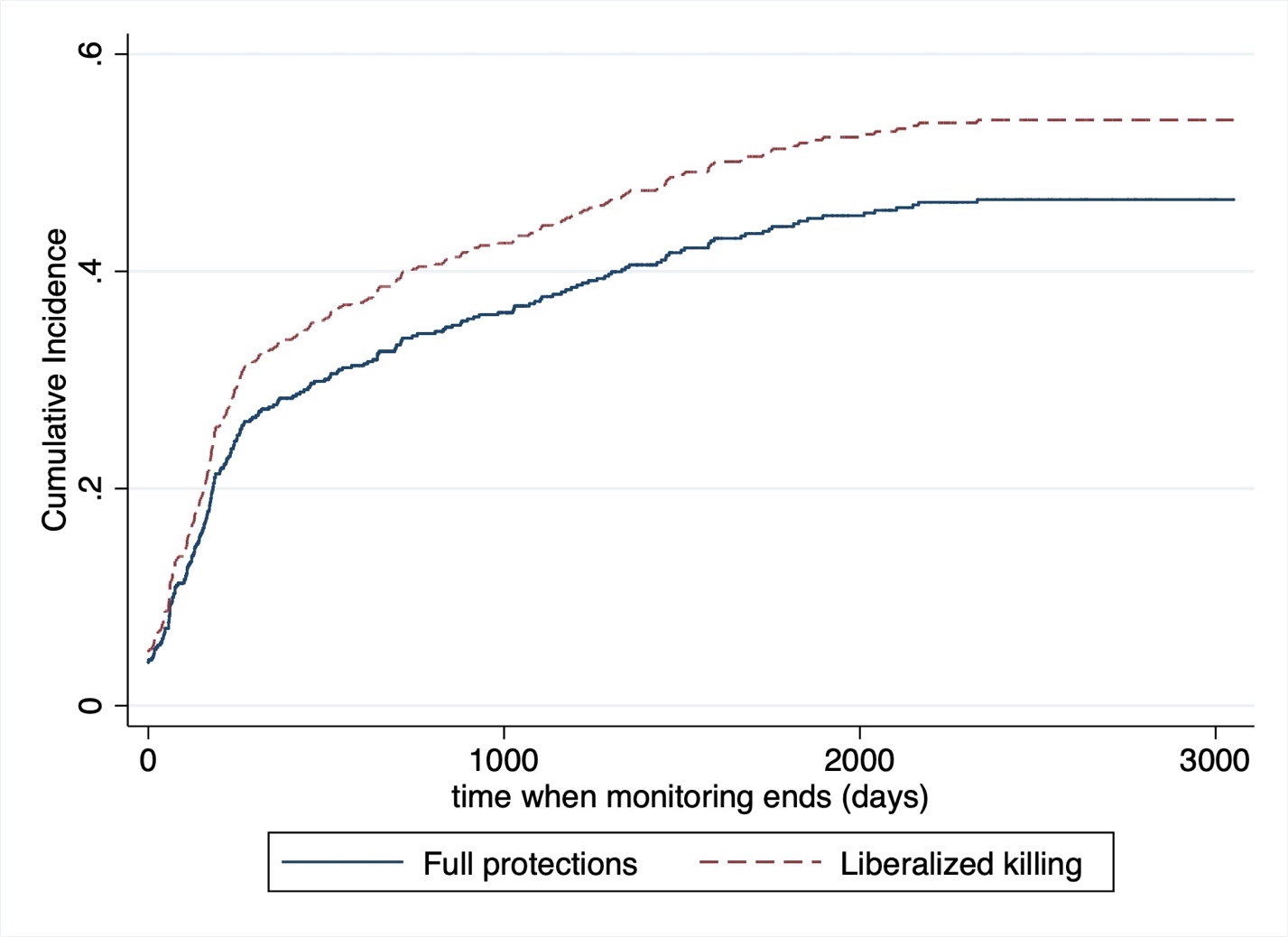


Supplementary Fig. S9. Cumulative incidence functions (CIFs) for the *LTF* endpoint (n=243) by policy period, derived from FG subhazard models, for MAIN simulation scenario. We illustrate the cumulative incidence (y axis, proportion of individuals reaching end of monitoring [endpoint]) over time (x axis) for periods of full protections (navy, solid lines) and periods of liberalized killing (maroon, dashed lines).


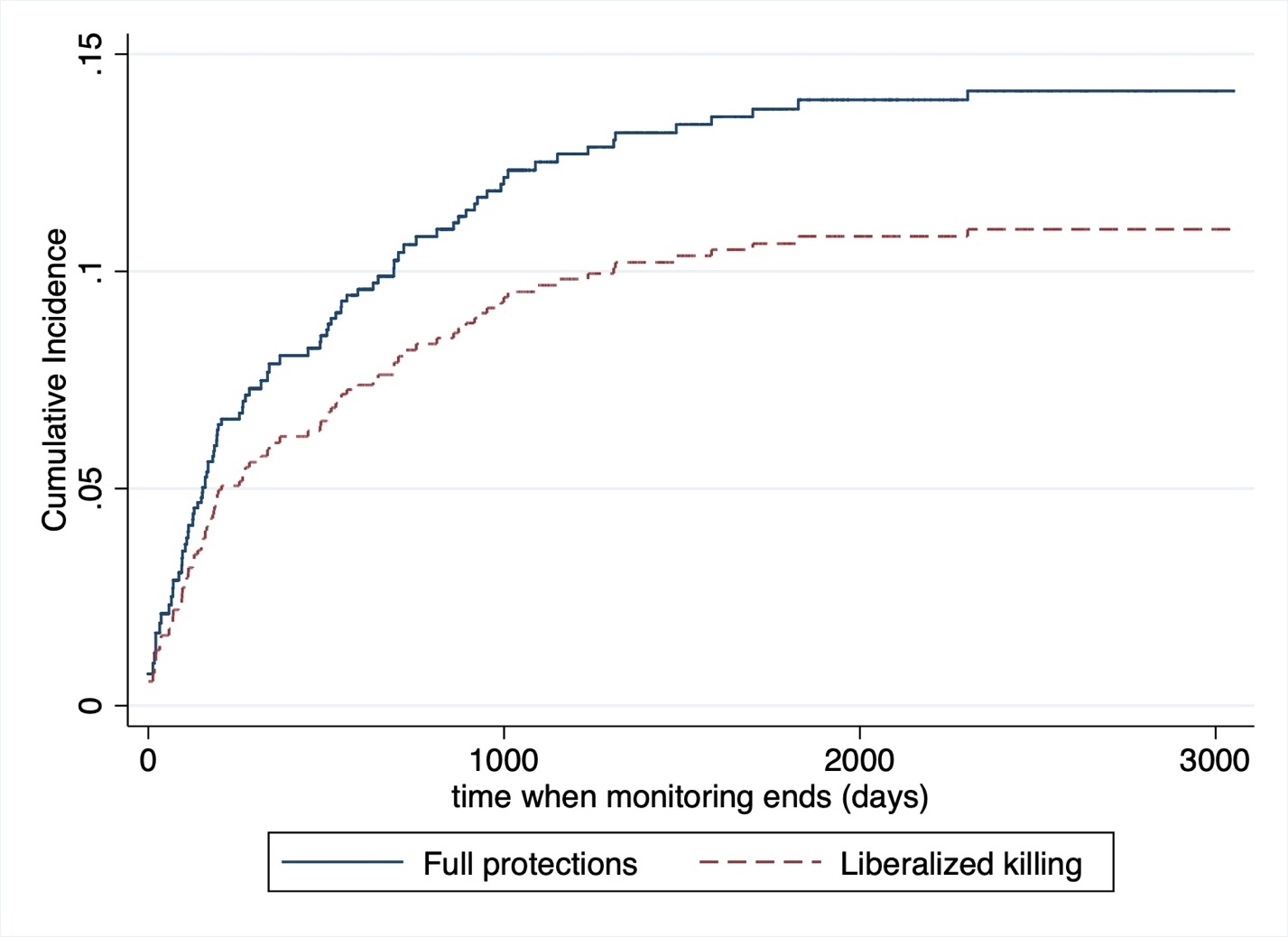


Supplementary Fig. S10. Cumulative incidence functions (CIFs) for the *reported poached* endpoint (n=88) by policy period, derived from FG subhazard models. We illustrate the cumulative incidence (y axis, proportion of individuals reaching end of monitoring [endpoint]) over time (x axis) for periods of full protections (navy, solid lines) and periods of liberalized killing (maroon, dashed lines).


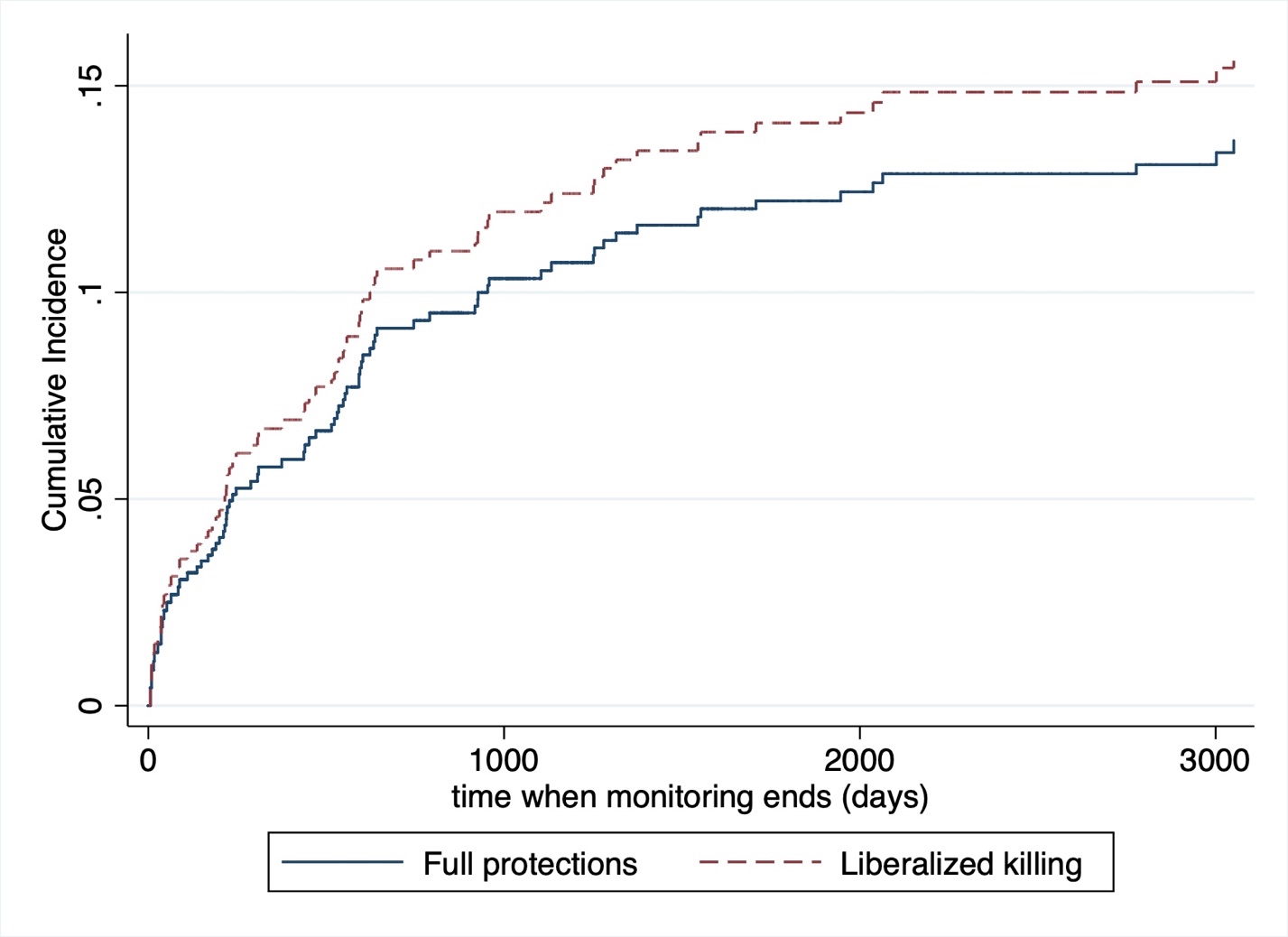


Supplementary Fig. S11. Cumulative incidence functions (CIFs) for the *nonhuman* endpoint (n=77) by policy period, derived from FG subhazard models. We illustrate the cumulative incidence (y axis, proportion of individuals reaching end of monitoring [endpoint]) over time (x axis) for periods of full protections (navy, solid lines) and periods of liberalized killing (maroon, dashed lines).


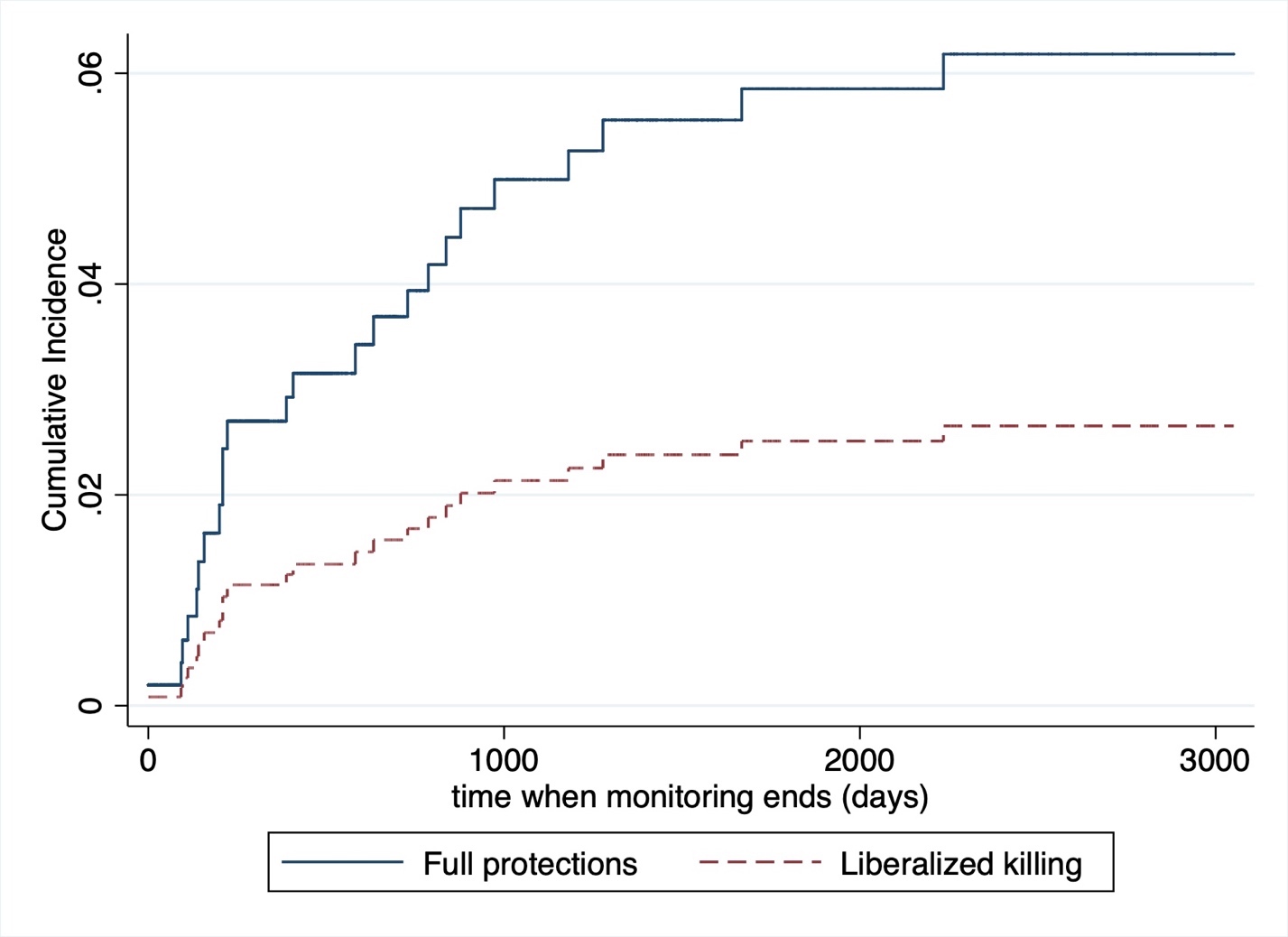


Supplementary Fig. S12. Cumulative incidence functions (CIFs) for the *collision* endpoint (n=24) by policy period, derived from FG subhazard models. We illustrate the cumulative incidence (y axis, proportion of individuals reaching end of monitoring [endpoint]) over time (x axis) for periods of full protections (navy, solid lines) and periods of liberalized killing (maroon, dashed lines).


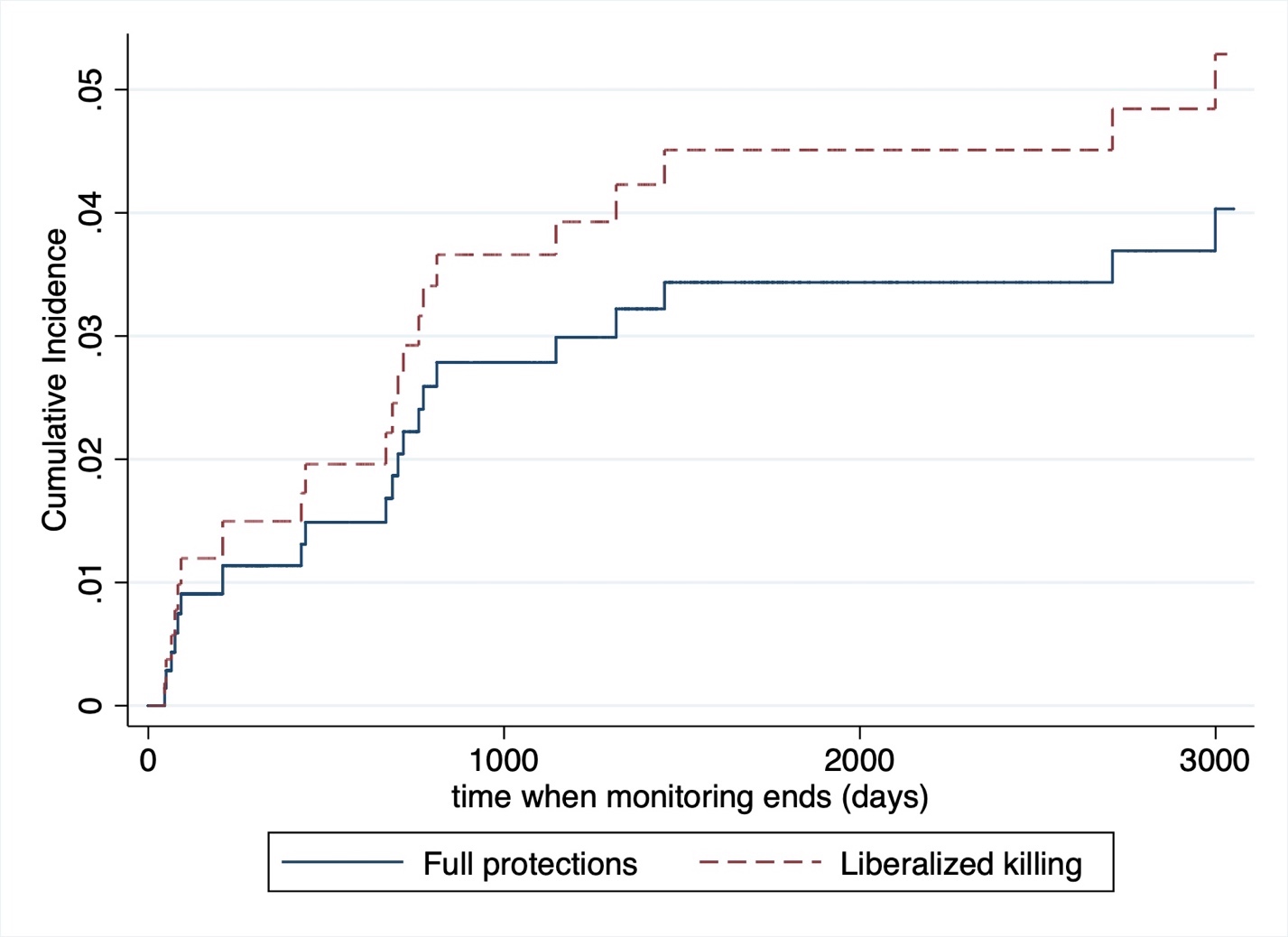


Supplementary Fig. S13. Cumulative incidence functions (CIFs) for the *uncertain* endpoint (n=21) by policy period, derived from FG subhazard models. We illustrate the cumulative incidence (y axis, proportion of individuals reaching end of monitoring [endpoint]) over time (x axis) for periods of full protections (navy, solid lines) and periods of liberalized killing (maroon, dashed lines).


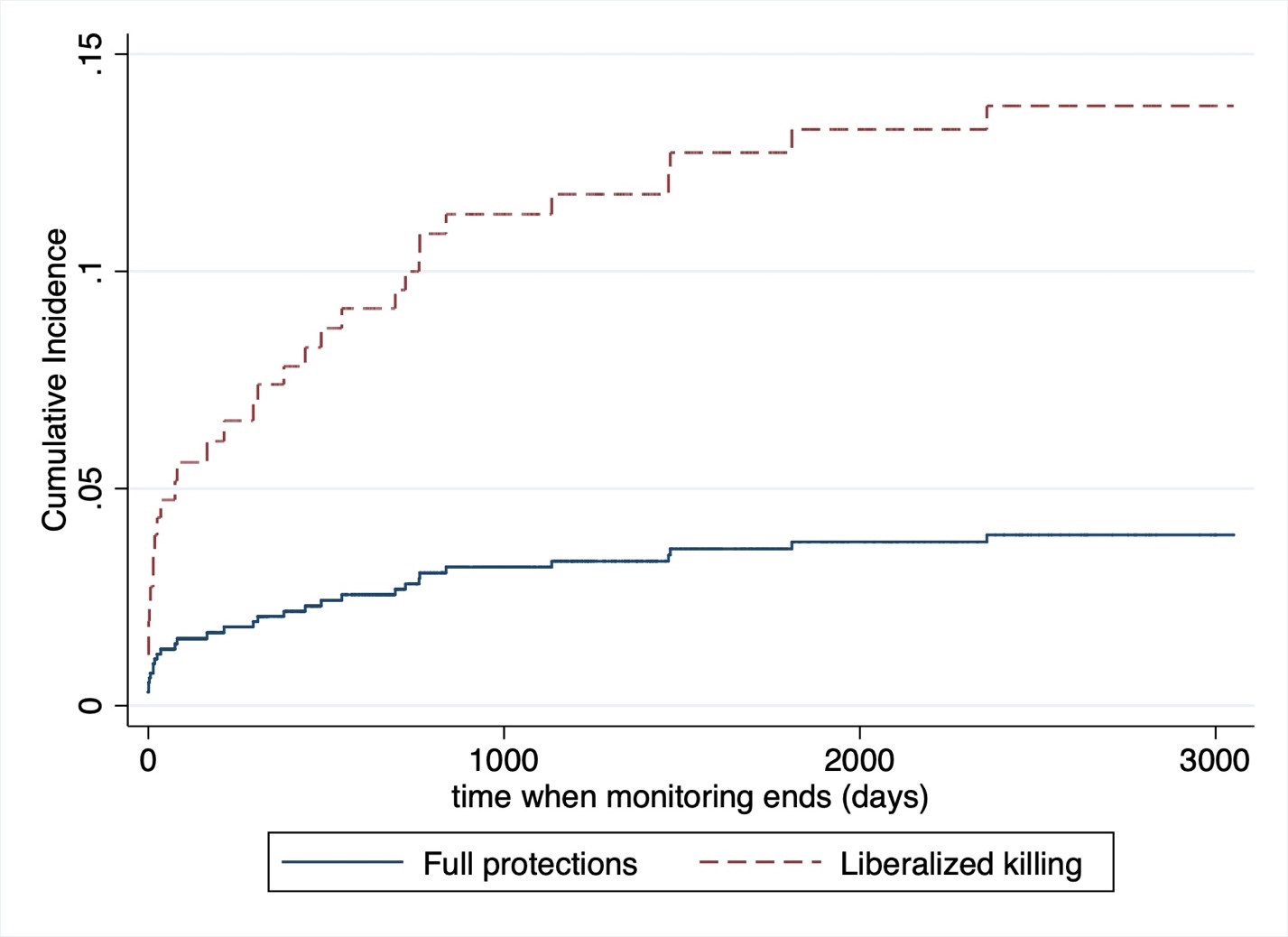


Supplementary Fig. S14. Cumulative incidence functions (CIFs) for the *legal killing* endpoint (n=32) by policy period, derived from FG subhazard models. We illustrate the cumulative incidence (y axis, proportion of individuals reaching end of monitoring [endpoint]) over time (x axis) for periods of full protections (navy, solid lines) and periods of liberalized killing (maroon, dashed lines).


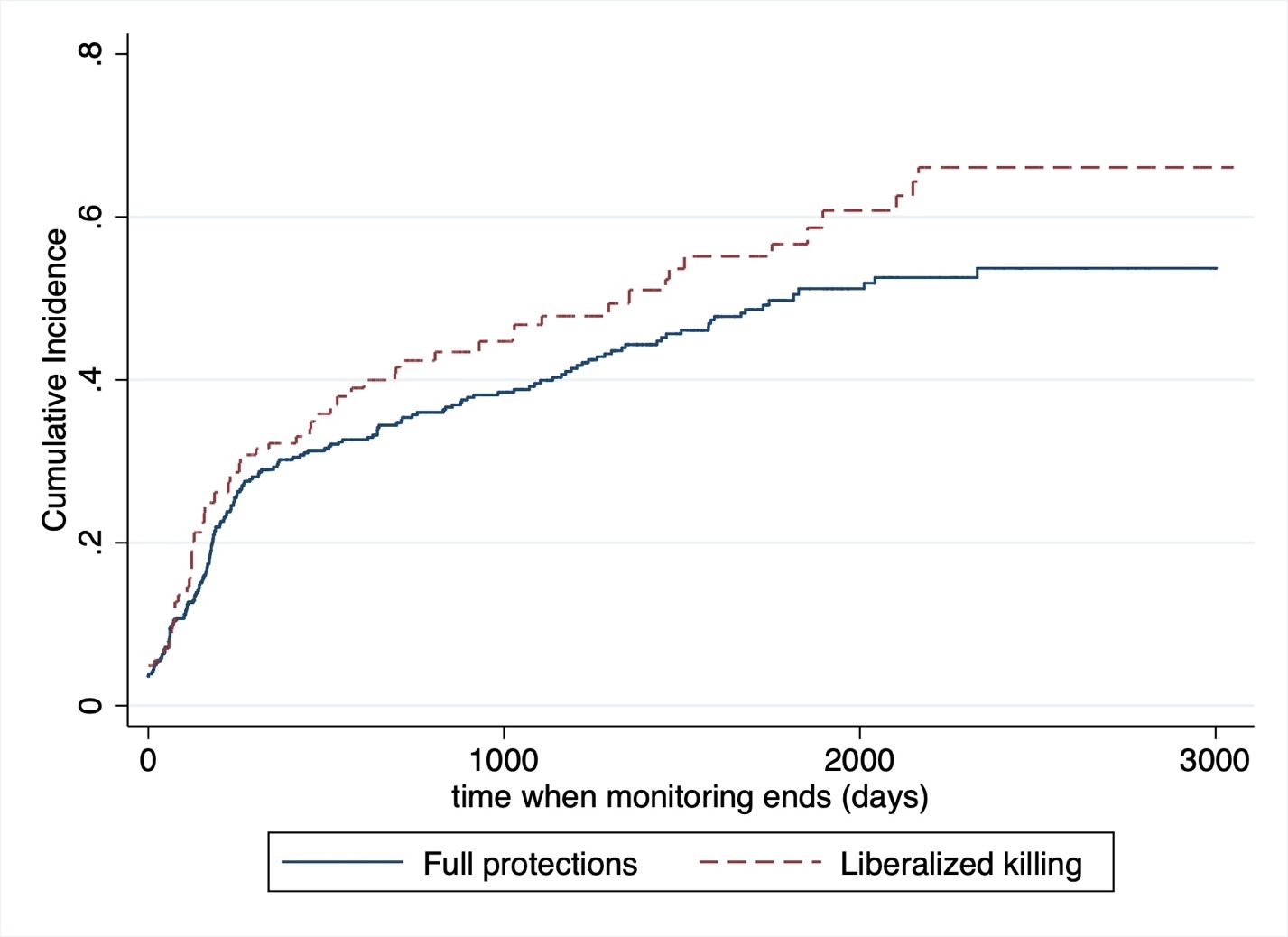


Supplementary Fig. S15. Cumulative incidence functions (CIFs) for the *LTF* endpoint (n=243) by policy period, derived from non-parametric models, for MAIN simulation scenario. We illustrate the cumulative incidence (y axis, proportion of individuals reaching end of monitoring [endpoint]) over time (x axis) for periods of full protections (navy, solid lines) and periods of liberalized killing (maroon, dashed lines).


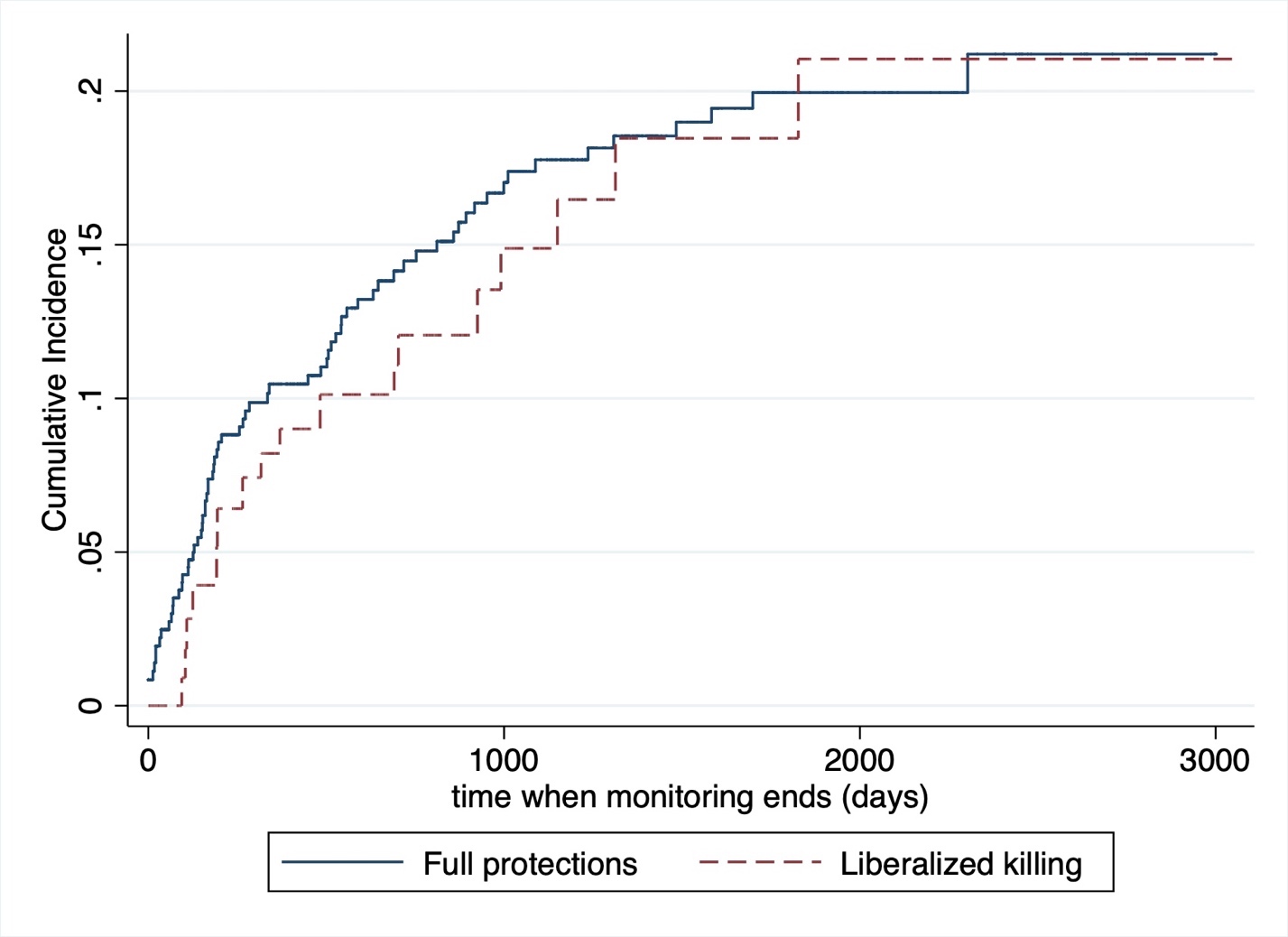


Supplementary Fig. S16. Cumulative incidence functions (CIFs) for the *reported poached* endpoint (n=88) by policy period, derived from non-parametric models. We illustrate the cumulative incidence (y axis, proportion of individuals reaching end of monitoring [endpoint]) over time (x axis) for periods of full protections (navy, solid lines) and periods of liberalized killing (maroon, dashed lines).


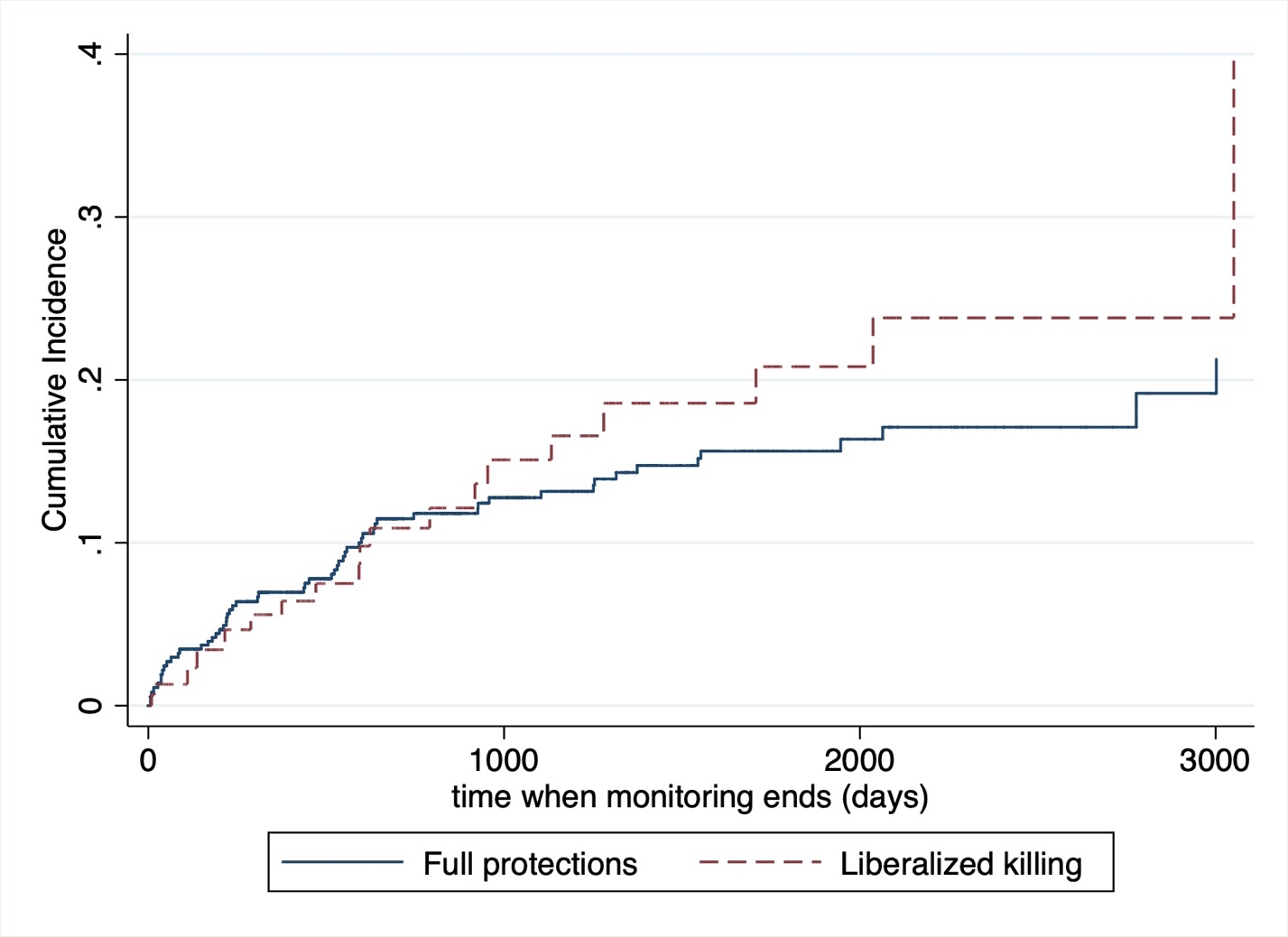


Supplementary Fig. S17. Cumulative incidence functions (CIFs) for the *nonhuman* endpoint (n=77) by policy period, derived from non-parametric models. We illustrate the cumulative incidence (y axis, proportion of individuals reaching end of monitoring [endpoint]) over time (x axis) for periods of full protections (navy, solid lines) and periods of liberalized killing (maroon, dashed lines).


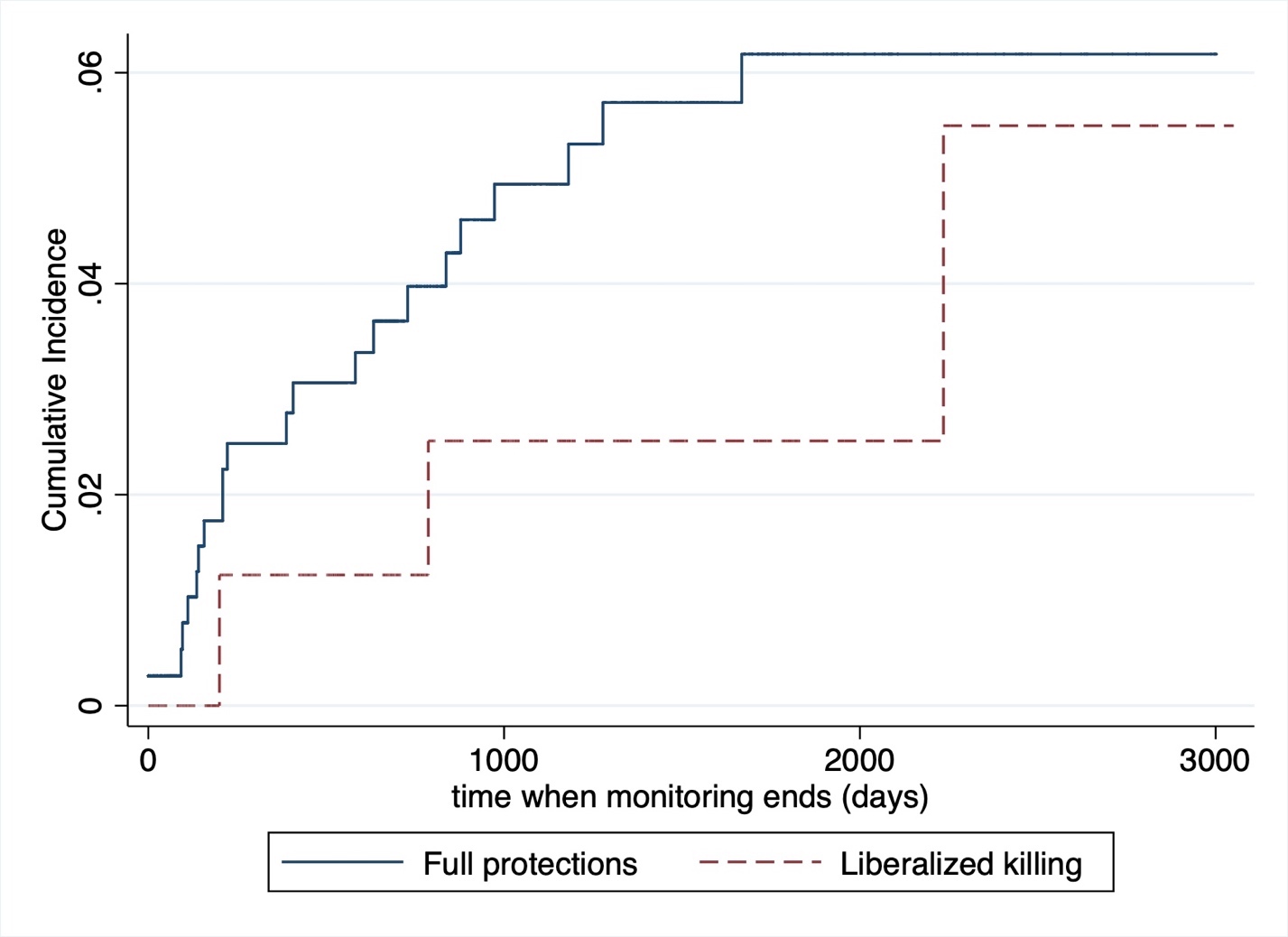


Supplementary Fig. S18. Cumulative incidence functions (CIFs) for the *collision* endpoint (n=24) by policy period, derived from non-parametric models. We illustrate the cumulative incidence (y axis, proportion of individuals reaching end of monitoring [endpoint]) over time (x axis) for periods of full protections (navy, solid lines) and periods of liberalized killing (maroon, dashed lines).


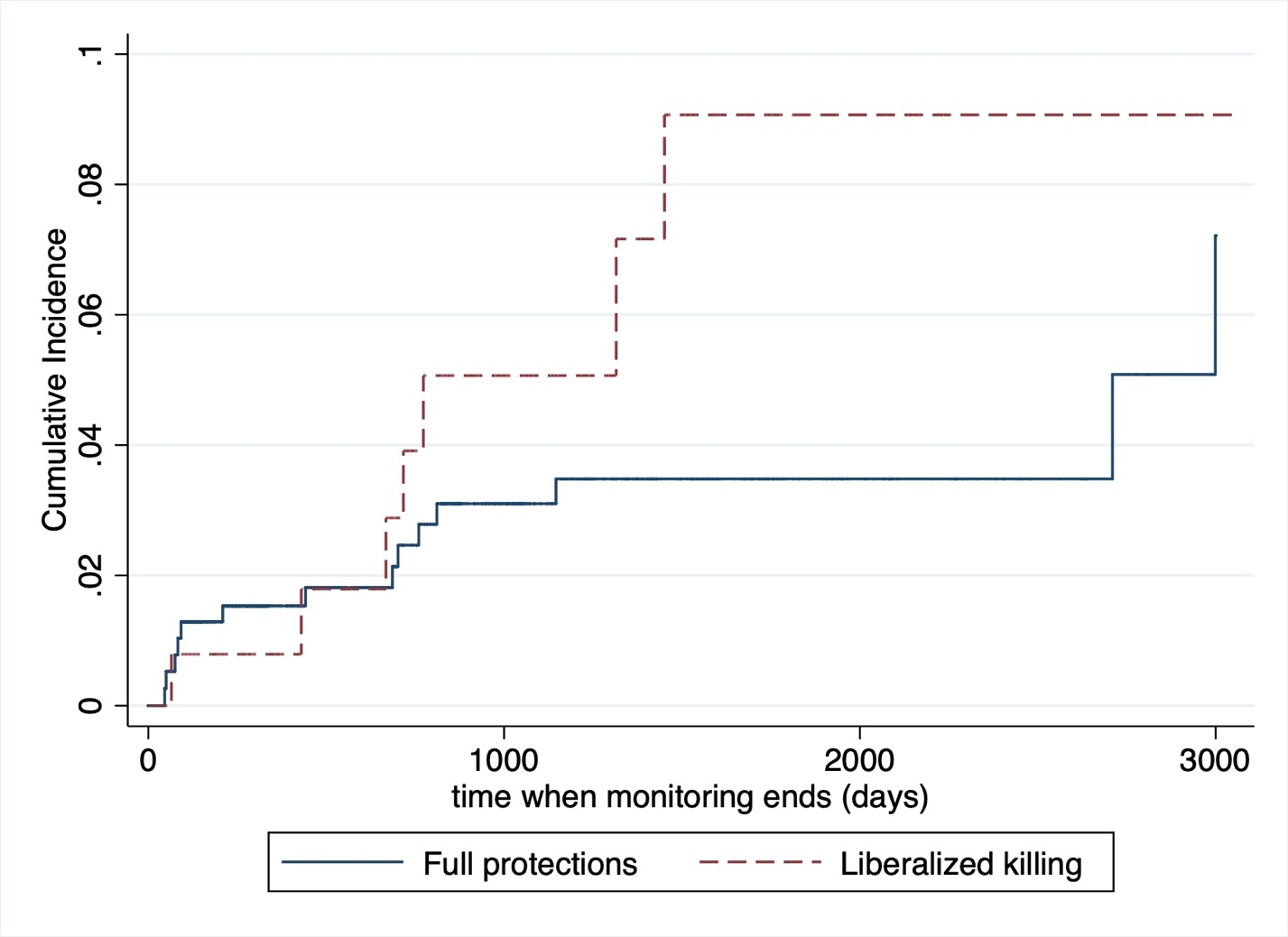


Supplementary Fig. S19. Cumulative incidence functions (CIFs) for the *uncertain* endpoint (n=21) by policy period, derived from non-parametric models. We illustrate the cumulative incidence (y axis, proportion of individuals reaching end of monitoring [endpoint]) over time (x axis) for periods of full protections (navy, solid lines) and periods of liberalized killing (maroon, dashed lines).


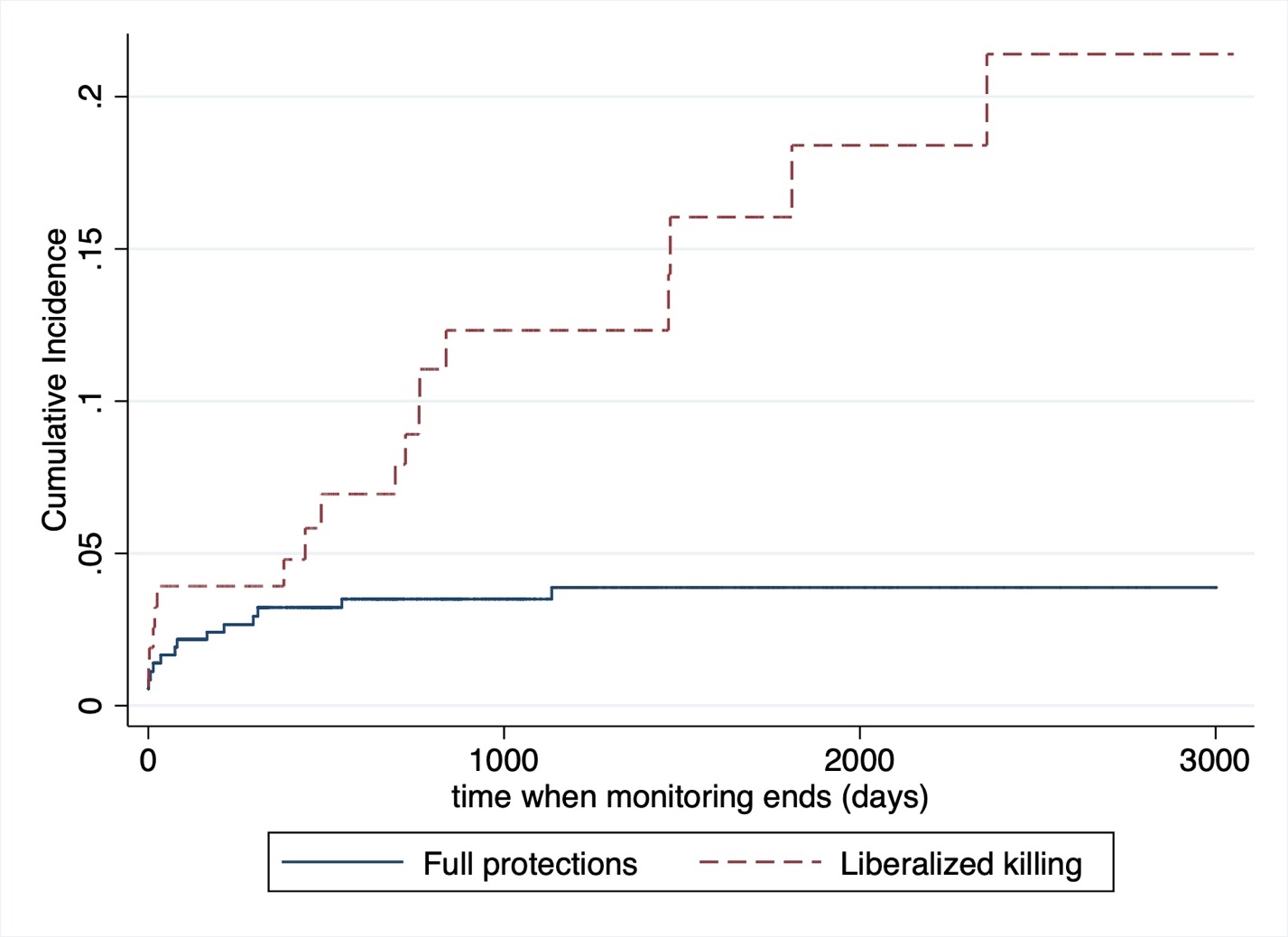


Supplementary Fig. S20. Cumulative incidence functions (CIFs) for the *legal killing* endpoint (n=32) by policy period, derived from non-parametric models. We illustrate the cumulative incidence (y axis, proportion of individuals reaching end of monitoring [endpoint]) over time (x axis) for periods of full protections (navy, solid lines) and periods of liberalized killing (maroon, dashed lines).


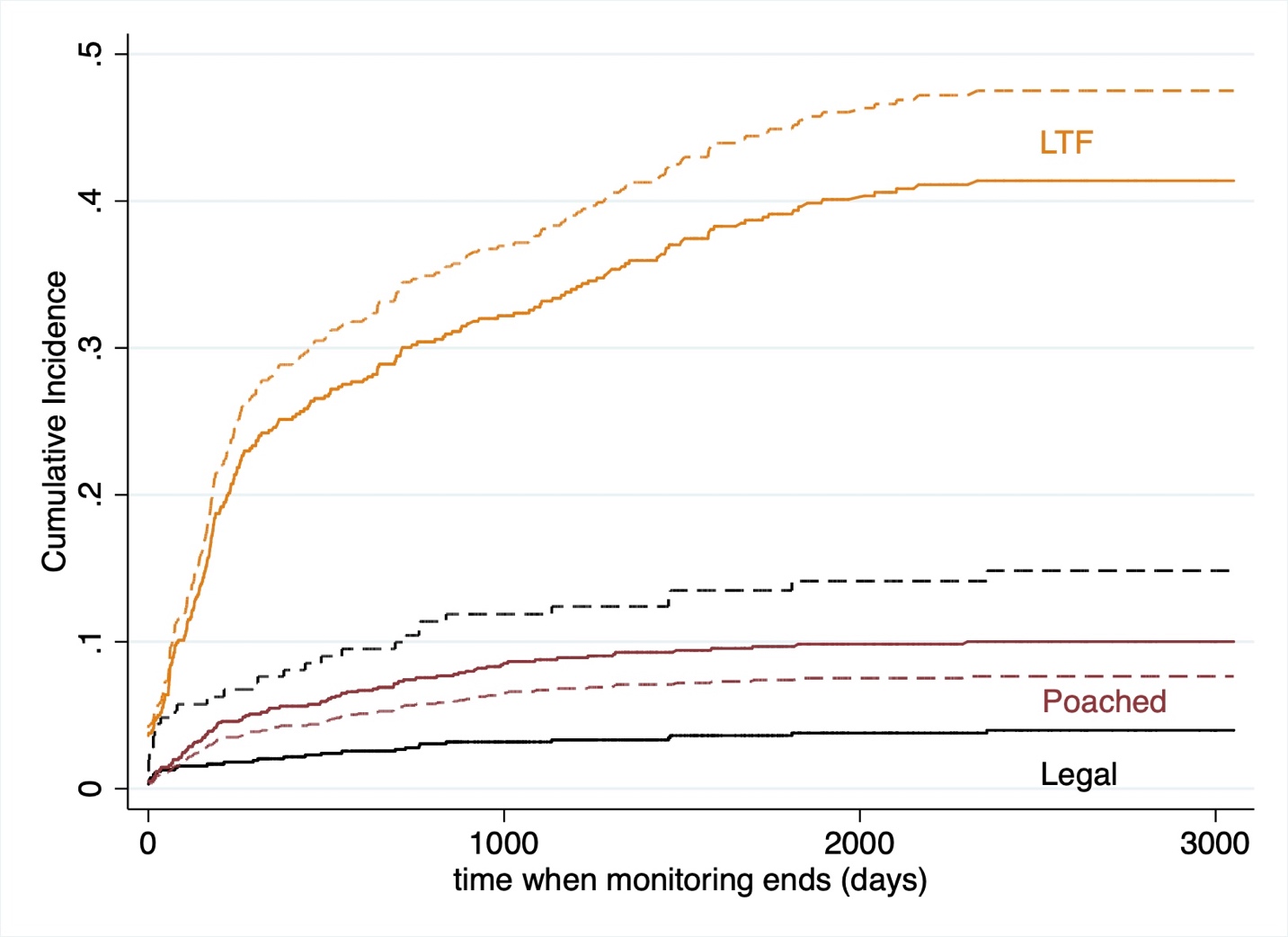


Supplementary Fig. S21. Cumulative incidence function (CIFs) of events for *LTF* (n=243, orange), *reported poached* (n=88, maroon), and *legal killing* (n=32, black) endpoints derived from FG models for LOW imputation scenario. For each endpoint, we illustrate the cumulative incidence (y axis, proportion of individuals reaching end of monitoring [endpoint]) over time (x axis) for periods of full protections (navy, solid lines) and periods of liberalized killing (maroon, dashed lines).


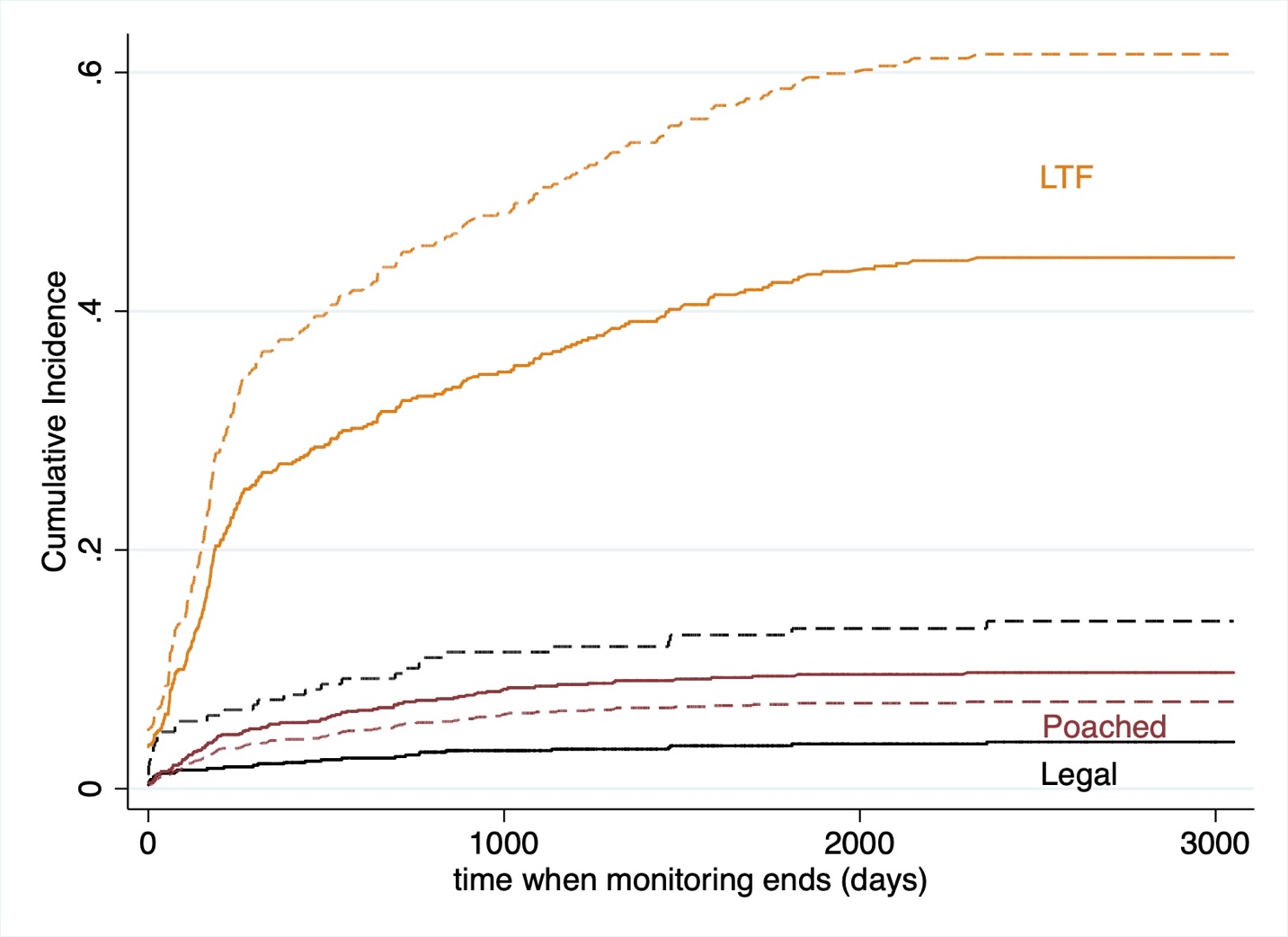


Supplementary Fig. S22. Cumulative incidence function (CIFs) of events for *LTF* (n=251, orange), *reported poached* (n=88, maroon), and *legal killing* (n=32, black) endpoints derived from FG models for HIGH imputation scenario. For each endpoint, we illustrate the cumulative incidence (y axis, proportion of individuals reaching end of monitoring [endpoint]) over time (x axis) for periods of full protections (navy, solid lines) and periods of liberalized killing (maroon, dashed lines).

Supplementary Tables S1 to S9

| **wolf_ID** | **analysis time when spell begins** | **analysis time when spell ends** | **lib_kill** | **winter** | **method_change** | **cause_endpoint** |
| --- | --- | --- | --- | --- | --- | --- |
| 410 | 0 | 55 | 0 | 0 | 2 |  |
| 410 | 55 | 350 | 1 | 0 | 3 |  |
| 410 | 350 | 515 | 0 | 1 | 3 | 6 |

**Supplementary Table S1.** Example of a hypothetical wolf’s (identified by *wolf_ID* 410) monitoring history broken up into mutually exclusive spells for each wolf. The duration of each spell is less than or equal to the duration of the covariate period it overlaps, for the creation of time-dependent variables. The analysis time variables keep track of the time interval and order of spells, and each spell corresponds to a change in a variable (either *lib_kill* or *winter*, in this example). The *cause_endpoint* variable is only reflected for the last record, which corresponds to when the endpoint was reached (at T=515 days). *Note*: *lib_kill* = ‘1’ for liberalized killing, ‘0’ full protections; *winter* = ‘1’ if winter, ‘0’ summer; *method_change* = ‘1’ if 1979-1994, ‘2’ if 1995-2000 and ‘3’ if 2001-2012; *cause_endpoint* ‘6’ corresponds to reported poached.

| **Period start (dd/mm/yyyy)** | **Period end (dd/mm/yyyy)** | **Federal status** | **Policy period****  **(*lib_kill*)** | **Number of wolves Lost to follow-up (LTF) in WI** |
| --- | --- | --- | --- | --- |
| 15/04/1994 | 31/03/2003 | Listed as endangered | full protections (0) | 119 |
| 01/04/2003 | 30/01/2005 | Down-listed to threatened | liberalized killing (1) | 30 |
| 31/01/2005 | 31/03/2005 | Relisted | full protections (0) | 1 |
| 01/04/2005 | 13/09/2005 | Sub-permit for killing issued | liberalized killing (1) | 5 |
| 14/09/2005 | 23/04/2006 | Sub-permit rescinded | full protections (0) | 4 |
| 24/04/2006* | 31/07/2006 | Sub-permit for killing issued | liberalized killing (1) | 2 |
| 01/08/2006 | 11/03/2007 | Sub-permit rescinded | full protections (0) | 12 |
| 12/03/2007 | 28/09/2008 | Delisted | liberalized killing (1) | 13 |
| 29/09/2008 | 03/05/2009 | Relisted | full protections (0) | 10 |
| 04/05/2009 | 30/06/2009 | Delisted | liberalized killing (1) | 0 |
| 01/07/2009 | 26/01/2012 | Relisted | full protections (0) | 34 |
| 27/01/2012 | 14/04/2012 | Delisted | liberalized killing (1) | 1 |

**Supplementary Table S2.** Periods of wolf policy changes in Wisconsin and Michigan during our study period ^12,26^, with number of LTF wolves in original dataset (n=231) by policy period in Wisconsin. States are identical except for the sub-permit issuance on 6 May 2006 to Michigan instead of issuance on 24 April 2006 to Wisconsin ^26^*.* ** Killing a wolf that posed a threat to human safety was always allowed under ESA sec.11(a)(3)

|  | **Imputation Models (IMs)** | | | | | | | | | |
| --- | --- | --- | --- | --- | --- | --- | --- | --- | --- | --- |
|  | **IM 1** | | **IM 2** | | **IM 3** | | **IM 4** | | **IM 5** | |
| **Variable** | SHR | 95% CI | SHR | 95% CI | SHR | 95% CI | SHR | 95% CI | SHR | 95% CI |
| *Liberalized killing periods (lib_kill)* | 0.96 | 0.69-0.13 | 1.02 | 0.73-1.45 | 1.13 | 0.77-1.66 | 1.23 | 0.81-1.87 | 0.99 | 0.7-1.4 |
| *Winter periods (winter)* | 1 | - | 1.51 | 1.07-2.13 | 1.55 | 1.10-2.20 | 3.2 | 2.09-4.90 | 3.11 | 2.03-4.77 |
| *Census periods (method_change)* |  |  |  |  |  |  |  |  |  |  |
| *Census method 1* | 1 | - | 1 | - | 1 | - | 1 | - | 1 | - |
| *Census method 2* | 1 | - | 1 | - | 1.12 | 0.72-1.74 | 1 | - | 1 | - |
| *Census method 3* | 1 | - | 1 | - | 0.86 | 0.59-1.25 | 1 | - | 1 | - |
| *time-varying coefficient (tvc)* |  |  |  |  |  |  |  |  |  |  |
| *Liberalized killing periods (lib_kill tvc) (change per year)* | 1 | - | 1 | - | 1 | - | 0.69 | 0.69-1 | 1 | - |
| *Winter periods (winter tvc) (change per year)* | 1 | - | 1 | - | 1 | - | 0.48 | 0.48-0.69 | 0.69 | 0.48-0.69 |
| **Model statistics** |  |  |  |  |  |  |  |  |  |  |
| Log likelihood (model) | -1364.06 | | -1360.411 | | -1359.295 | | -1344.444 | | -1345.736 | |
| df | 1 | | 2 | | 4 | | 4 | | 3 | |
| AIC | 2730.121 | | 2724.821 | | 2726.59 | | 2696.888 | | 2697.471 | |
| BIC | 2735.914 | | 2736.408 | | 2749.764 | | 2720.062 | | 2714.852 | |

Supplementary Table S3. Fine & Gray (FG) subhazard imputation models (IMs) estimated for imputation of probability of LTF for wolves with missing endpoint (n=26 wolves). We present model statistics, along with subhazard ratios (SHR) and confidence intervals (95% CI) for all variables in all models. IMs 4 and 5 were the most appropriate models. IM2 (corresponding to IM4 or IM5 without tvcs) was used for LTF prediction and imputation. See Supplementary Table S1 for variable names and coding.

| **Wolf ID** | **days to endpoint** | **P(ltf) \| t; lib_kill=1, winter=1** |
| --- | --- | --- |
| 788 | 115 | 0.179 |
| 766/765 | 151 | 0.217 |
| 825 | 158 | 0.226 |
| 815 | 159 | 0.226 |
| 756 | 164 | 0.232 |
| 789 | 176 | 0.255 |
| 807 | 211 | 0.291 |
| 819 | 303 | 0.356 |
| 665 | 320 | 0.364 |
| 769 | 339 | 0.364 |
| 690 | 531 | 0.408 |
| 791 | 537 | 0.410 |
| 776 | 654 | 0.433 |
| 755 | 693 | 0.433 |
| 773 | 696 | 0.433 |
| 760 | 708 | 0.436 |
| 759 | 709 | 0.436 |
| 657 | 886 | 0.465 |
| 687 | 1020 | 0.476 |
| 686 | 1025 | 0.476 |
| 670 | 1029 | 0.479 |
| 555 | 1036 | 0.479 |
| 669 | 1083 | 0.482 |
| 626 | 1753 | 0.585 |
| 500B | 2149 | 0.612 |
| 602 | 2165 | 0.612 |

Supplementary Table S4. Predicted probability of LTF for individual wolves (by wolf ID) with missing endpoint (n=26) following IM2 (Supplementary Table S3) used for simulating endpoint. *P(ltf)* refers to the probability of LTF during the winter and during the period January 27, 2012 (winter=1), when wolves were delisted (lib_kill=1) and April 14, 2012, when our study period ended.

| **Model (M#)** | **ll(model)** | **df** | **AIC** | **BIC** | **Model description** |
| --- | --- | --- | --- | --- | --- |
|  |  |  |  |  |  |
| Model 1 | -2519.857 | 24 | 5087.714 | 5269.762 | endpoint-specific interactions with all covariate factor levels |
|  |  |  |  |  |  |
| Model 2 | -2527.014 | 14 | 5082.028 | 5188.223 | endpoint-specific interactions for lib_kill & winter; method_change general effect |
|  |  |  |  |  |  |
| Model 3 | -2512.954 | 16 | 5057.908 | 5179.273 | Model 2 + all tvcs |
|  |  |  |  |  |  |
| Model 4 | -2510.178 | 15 | 5050.356 | 5164.136 | endpoint-specific interactions for lib_kill & winter, method_change(2)*poa and tvcs |
|  |  |  |  |  |  |
| Model 5 | -2510.884 | 13 | 5047.769 | 5146.378 | only significant variables + tvcs (‘*BEST’ model*) |
|  |  |  |  |  |  |
| Model 6 | -2513.832 | 12 | 5051.665 | 5142.689 | Model 5 without lib_kill*legal tvc (*2nd best*) |
|  |  |  |  |  |  |
| Model 7 | -2524.869 | 11 | 5071.737 | 5155.176 | Model 5 without tvcs (*3rd best*) |

Supplementary Table S5. Model selection statistics for stratified joint Cox models for analyses of hazards using Lunn & McNeil's ^44^ data augmentation Method B. We present log-likelihood (ll), degrees of freedom (df), AIC and BIC statistics, and a model description, for all models. Model statistics suggest Model 5 (M5) was the best model. M7 (M5 without tvcs) was used for Cox CIF predictions.

| **Parameter (covariate*endpoint)** | **rho** | **chi2** | **df** | **Prob>χ^2^** |
| --- | --- | --- | --- | --- |
| lib_kill*collision | 0.031 | 0.41 | 1 | 0.521 |
| lib_kill*legal | 0.101 | 4.40 | 1 | 0.036 |
| lib_kill*ltf | -0.071 | 2.64 | 1 | 0.104 |
| ib_kill*nonhuman | 0.028 | 0.42 | 1 | 0.517 |
| lib_kill*poached | 0.056 | 1.35 | 1 | 0.246 |
| lib_kill*uncertain | 0.021 | 0.20 | 1 | 0.656 |
| winter*collision | 0.006 | 0.01 | 1 | 0.915 |
| winter*ltf | -0.248 | 43.67 | 1 | 0.000 |
| winter*nonhuman | 0.004 | 0.01 | 1 | 0.917 |
| winter*poached | -0.043 | 1.24 | 1 | 0.265 |
| 2.monit_period*poached | -0.012 | 0.07 | 1 | 0.789 |

Supplementary Table S6. Test of proportional hazards assumptions (Chi-squared, χ^2^) for parameters in the stratified joint Cox Lunn & McNeil ^44^ model M7 (Supplementary Table S5), used for evaluating proportionality assumptions. The tests suggest non-proportionality present in the interactions *lib_kill*legal* and *winter*ltf*. The tvcs in M5, the best stratified joint Cox hazards model, appropriately model the observed non-proportionality through interactions of these endpoint-covariate combinations with analysis time (i.e., tvc) (M5, Supplementary Table S5).

| **Variable** | **LTF HR** | | **LTF SHR** | |
| --- | --- | --- | --- | --- |
|  | MAIN | LOW-HIGH | MAIN | LOW-HIGH |
| *Periods liberalizing wolf-killing (lib_kill)* | 1.18 | 1.10-1.33 | 1.19 | 1.11-1.34 |
| *Season (winter)* | 3.13 | 3.21-2.55 | 2.89 | 2.92-2.4 |
| *Census (method_change)* |  |  |  |  |
| *Census method 1* | 1 | 1 | 1 | 1 |
| *Census method 2* | 1 | 1 | 1 | 1 |
| *Census method 3* | 1 | 1 | 1 | 1 |
| *time-varying coefficient (tvc)* |  |  |  |  |
| *Periods liberalizing wolf-killing (lib_kill tvc) (change per year)* | 1 | 1 | 1 | 1 |
| *Season (winter tvc) (change per year)* | 0.69 | 0.48-0.69 | 0.69 | 0.69-0.69 |

Supplementary Table S7. Lost-to-follow up (LTF) hazard (HR) and subhazard ratio (SHR) point estimates for collared wolves for all imputation scenarios (MAIN, LOW and HIGH). M5 was the best final stratified joint Cox (HR) model for all scenarios.

|  | **Cox (hazard) model** | | | | **Fine-Gray (subhazard) model** | | | |
| --- | --- | --- | --- | --- | --- | --- | --- | --- |
| **Variable** | **Legal** | | **LTF** | | **Legal** | | **LTF** | |
|  | HR | 95% CI | HR | 95% CI | SHR | 95% CI | SHR | 95% CI |
| *Periods liberalizing wolf-killing (lib_kill)* | 3.3 | 1.67-6.52 | 1.2 | 0.89-1.62 | 3.7 | 1.83-7.49 | 1.24 | 0.9-1.71 |
| *Season (winter)* | 1 | - | 1.69 | 1.19-2.40 | 1 | - | 1.32 | 0.94-1.83 |
| *Census (method_change)* |  |  |  |  |  |  |  |  |
| *Census method 1* | 1 | - | 1 | - | 1 | - | 1 | - |
| *Census method 2* | 1 | - | 1 | - | 1 | - | 1 | - |
| *Census method 3* | 1 | - | 1 | - | 1 | - | 1 | - |

Supplementary Table S8. Cox (hazard ratio, HR) and Fine-Gray (subhazard, SHR) point estimates and compatibility intervals (95% CI) of models without tvc used for estimation of cumulative incidence functions (CIFs) for legal and LTF endpoints for MAIN imputation scenario, given computational limitations of predicting hazards with tvc.

| **Variable** | **LTF HR** | | **LTF SHR** | |
| --- | --- | --- | --- | --- |
|  | MAIN | LOW-HIGH | MAIN | LOW-HIGH |
| *Periods liberalizing wolf-killing (lib_kill)* | 1.2 | 1.12-1.35 | 1.24 | 1.15-1.38 |
| *Season (winter)* | 1.69 | 1.80-1.41 | 1.32 | 1.41-1.17 |
| *Census (method_change)* |  |  |  |  |
| *Census method 1* | 1 | 1 | 1 | 1 |
| *Census method 2* | 1 | 1 | 1 | 1 |
| *Census method 3* | 1 | 1 | 1 | 1 |

Supplementary Table S9. Lost-to-follow up (LTF) Cox (hazard, HR) and Fine-Gray (subhazard, SHR) point estimates for collared wolves used for estimating Cox and Fine-Gray cumulative incidence functions (CIFs), respectively, for all imputation scenarios (MAIN, LOW and HIGH).

Supplementary Data S1 to S3

Supplementary Data S1. (separate .xlsx file)

Original dataset containing monitoring histories for n=513 collared wolves (1979-2012).

Supplementary Data S2. (separate .xlsx file)

Subsample (n=40) of collared wolves in Data S1 with missing endpoint records before the end of our analysis period (April 14, 2012), along with information on subsequent endpoint category and date if found in subsequent agency reports (n=14). We were unable to find data for n=26 wolves.

Supplementary Data S3. (separate .xlsx file)

Results of imputation of LTF or censoring (n=26 wolves) for three LTF probability scenarios (MAIN, LOW, HIGH).

Statistical code (STATA)

Statistical code for all analyses conducted in STATA.

**WI wolf data Survival Analysis – Competing risk (for data expansion, setup and simulation runs)**

clear all

******************************************************************************

***DATA EXPANSION AND SETUP**************************************************

*Following Lunn & McNeill's (1995) data expansion approach (to use Cox model)*

use "Data S1.dta", replace

tab cause_endpoint

*Following LTF Simulation data (FG) for MAIN scenario - replace with wolf IDs from simulation)**

replace cause_endpoint="censored" if cause_endpoint2==4 & wolf_ID=="788" | cause_endpoint2==4 & wolf_ID=="766/765" | ///

cause_endpoint2==4 & wolf_ID=="825" | cause_endpoint2==4 & wolf_ID=="756" | cause_endpoint2==4 & wolf_ID=="789" | ///

cause_endpoint2==4 & wolf_ID=="807" | cause_endpoint2==4 & wolf_ID=="665" | cause_endpoint2==4 & wolf_ID=="690" | ///

cause_endpoint2==4 & wolf_ID=="791" | cause_endpoint2==4 & wolf_ID=="776" | cause_endpoint2==4 & wolf_ID=="760" | ///

cause_endpoint2==4 & wolf_ID=="657" | cause_endpoint2==4 & wolf_ID=="687" | cause_endpoint2==4 & wolf_ID=="686" | ///

cause_endpoint2==4 & wolf_ID=="670" | cause_endpoint2==4 & wolf_ID=="555" | cause_endpoint2==4 & wolf_ID=="669" | ///

cause_endpoint2==4 & wolf_ID=="626" | cause_endpoint2==4 & wolf_ID=="500B"

*Following LTF Simulation data (FG) for LOW scenario - replace with wolf IDs from simulation)**

replace cause_endpoint="censored" if cause_endpoint2==4 & wolf_ID=="788" | cause_endpoint2==4 & wolf_ID=="766/765" | ///

cause_endpoint2==4 & wolf_ID=="825" | cause_endpoint2==4 & wolf_ID=="756" | cause_endpoint2==4 & wolf_ID=="789" | ///

cause_endpoint2==4 & wolf_ID=="807" | cause_endpoint2==4 & wolf_ID=="665" | cause_endpoint2==4 & wolf_ID=="690" | ///

cause_endpoint2==4 & wolf_ID=="791" | cause_endpoint2==4 & wolf_ID=="776" | cause_endpoint2==4 & wolf_ID=="760" | ///

cause_endpoint2==4 & wolf_ID=="657" | cause_endpoint2==4 & wolf_ID=="687" | cause_endpoint2==4 & wolf_ID=="686" | ///

cause_endpoint2==4 & wolf_ID=="670" | cause_endpoint2==4 & wolf_ID=="555" | cause_endpoint2==4 & wolf_ID=="669" | ///

cause_endpoint2==4 & wolf_ID=="626" | cause_endpoint2==4 & wolf_ID=="500B"

*Following LTF Simulation data (FG) for HIGH scenario - replace with wolf IDs from simulation)**

replace cause_endpoint="censored" if cause_endpoint2==4 & wolf_ID=="769" | cause_endpoint2==4 & wolf_ID=="755" | ///

cause_endpoint2==4 & wolf_ID=="759" | cause_endpoint2==4 & wolf_ID=="686" | cause_endpoint2==4 & wolf_ID=="555" | ///

cause_endpoint2==4 & wolf_ID=="602"

drop cause_endpoint2

encode cause_endpoint, gen(cause_endpoint_enc)

order cause_endpoint_enc, after(cause_endpoint)

expand 6

by wolf_ID, sort: gen cause_endpoint2= _n+1

order cause_endpoint2, after(cause_endpoint_enc)

/*Generating cause of endpoint binary variables*/

gen collision = cause_endpoint2==2

gen legal = cause_endpoint2==3

gen ltf = cause_endpoint2==4

gen nonhuman = cause_endpoint2==5

gen poached = cause_endpoint2==6

gen unknown = cause_endpoint2==7

gen event = (cause_endpoint2==cause_endpoint_enc)

gen censored = cause_endpoint_enc==1

*Generate ID var for each expanded record (wolf_ID - [1-6]) (CLUSTER FOR THIS INSTEAD OF wolf_ID)

gen wolf_ID_exp=wolf_ID+"-"+string(cause_endpoint2, "%02.0f")

order wolf_ID_exp, after(wolf_ID)

*Checking stset

stset date_endpoint, failure(event) exit(failure) origin(time capture_date) id(wolf_ID_exp)

*****TIME-SPLITTING for time-dependent variables and 'spells'*****

*****TREATMENT VARIABLE*****

stsplit treat_split, at(15795 16466 16526 16692 16914 17013 17236 17803 18020 18078 19018 19097) ///

after(capture_date==1/1/1960)

***Generating liberalized killing treatment binary variable (1 if lib kill period)

gen lib_kill = 0

replace lib_kill = 1 if treat_split==15795 | treat_split==16526 | treat_split==16914 | treat_split==17236 ///

| treat_split==18020 | treat_split==19018

tab lib_kill if _d==1

*****WINTER-SUMMER VARIABLE*****

stsplit sum_wint_split, at(7029 7212 7395 7578 7760 7943 8125 8308 8490 8673 8856 9039 ///

9221 9404 9586 9769 9951 10134 10317 10500 10682 10865 11047 11230 11412 11595 11778 11961 ///

12143 12326 12508 12691 12873 13056 13239 13422 13604 13787 13969 14152 14334 14517 14700 ///

14883 15065 15248 15430 15613 15795 15978 16161 16344 16526 16709 16891 17074 17256 17439 ///

17622 17805 17987 18170 18352 18535 18717 18900 19083 19266) after(capture_date==1/1/1960)

**Generating winter (1) time-dep binary variable

gen winter = 0

replace winter = 1 if sum_wint_split==7212 | sum_wint_split==7578 | sum_wint_split==7943 | ///

sum_wint_split==8308 | sum_wint_split==8673 | sum_wint_split==9039 | sum_wint_split==9404 | ///

sum_wint_split==9769 | sum_wint_split==10134 | sum_wint_split==10500 | sum_wint_split==10865 | ///

sum_wint_split==11230 | sum_wint_split==11595 | sum_wint_split==11961 | sum_wint_split==12326 | ///

sum_wint_split==12691 | sum_wint_split==13056 | sum_wint_split==13422 | sum_wint_split==13787 | ///

sum_wint_split==14152 | sum_wint_split==14517 | sum_wint_split==14883 | sum_wint_split==15248 | ///

sum_wint_split==15613 | sum_wint_split==15978 | sum_wint_split==16344 | sum_wint_split==16709 | ///

sum_wint_split==17074 | sum_wint_split==17439 | sum_wint_split==17805 | sum_wint_split==18170 | ///

sum_wint_split==18535 | sum_wint_split==18900 | sum_wint_split==19266

tab lib_kill winter

tab lib_kill winter if _d==1

*Generating time period variable (for controlling for time series & changes in monitoring methods)

gen method_change=1 if year(date_endpoint)<1995

replace method_change=2 if year(date_endpoint)>=1995 & year(date_endpoint)<=2000

replace method_change=3 if year(date_endpoint)>2000

tab year_endpoint method_change if _d==1

tab method_change lib_kill if _d==1

tab method_change lib_kill if _d==1 & year_endpoint>=2003

tab cause_endpoint_enc lib_kill if _d==1 & method_change==3

tab cause_endpoint_enc lib_kill if _d==1 & year_endpoint>=2002

replace event=0 if event==.

*Replace all 28 0s by adding .125 to date_endpoint... or use days_to_endpoint

replace date_endpoint = date_endpoint+.125 if date_endpoint==capture_date

***SAVE DATASET***

save "Data S1 expanded.dta", replace

tab cause_endpoint_enc if _d==1

******************************************************************************

**DESCRIPTIVES USING LAST SPELLS OF EACH SUBJECT*********************************

******************************************************************************

use "Data S1 expanded.dta", replace

******************************************************************************

***JOINT ST COX MODELS FOR ALL CAUSE-SPECIFIC HAZARDS***************************

******************************************************************************

*Checking stset by wolf_ID_exp (for multiple records)

stset date_endpoint, failure(event) exit(failure) origin(time capture_date) id(wolf_ID_exp)

stdes

stsum

******************************************************************************

**Models for CAUSE-SPECIFIC HAZARDS --> same results as stcox for specific causes (seen above); sample code for NONH cause of failure

**STRATIFIED COX MODEL

stcox i.lib_kill i.winter i.method_change, efron strata(cause_endpoint2) robust cluster(wolf_ID)

*failure-specific model (sample code)

stcox i.lib_kill i.winter if ltf==1 , tvc(i.winter) efron robust cluster(wolf_ID) //or if cause_endpoint2==2//

estat ic

stphplot if ltf==1, by(lib_kill) nolnt

******************************************************************************

***CAUSE-SPECIFIC HAZARD RATES FOR ALL CAUSES SIMULTANEOUSLY***

*Fit all in same model (basically same results as with separate PH models for all above)

stcox 1.lib_kill#2.cause_endpoint2 1.lib_kill#3.cause_endpoint2 ///

1.lib_kill#4.cause_endpoint2 1.lib_kill#5.cause_endpoint2 1.lib_kill#6.cause_endpoint2 ///

1.lib_kill#7.cause_endpoint2 1.winter#2.cause_endpoint2 1.winter#3.cause_endpoint2 ///

1.winter#4.cause_endpoint2 1.winter#5.cause_endpoint2 1.winter#6.cause_endpoint2 ///

1.winter#7.cause_endpoint2 2.method_change#2.cause_endpoint2 2.method_change#3.cause_endpoint2 ///

2.method_change#4.cause_endpoint2 2.method_change#5.cause_endpoint2 2.method_change#6.cause_endpoint2 ///

2.method_change#7.cause_endpoint2 3.method_change#2.cause_endpoint2 3.method_change#3.cause_endpoint2 ///

3.method_change#4.cause_endpoint2 3.method_change#5.cause_endpoint2 3.method_change#6.cause_endpoint2 ///

3.method_change#7.cause_endpoint2, efron strata(cause_endpoint2) robust cluster(wolf_ID)

estat ic

*monit period not failure-specific

stcox 1.lib_kill#2.cause_endpoint2 1.lib_kill#3.cause_endpoint2 ///

1.lib_kill#4.cause_endpoint2 1.lib_kill#5.cause_endpoint2 1.lib_kill#6.cause_endpoint2 ///

1.lib_kill#7.cause_endpoint2 1.winter#2.cause_endpoint2 1.winter#3.cause_endpoint2 ///

1.winter#4.cause_endpoint2 1.winter#5.cause_endpoint2 1.winter#6.cause_endpoint2 ///

1.winter#7.cause_endpoint2 i.method_change, efron strata(cause_endpoint2) robust cluster(wolf_ID)

estat ic

test 2.method_change 3.method_change

/*Sample code for checking assumptions*/

estat phtest, log detail

estat phtest, plot(1.lib_kill#3.cause_endpoint2)

estat phtest, plot(1.winter#4.cause_endpoint2)

stphplot if cause_endpoint2==3, by(lib_kill) nolnt

stphplot if cause_endpoint2==4, by(winter) nolnt

*adding tvc, general method_change and suspect tvcs

stcox 1.lib_kill#2.cause_endpoint2 1.lib_kill#3.cause_endpoint2 ///

1.lib_kill#4.cause_endpoint2 1.lib_kill#5.cause_endpoint2 1.lib_kill#6.cause_endpoint2 ///

1.lib_kill#7.cause_endpoint2 1.winter#2.cause_endpoint2 1.winter#3.cause_endpoint2 ///

1.winter#4.cause_endpoint2 1.winter#5.cause_endpoint2 1.winter#6.cause_endpoint2 ///

1.winter#7.cause_endpoint2 i.method_change, tvc(1.lib_kill#3.cause_endpoint2 1.winter#4.cause_endpoint2) efron strata(cause_endpoint2) robust cluster(wolf_ID)

estat ic

****WITH TVC and MONIT PERIOD for POA***

stcox 1.lib_kill#2.cause_endpoint2 1.lib_kill#3.cause_endpoint2 ///

1.lib_kill#4.cause_endpoint2 1.lib_kill#5.cause_endpoint2 1.lib_kill#6.cause_endpoint2 ///

1.lib_kill#7.cause_endpoint2 1.winter#2.cause_endpoint2 1.winter#3.cause_endpoint2 ///

1.winter#4.cause_endpoint2 1.winter#5.cause_endpoint2 1.winter#6.cause_endpoint2 ///

1.winter#7.cause_endpoint2 2.method_change#6.cause_endpoint2, tvc(1.lib_kill#3.cause_endpoint2 1.winter#4.cause_endpoint2) efron strata(cause_endpoint2) robust cluster(wolf_ID)

estat ic

*without LIB_KILL TVC for LEG

stcox 1.lib_kill#2.cause_endpoint2 1.lib_kill#3.cause_endpoint2 ///

1.lib_kill#4.cause_endpoint2 1.lib_kill#5.cause_endpoint2 1.lib_kill#6.cause_endpoint2 ///

1.lib_kill#7.cause_endpoint2 1.winter#2.cause_endpoint2 1.winter#3.cause_endpoint2 ///

1.winter#4.cause_endpoint2 1.winter#5.cause_endpoint2 1.winter#6.cause_endpoint2 ///

1.winter#7.cause_endpoint2 2.method_change#6.cause_endpoint2, tvc(1.winter#4.cause_endpoint2) efron strata(cause_endpoint2) robust cluster(wolf_ID)

estat ic

****WITH SIG VARIABLES FROM CAUSE-SPECIFIC COX MODELS ABOVE and winter/ltf TVC****

stcox 1.lib_kill#2.cause_endpoint2 1.lib_kill#3.cause_endpoint2 ///

1.lib_kill#4.cause_endpoint2 1.lib_kill#5.cause_endpoint2 1.lib_kill#6.cause_endpoint2 ///

1.lib_kill#7.cause_endpoint2 1.winter#2.cause_endpoint2 1.winter#4.cause_endpoint2 ///

1.winter#5.cause_endpoint2 1.winter#6.cause_endpoint2 2.method_change#6.cause_endpoint2, tvc(1.lib_kill#3.cause_endpoint2 1.winter#4.cause_endpoint2) efron strata(cause_endpoint2) robust cluster(wolf_ID)

estat ic

stcox 1.lib_kill#2.cause_endpoint2 1.lib_kill#3.cause_endpoint2 ///

1.lib_kill#4.cause_endpoint2 1.lib_kill#5.cause_endpoint2 1.lib_kill#6.cause_endpoint2 ///

1.lib_kill#7.cause_endpoint2 1.winter#2.cause_endpoint2 1.winter#4.cause_endpoint2 ///

1.winter#5.cause_endpoint2 1.winter#6.cause_endpoint2 2.method_change#6.cause_endpoint2, tvc(1.winter#4.cause_endpoint2) efron strata(cause_endpoint2) robust cluster(wolf_ID)

estat ic

/*using model above without tvc for tests, diagnostics and curves*/

stcox 1.lib_kill#2.cause_endpoint2 1.lib_kill#3.cause_endpoint2 ///

1.lib_kill#4.cause_endpoint2 1.lib_kill#5.cause_endpoint2 1.lib_kill#6.cause_endpoint2 ///

1.lib_kill#7.cause_endpoint2 1.winter#2.cause_endpoint2 1.winter#4.cause_endpoint2 ///

1.winter#5.cause_endpoint2 1.winter#6.cause_endpoint2 2.method_change#6.cause_endpoint2, efron strata(cause_endpoint2) robust cluster(wolf_ID)

estat ic

estat phtest, log detail

estat phtest, plot(1.lib_kill#2.cause_endpoint2)

estat phtest, plot(1.lib_kill#3.cause_endpoint2)

estat phtest, plot(1.lib_kill#4.cause_endpoint2)

estat phtest, plot(1.lib_kill#5.cause_endpoint2)

estat phtest, plot(1.lib_kill#6.cause_endpoint2)

estat phtest, plot(1.lib_kill#7.cause_endpoint2)

estat phtest, plot(1.winter#2.cause_endpoint2)

estat phtest, plot(1.winter#4.cause_endpoint2)

estat phtest, plot(1.winter#5.cause_endpoint2)

estat phtest, plot(1.winter#6.cause_endpoint2)

estat phtest, plot(2.method_change#6.cause_endpoint2)

stphplot if cause_endpoint2==3, by(lib_kill) nolnt

stphplot if cause_endpoint2==4, by(lib_kill) nolnt

stphplot if cause_endpoint2==4, by(winter) nolnt

stphplot if lib_kill==1, by(cause_endpoint2) nolnt

stphplot if lib_kill==0, by(cause_endpoint2) nolnt

******************************************************************************

*****TIME-SPLITTING DATASET FOR COMPETING RISK ANALYSES*************************

******************************************************************************

use "Data S1.dta", replace

set more off, permanently

******************************************************************************

******************************************************************************

stset date_endpoint, failure(cause_endpoint2==2 3 4 5 6 7) exit(failure) origin(time capture_date) id(wolf_ID)

list wolf_ID _t0 _t _d _st in 1/20

stdes

stsum

*****TIME-SPLITTING for time-dependent variables and 'spells'*****

*****TREATMENT VARIABLE*****

stsplit treat_split, at(15795 16466 16526 16692 16914 17013 17236 17803 18020 18078 19018 19097) ///

after(capture_date==1/1/1960)

***Generating liberalized killing treatment binary variable (1 if lib kill period)

gen lib_kill = 0

replace lib_kill = 1 if treat_split==15795 | treat_split==16526 | treat_split==16914 | treat_split==17236 ///

| treat_split==18020 | treat_split==19018

tab lib_kill if _d==1

tab cause_endpoint2 lib_kill if _d==1

*****WINTER-SUMMER VARIABLE*****

stsplit sum_wint_split, at(7029 7212 7395 7578 7760 7943 8125 8308 8490 8673 8856 9039 ///

9221 9404 9586 9769 9951 10134 10317 10500 10682 10865 11047 11230 11412 11595 11778 11961 ///

12143 12326 12508 12691 12873 13056 13239 13422 13604 13787 13969 14152 14334 14517 14700 ///

14883 15065 15248 15430 15613 15795 15978 16161 16344 16526 16709 16891 17074 17256 17439 ///

17622 17805 17987 18170 18352 18535 18717 18900 19083 19266) after(capture_date==1/1/1960)

**Generating winter (1) time-dep binary variable

gen winter = 0

replace winter = 1 if sum_wint_split==7212 | sum_wint_split==7578 | sum_wint_split==7943 | ///

sum_wint_split==8308 | sum_wint_split==8673 | sum_wint_split==9039 | sum_wint_split==9404 | ///

sum_wint_split==9769 | sum_wint_split==10134 | sum_wint_split==10500 | sum_wint_split==10865 | ///

sum_wint_split==11230 | sum_wint_split==11595 | sum_wint_split==11961 | sum_wint_split==12326 | ///

sum_wint_split==12691 | sum_wint_split==13056 | sum_wint_split==13422 | sum_wint_split==13787 | ///

sum_wint_split==14152 | sum_wint_split==14517 | sum_wint_split==14883 | sum_wint_split==15248 | ///

sum_wint_split==15613 | sum_wint_split==15978 | sum_wint_split==16344 | sum_wint_split==16709 | ///

sum_wint_split==17074 | sum_wint_split==17439 | sum_wint_split==17805 | sum_wint_split==18170 | ///

sum_wint_split==18535 | sum_wint_split==18900 | sum_wint_split==19266

tab lib_kill winter

tab lib_kill winter if _d==1

tab lib_kill winter if year_endpoint>=2003 & _d==1

table cause_endpoint2 lib_kill winter if _d==1, contents(freq)

table cause_endpoint2 lib_kill winter if year_endpoint>=2003 & _d==1, contents(freq)

*Generating time period variable (for controlling for time series & changes in monitoring methods)

gen method_change=1 if year(date_endpoint)<1995

replace method_change=2 if year(date_endpoint)>=1995 & year(date_endpoint)<=2000

replace method_change=3 if year(date_endpoint)>2000

tab year_endpoint method_change if _d==1

tab method_change lib_kill if _d==1

tab method_change lib_kill if _d==1 & year_endpoint>=2003

tab cause_endpoint2 method_change if _d==1

tab cause_endpoint2 if year_endpoint>=2003 & _d==1

tab cause_endpoint2 if year_endpoint<2003 & _d==1

save "Data S1 split.dta", replace

******************************************************************************

***CHECKING FOR CAUSE-SPECIFIC DIFFERENCES IN HAZARD & PH ASSUMPTION************

******************************************************************************

**FOR THE DATASETS USED IN THIS .DO FILE**

use "Data S1 split.dta", replace

*Following LTF Simulation data (FG) for MAIN scenario - replace with wolf IDs from simulation)**

replace cause_endpoint2=1 if cause_endpoint2==4 & wolf_ID=="788" | cause_endpoint2==4 & wolf_ID=="766/765" | ///

cause_endpoint2==4 & wolf_ID=="825" | cause_endpoint2==4 & wolf_ID=="756" | cause_endpoint2==4 & wolf_ID=="789" | ///

cause_endpoint2==4 & wolf_ID=="807" | cause_endpoint2==4 & wolf_ID=="665" | cause_endpoint2==4 & wolf_ID=="690" | ///

cause_endpoint2==4 & wolf_ID=="791" | cause_endpoint2==4 & wolf_ID=="776" | cause_endpoint2==4 & wolf_ID=="760" | ///

cause_endpoint2==4 & wolf_ID=="657" | cause_endpoint2==4 & wolf_ID=="687" | cause_endpoint2==4 & wolf_ID=="686" | ///

cause_endpoint2==4 & wolf_ID=="670" | cause_endpoint2==4 & wolf_ID=="555" | cause_endpoint2==4 & wolf_ID=="669" | ///

cause_endpoint2==4 & wolf_ID=="626" | cause_endpoint2==4 & wolf_ID=="500B"

*Following LTF Simulation data (FG) for LOW scenario - replace with wolf IDs from simulation)**

replace cause_endpoint2=1 if cause_endpoint2==4 & wolf_ID=="788" | cause_endpoint2==4 & wolf_ID=="766/765" | ///

cause_endpoint2==4 & wolf_ID=="825" | cause_endpoint2==4 & wolf_ID=="756" | cause_endpoint2==4 & wolf_ID=="789" | ///

cause_endpoint2==4 & wolf_ID=="807" | cause_endpoint2==4 & wolf_ID=="665" | cause_endpoint2==4 & wolf_ID=="690" | ///

cause_endpoint2==4 & wolf_ID=="791" | cause_endpoint2==4 & wolf_ID=="776" | cause_endpoint2==4 & wolf_ID=="760" | ///

cause_endpoint2==4 & wolf_ID=="657" | cause_endpoint2==4 & wolf_ID=="687" | cause_endpoint2==4 & wolf_ID=="686" | ///

cause_endpoint2==4 & wolf_ID=="670" | cause_endpoint2==4 & wolf_ID=="555" | cause_endpoint2==4 & wolf_ID=="669" | ///

cause_endpoint2==4 & wolf_ID=="626" | cause_endpoint2==4 & wolf_ID=="500B"

*Following LTF Simulation data (FG) for HIGH scenario - replace with wolf IDs from simulation)**

replace cause_endpoint2=1 if cause_endpoint2==4 & wolf_ID=="769" | cause_endpoint2==4 & wolf_ID=="755" | ///

cause_endpoint2==4 & wolf_ID=="759" | cause_endpoint2==4 & wolf_ID=="686" | cause_endpoint2==4 & wolf_ID=="555" | ///

cause_endpoint2==4 & wolf_ID=="602"

save "Data S1 split SIM.dta", replace

tab cause_endpoint2

use "Data S1 split SIM.dta", replace

**ALL EVENTS**

stset date_endpoint, failure(cause_endpoint2==2 3 4 5 6 7) exit(failure) origin(time capture_date) id(wolf_ID)

stdes

stsum

*logrank test

sts test lib_kill, strata(cause_endpoint2) d

******************************************************************************

****ST COX FOR CAUSE-SPECIFIC HAZARDS******************************************

******************************************************************************

**COLLISION**

stset date_endpoint, failure(cause_endpoint2==2) exit(failure) origin(time capture_date) id(wolf_ID)

stcox i.lib_kill, efron robust cluster(wolf_ID)

estat ic

stcox i.lib_kill, tvc(i.lib_kill) efron robust cluster(wolf_ID)

estat ic

*adding variables*/

stcox i.lib_kill i.winter i.method_change, efron robust cluster(wolf_ID)

estat ic

stcox i.lib_kill i.winter i.method_change, tvc(i.lib_kill i.winter i.method_change) efron robust cluster(wolf_ID)

estat ic

stcox lib_kill winter, efron robust cluster(wolf_ID)

estat ic

estat phtest, log detail

estat phtest, plot(lib_kill)

estat phtest, plot(winter)

stphplot, by(lib_kill) nolnt

*Goodness of fit

quietly stcox lib_kill winter, nohr efron robust cluster(wolf_ID) mgale(mg)

predict cs, csnell

stset cs, id(wolf_ID) failure(cause_endpoint2==2)

sts generate H = na

line H cs cs, sort xlab(0 1 to 4) ylab(0 1 to 4)

drop mg cs H

*predicting HR for CIFs later

stset date_endpoint, failure(cause_endpoint2==2) exit(failure) origin(time capture_date) id(wolf_ID)

stcox lib_kill winter, nohr efron robust cluster(wolf_ID)

predict h_col_0, basehc

gsort _t -_d

by _t: replace h_col_0 = . if _n > 1

gen h_col_1 = h_col_0*exp(_b[lib_kill])

twoway line h_col_* _t, connect(J J) sort lpattern(solid dash) lcolor(navy maroon) ///

legend(label(1 "Full Protections") label(2 "Reduced protections")) ///

graphregion(color(white)) title("COL Hazards STCOX")

**LEGAL**

stset date_endpoint, failure(cause_endpoint2==3) exit(failure) origin(time capture_date) id(wolf_ID)

stcox i.lib_kill, efron robust cluster(wolf_ID)

estat ic

stcox i.lib_kill, tvc(i.lib_kill) efron robust cluster(wolf_ID)

estat ic

*adding variables

stcox i.lib_kill i.winter i.method_change, tvc(i.lib_kill) efron robust cluster(wolf_ID)

estat ic

stcox i.lib_kill i.winter i.method_change, tvc(i.lib_kill i.winter i.method_change) efron robust cluster(wolf_ID)

estat ic

stcox i.lib_kill i.winter, tvc(winter) efron robust cluster(wolf_ID)

estat ic

stcox i.lib_kill i.winter, efron robust cluster(wolf_ID)

estat ic

stcox i.lib_kill, efron robust cluster(wolf_ID)

estat ic

estat phtest, log detail

estat phtest, plot(1.lib_kill)

stphplot, by(lib_kill) nolnt

*Goodness of fit

quietly stcox i.lib_kill, nohr efron robust cluster(wolf_ID) mgale(mg)

predict cs, csnell

stset cs, id(wolf_ID) failure(cause_endpoint2==3)

sts generate H = na

line H cs cs, sort xlab(0 1 to 4) ylab(0 1 to 4)

drop mg cs H

*predicting HR for CIFs later

stset date_endpoint, failure(cause_endpoint2==3) exit(failure) origin(time capture_date) id(wolf_ID)

stcox lib_kill, nohr efron robust cluster(wolf_ID)

predict h_leg_0, basehc

gsort _t -_d

by _t: replace h_leg_0 = . if _n > 1

gen h_leg_1 = h_leg_0*exp(_b[lib_kill])

twoway line h_leg_* _t, connect(J J) sort lpattern(solid dash) lcolor(navy maroon) ///

legend(label(1 "Full Protections") label(2 "Reduced protections")) ///

graphregion(color(white)) title("LEG Hazards STCOX")

**LTF**

stset date_endpoint, failure(cause_endpoint2==4) exit(failure) origin(time capture_date) id(wolf_ID)

stcox i.lib_kill, efron robust cluster(wolf_ID)

estat ic

stcox i.lib_kill, tvc(i.lib_kill) efron robust cluster(wolf_ID)

estat ic

*adding variables

stcox i.lib_kill i.winter i.method_change, efron robust cluster(wolf_ID)

estat ic

stcox i.lib_kill i.winter i.method_change, tvc(i.winter i.method_change) efron robust cluster(wolf_ID)

estat ic

stcox i.lib_kill i.winter, tvc(i.winter) efron robust cluster(wolf_ID)

estat ic

stcox lib_kill winter, efron robust cluster(wolf_ID)

estat ic

estat phtest, log detail

estat phtest, plot(winter)

stphplot, by(lib_kill) nolnt

*Goodness of fit

quietly stcox lib_kill winter, efron robust cluster(wolf_ID) mgale(mg)

predict cs, csnell

stset cs, id(wolf_ID) failure(cause_endpoint2==4)

sts generate H = na

line H cs cs, sort xlab(0 1 to 4) ylab(0 1 to 4)

drop mg cs H

*predicting HR for CIFs later

stset date_endpoint, failure(cause_endpoint2==4) exit(failure) origin(time capture_date) id(wolf_ID)

stcox lib_kill winter, nohr efron robust cluster(wolf_ID)

predict h_ltf_0, basehc

gsort _t -_d

by _t: replace h_ltf_0 = . if _n > 1

gen h_ltf_1 = h_ltf_0*exp(_b[lib_kill])

twoway line h_ltf_* _t, connect(J J) sort lpattern(solid dash) lcolor(navy maroon) ///

legend(label(1 "Full Protections") label(2 "Reduced protections")) ///

graphregion(color(white)) title("LTF Hazards STCOX")

**NONHUMAN**

stset date_endpoint, failure(cause_endpoint2==5) exit(failure) origin(time capture_date) id(wolf_ID)

stcox i.lib_kill, efron robust cluster(wolf_ID)

estat ic

stcox i.lib_kill, tvc(i.lib_kill) efron robust cluster(wolf_ID)

estat ic

*adding variables

stcox i.lib_kill i.winter i.method_change, efron robust cluster(wolf_ID)

estat ic

stcox i.lib_kill i.winter i.method_change, tvc(i.winter i.method_change) efron robust cluster(wolf_ID)

estat ic

stcox lib_kill winter, efron robust cluster(wolf_ID)

estat ic

estat phtest, log detail

estat phtest, plot(winter)

stphplot, by(lib_kill) nolnt

*Goodness of fit

quietly stcox lib_kill winter, nohr efron robust cluster(wolf_ID) mgale(mg)

predict cs, csnell

stset cs, id(wolf_ID) failure(cause_endpoint2==5)

sts generate H = na

line H cs cs, sort xlab(0 1 to 4) ylab(0 1 to 4)

drop mg cs H

*predicting HR for CIFs later

stset date_endpoint, failure(cause_endpoint2==5) exit(failure) origin(time capture_date) id(wolf_ID)

stcox lib_kill winter, nohr efron robust cluster(wolf_ID)

predict h_non_0, basehc

gsort _t -_d

by _t: replace h_non_0 = . if _n > 1

gen h_non_1 = h_non_0*exp(_b[lib_kill])

twoway line h_non_* _t, connect(J J) sort lpattern(solid dash) lcolor(navy maroon) ///

legend(label(1 "Full Protections") label(2 "Reduced protections")) ///

graphregion(color(white)) title("NONH Hazards STCOX")

**POACHED**

stset date_endpoint, failure(cause_endpoint2==6) exit(failure) origin(time capture_date) id(wolf_ID)

stcox i.lib_kill, efron robust cluster(wolf_ID)

estat ic

stcox i.lib_kill, tvc(i.lib_kill) efron robust cluster(wolf_ID)

estat ic

*adding variables

stcox i.lib_kill i.winter i.method_change, efron robust cluster(wolf_ID)

estat ic

stcox i.lib_kill i.winter i.method_change, tvc(i.winter i.method_change) efron robust cluster(wolf_ID)

estat ic

stcox lib_kill winter 2.method_change, efron robust cluster(wolf_ID)

estat ic

estat phtest, log detail

stphplot, by(lib_kill) nolnt

*Goodness of fit

quietly stcox lib_kill winter 2.method_change, nohr efron robust cluster(wolf_ID) mgale(mg)

predict cs, csnell

stset cs, id(wolf_ID) failure(cause_endpoint2==6)

sts generate H = na

line H cs cs, sort xlab(0 1 to 4) ylab(0 1 to 4)

drop mg cs H

*predicting HR for CIFs later

stset date_endpoint, failure(cause_endpoint2==6) exit(failure) origin(time capture_date) id(wolf_ID)

quietly stcox lib_kill winter 2.method_change, nohr efron robust cluster(wolf_ID)

predict h_poa_0, basehc

gsort _t -_d

by _t: replace h_poa_0 = . if _n > 1

gen h_poa_1 = h_poa_0*exp(_b[lib_kill])

twoway line h_poa_* _t, connect(J J) sort lpattern(solid dash) lcolor(navy maroon) ///

legend(label(1 "Full Protections") label(2 "Reduced protections")) ///

graphregion(color(white)) title("POA Hazards STCOX")

**UNKNOWN**

stset date_endpoint, failure(cause_endpoint2==7) exit(failure) origin(time capture_date) id(wolf_ID)

stcox i.lib_kill, efron robust cluster(wolf_ID)

estat ic

stcox i.lib_kill, tvc(i.lib_kill) efron robust cluster(wolf_ID)

estat ic

*adding variables

stcox i.lib_kill i.winter i.method_change, efron robust cluster(wolf_ID)

estat ic

stcox i.lib_kill i.winter i.method_change, tvc(i.winter i.method_change) efron robust cluster(wolf_ID)

estat ic

stcox lib_kill, efron robust cluster(wolf_ID)

estat ic

estat phtest, log detail

stphplot, by(lib_kill) nolnt

*Goodness of fit

quietly stcox lib_kill, nohr efron robust cluster(wolf_ID) mgale(mg)

predict cs, csnell

stset cs, id(wolf_ID) failure(cause_endpoint2==7)

sts generate H = na

line H cs cs, sort xlab(0 1 to 4) ylab(0 1 to 4)

drop mg cs H

*predicting HR for CIFs later

stset date_endpoint, failure(cause_endpoint2==7) exit(failure) origin(time capture_date) id(wolf_ID)

quietly stcox lib_kill, nohr efron robust cluster(wolf_ID)

predict h_unk_0, basehc

gsort _t -_d

by _t: replace h_unk_0 = . if _n > 1

gen h_unk_1 = h_unk_0*exp(_b[lib_kill])

twoway line h_unk_* _t, connect(J J) sort lpattern(solid dash) lcolor(navy maroon) ///

legend(label(1 "Full Protections") label(2 "Reduced protections")) ///

graphregion(color(white)) title("UNK Hazards STCOX")

******************************************************************************

**CREATING CIFs WITH ABOVE HAZARD CONTRIBUTIONS (see competingrisk_statuse.pdf)*

drop if missing(h_col_0) & missing(h_leg_0) & missing(h_ltf_0) & missing(h_non_0) & missing(h_poa_0) & missing(h_unk_0)

replace h_col_0=0 if missing(h_col_0)

replace h_col_1=0 if missing(h_col_1)

replace h_leg_0=0 if missing(h_leg_0)

replace h_leg_1=0 if missing(h_leg_1)

replace h_ltf_0=0 if missing(h_ltf_0)

replace h_ltf_1=0 if missing(h_ltf_1)

replace h_non_0=0 if missing(h_non_0)

replace h_non_1=0 if missing(h_non_1)

replace h_poa_0=0 if missing(h_poa_0)

replace h_poa_1=0 if missing(h_poa_1)

replace h_unk_0=0 if missing(h_unk_0)

replace h_unk_1=0 if missing(h_unk_1)

**calculating event-free survivor functions

sort _t

gen S_0 = exp(sum(log(1- h_col_0 - h_leg_0 - h_ltf_0 - h_non_0 - h_poa_0 - h_unk_0)))

gen S_1 = exp(sum(log(1- h_col_1 - h_leg_1 - h_ltf_1 - h_non_1 - h_poa_1 - h_unk_1)))

twoway line S_* _t, connect(J J) sort

*calculating CIFs

*colissions

gen cif_col_0 = sum(S_0[_n-1]*h_col_0)

gen cif_col_1 = sum(S_1[_n-1]*h_col_1)

twoway line cif_col_* _t, connect(J J) sort lpattern(solid dash) lcolor(navy maroon) ///

legend(label(1 "Full Protections") label(2 "Reduced protections")) ///

graphregion(color(white)) title("COL CIFs STCOX")

*legal

gen cif_leg_0 = sum(S_0[_n-1]*h_leg_0)

gen cif_leg_1 = sum(S_1[_n-1]*h_leg_1)

twoway line cif_leg_* _t, connect(J J) sort lpattern(solid dash) lcolor(navy maroon) ///

legend(label(1 "Full Protections") label(2 "Reduced protections")) ///

graphregion(color(white)) ytitle("Cumulative Incidence") title("LEG CIFs STCOX")

*ltf

gen cif_ltf_0 = sum(S_0[_n-1]*h_ltf_0)

gen cif_ltf_1 = sum(S_1[_n-1]*h_ltf_1)

twoway line cif_ltf_* _t, connect(J J) sort lpattern(solid dash) lcolor(navy maroon) ///

legend(label(1 "Full Protections") label(2 "Reduced protections")) ///

graphregion(color(white)) ytitle("Cumulative Incidence") title("LTF CIFs STCOX")

*nonhuman

gen cif_non_0 = sum(S_0[_n-1]*h_non_0)

gen cif_non_1 = sum(S_1[_n-1]*h_non_1)

twoway line cif_non_* _t, connect(J J) sort lpattern(solid dash) lcolor(navy maroon) ///

legend(label(1 "Full Protections") label(2 "Reduced protections")) ///

graphregion(color(white)) ytitle("Cumulative Incidence") title("NONH CIFs STCOX")

*poached

gen cif_poa_0 = sum(S_0[_n-1]*h_poa_0)

gen cif_poa_1 = sum(S_1[_n-1]*h_poa_1)

twoway line cif_poa_* _t, connect(J J) sort lpattern(solid dash) lcolor(navy maroon) ///

legend(label(1 "Full Protections") label(2 "Reduced protections")) ///

graphregion(color(white)) ytitle("Cumulative Incidence") title("POA CIFs STCOX")

*unknown

gen cif_unk_0 = sum(S_0[_n-1]*h_unk_0)

gen cif_unk_1 = sum(S_1[_n-1]*h_unk_1)

twoway line cif_unk_* _t, connect(J J) sort lpattern(solid dash) lcolor(navy maroon) ///

legend(label(1 "Full Protections") label(2 "Reduced protections")) ///

graphregion(color(white)) ytitle("Cumulative Incidence") title("UNK CIFs STCOX")

******************************************************************************

***SEMI-PARAMETRIC APPROACHES FOR CIFS****************************************

******************************************************************************

clear all

use "Data S1 split SIM.dta", replace

***FINE & GRAY APPROACH FOR COMPETING RISK, BY CAUSE OF FAILURE*****************

**COLISSION**

stset date_endpoint, failure(cause_endpoint2==2) exit(failure) origin(time capture_date) id(wolf_ID)

stcrreg i.lib_kill, compete(cause_endpoint2==3 4 5 6 7) nolog show vce(cluster wolf_ID)

estat ic

stcrreg i.lib_kill i.winter, compete(cause_endpoint2==3 4 5 6 7) nolog show vce(cluster wolf_ID)

estat ic

stcrreg i.lib_kill i.winter i.method_change, compete(cause_endpoint2==3 4 5 6 7) nolog show vce(cluster wolf_ID)

estat ic

stcrreg i.lib_kill i.winter i.method_change, tvc(i.lib_kill i.winter i.method_change) compete(cause_endpoint2==3 4 5 6 7) nolog show vce(cluster wolf_ID)

estat ic

*using best model to calculate CIFs

stcrreg i.lib_kill i.winter, compete(cause_endpoint2==3 4 5 6 7) nolog show vce(cluster wolf_ID)

stcurve, cif at1(lib_kill = 0) at2(lib_kill = 1) lpattern(solid dash) lcolor(navy maroon) ///

legend(label(1 "Full Protections") label(2 "Reduced protections")) ///

graphregion(color(white)) title("COL CIFs F&G")

predict fg_col_0, basecif

gsort _t -_d

by _t: replace fg_col_0 = . if _n > 1

gen fg_col_1 = fg_col_0*exp(_b[1.lib_kill])

**LEGAL**

stset date_endpoint, failure(cause_endpoint2==3) exit(failure) origin(time capture_date) id(wolf_ID)

stcrreg i.lib_kill, compete(cause_endpoint2==2 4 5 6 7) nolog show vce(cluster wolf_ID)

estat ic

stcrreg i.lib_kill i.winter, compete(cause_endpoint2==2 4 5 6 7) nolog show vce(cluster wolf_ID)

estat ic

stcrreg i.lib_kill i.winter i.method_change, compete(cause_endpoint2==2 4 5 6 7) nolog show vce(cluster wolf_ID)

estat ic

stcrreg i.lib_kill i.winter i.method_change, tvc(i.lib_kill i.winter i.method_change) compete(cause_endpoint2==2 4 5 6 7) nolog show vce(cluster wolf_ID)

estat ic

stcrreg i.lib_kill, tvc(lib_kill) compete(cause_endpoint2==2 4 5 6 7) nolog show vce(cluster wolf_ID)

estat ic

*using best model to calculate CIFs

stcrreg i.lib_kill, compete(cause_endpoint2==2 4 5 6 7) nolog show vce(cluster wolf_ID)

stcurve, cif at1(lib_kill = 0) at2(lib_kill = 1) lpattern(solid dash) lcolor(navy maroon) ///

legend(label(1 "Full Protections") label(2 "Reduced protections")) ///

graphregion(color(white)) title("LEG CIFs F&G")

predict fg_leg_0, basecif

gsort _t -_d

by _t: replace fg_leg_0 = . if _n > 1

gen fg_leg_1 = fg_leg_0*exp(_b[1.lib_kill])

**LTF**

stset date_endpoint, failure(cause_endpoint2==4) exit(failure) origin(time capture_date) id(wolf_ID)

stcrreg i.lib_kill, compete(cause_endpoint2==2 3 5 6 7) nolog show vce(cluster wolf_ID)

estat ic

stcrreg i.lib_kill i.winter, compete(cause_endpoint2==2 3 5 6 7) nolog show vce(cluster wolf_ID)

estat ic

stcrreg i.lib_kill i.winter i.method_change, compete(cause_endpoint2==2 3 5 6 7) nolog show vce(cluster wolf_ID)

estat ic

stcrreg i.lib_kill i.winter i.method_change, tvc(i.lib_kill i.winter i.method_change) compete(cause_endpoint2==2 3 5 6 7) nolog show vce(cluster wolf_ID)

estat ic

stcrreg i.lib_kill i.winter, tvc(i.winter) compete(cause_endpoint2==2 3 5 6 7) nolog show vce(cluster wolf_ID)

estat ic

/*ANALYSIS --> not sig 21% increase in incidence*/

*using best model to calculate CIFs (NO TVC, SO 2ND BEST)

stcrreg i.lib_kill i.winter, compete(cause_endpoint2==2 3 5 6 7) nolog show vce(cluster wolf_ID)

stcurve, cif at1(lib_kill = 0) at2(lib_kill = 1) lpattern(solid dash) lcolor(navy maroon) ///

legend(label(1 "Full Protections") label(2 "Reduced protections")) ///

graphregion(color(white)) title("LTF CIFs F&G")

predict fg_ltf_0, basecif

gsort _t -_d

by _t: replace fg_ltf_0 = . if _n > 1

gen fg_ltf_1 = fg_ltf_0*exp(_b[1.lib_kill])

twoway line fg_leg_* fg_ltf_* _t, connect(J J) sort lpattern(solid solid longdash longdash) lcolor(navy maroon navy maroon) ///

legend(label(1 "Legal w/Protections") label(2 "Legal w/Killing") label(3 "LTF w/Protections") label(4 "LTF w/Killing")) ///

graphregion(color(white)) ytitle("Cumulative Incidence") title("LEG-LTF CIFs FG")

**NONHUMAN**

stset date_endpoint, failure(cause_endpoint2==5) exit(failure) origin(time capture_date) id(wolf_ID)

stcrreg i.lib_kill, compete(cause_endpoint2==2 3 4 6 7) nolog show vce(cluster wolf_ID)

estat ic

stcrreg i.lib_kill i.winter, compete(cause_endpoint2==2 3 4 6 7) nolog show vce(cluster wolf_ID)

estat ic

stcrreg i.lib_kill i.winter i.method_change, compete(cause_endpoint2==2 3 4 6 7) nolog show vce(cluster wolf_ID)

estat ic

stcrreg i.lib_kill i.winter i.method_change, tvc(i.lib_kill i.winter i.method_change) compete(cause_endpoint2==2 3 4 6 7) nolog show vce(cluster wolf_ID)

estat ic

stcrreg i.lib_kill i.winter, tvc(i.winter) compete(cause_endpoint2==2 3 4 6 7) nolog show vce(cluster wolf_ID)

estat ic

*using best model to calculate CIFs

stcrreg i.lib_kill i.winter, compete(cause_endpoint2==2 3 4 6 7) nolog show vce(cluster wolf_ID)

stcurve, cif at1(lib_kill = 0) at2(lib_kill = 1) lpattern(solid dash) lcolor(navy maroon) ///

legend(label(1 "Full Protections") label(2 "Reduced protections")) ///

graphregion(color(white)) title("NONH CIFs F&G")

predict fg_nonh_0, basecif

gsort _t -_d

by _t: replace fg_nonh_0 = . if _n > 1

gen fg_nonh_1 = fg_nonh_0*exp(_b[1.lib_kill])

**POACHED**

stset date_endpoint, failure(cause_endpoint2==6) exit(failure) origin(time capture_date) id(wolf_ID)

stcrreg i.lib_kill, compete(cause_endpoint2==2 3 4 5 7) nolog show vce(cluster wolf_ID)

estat ic

stcrreg i.lib_kill i.winter, compete(cause_endpoint2==2 3 4 5 7) nolog show vce(cluster wolf_ID)

estat ic

/*ANALYSIS --> not sig 10% decrease in incidence*/

stcrreg i.lib_kill i.winter i.method_change, compete(cause_endpoint2==2 3 4 5 7) nolog show vce(cluster wolf_ID)

estat ic

/*ANALYSIS --> not sig 30% decrease in incidence*/

stcrreg i.lib_kill i.winter 2.method_change, compete(cause_endpoint2==2 3 4 5 7) nolog show vce(cluster wolf_ID)

estat ic

/*ANALYSIS --> not sig 24% decrease in incidence*/

*using best models to calculate CIFs*?

stcrreg i.lib_kill i.winter 2.method_change, compete(cause_endpoint2==2 3 4 5 7) nolog show vce(cluster wolf_ID)

stcurve, cif at1(lib_kill = 0) at2(lib_kill = 1) lpattern(solid dash) lcolor(navy maroon) ///

legend(label(1 "Full Protections") label(2 "Reduced protections")) ///

graphregion(color(white)) title("POA CIFs F&G")

predict fg_poa_0, basecif

gsort _t -_d

by _t: replace fg_poa_0 = . if _n > 1

gen fg_poa_1 = fg_poa_0*exp(_b[1.lib_kill])

twoway line fg_leg_* fg_ltf_* fg_poa_* _t, connect(J J) sort lpattern(solid solid longdash longdash shortdash shortdash) lcolor(navy maroon navy maroon navy maroon) ///

legend(label(1 "Legal w/Protections") label(2 "Legal w/Killing") label(3 "LTF w/Protections") label(4 "LTF w/Killing") label(5 "Poached w/Protections") label(6 "Poached w/Killing")) ///

graphregion(color(white)) ytitle("Cumulative Incidence") title("LEG,LTF,POA CIFs FG")

twoway line fg_leg_* fg_ltf_* fg_poa_* _t , connect(J J) sort lpattern(solid dash solid dash solid dash) lcolor(black black dkorange dkorange maroon maroon) ///

legend(off) graphregion(color(white)) ytitle("Cumulative Incidence") xtitle("time when monitoring ends (days)") text(.43 2500 "LTF", place(ne) color(dkorange)) text(.0 2500 "Legal", place(ne) color(black)) ///

text(.05 2500 "Poached", place(ne) color(maroon))

**UNKNOWN**

stset date_endpoint, failure(cause_endpoint2==7) exit(failure) origin(time capture_date) id(wolf_ID)

stcrreg i.lib_kill, compete(cause_endpoint2==2 3 4 5 6) nolog show vce(cluster wolf_ID)

estat ic

stcrreg i.lib_kill i.winter, compete(cause_endpoint2==2 3 4 5 6) nolog show vce(cluster wolf_ID)

estat ic

stcrreg i.lib_kill i.winter i.method_change, compete(cause_endpoint2==2 3 4 5 6) nolog show vce(cluster wolf_ID)

estat ic

stcrreg i.lib_kill i.winter i.method_change, tvc(i.lib_kill i.winter i.method_change) compete(cause_endpoint2==2 3 4 5 6) nolog show vce(cluster wolf_ID)

estat ic

*using best model to calculate CIFs --> *different model than STCOX (this one includes winter)*

stcrreg i.lib_kill i.winter, compete(cause_endpoint2==2 3 4 5 6) nolog show vce(cluster wolf_ID)

stcurve, cif at1(lib_kill = 0) at2(lib_kill = 1) lpattern(solid dash) lcolor(navy maroon) ///

legend(label(1 "Full Protections") label(2 "Reduced protections")) ///

graphregion(color(white)) title("UNK CIFs F&G")

predict fg_unk_0, basecif

gsort _t -_d

by _t: replace fg_unk_0 = . if _n > 1

gen fg_unk_1 = fg_unk_0*exp(_b[1.lib_kill])

******************************************************************************

***CHECKING F&G/STCOX MODELS AGAINST NON-PAR*********************************

******************************************************************************

***COLLISION***

stset date_endpoint, failure(cause_endpoint2==2) exit(failure) origin(time capture_date) id(wolf_ID)

stcrreg if lib_kill==0, compete(cause_endpoint2==3 4 5 6 7) nolog show vce(cluster wolf_ID)

predict nonp_cif_col_0, basecif

stcrreg if lib_kill==1, compete(cause_endpoint2==3 4 5 6 7) nolog show vce(cluster wolf_ID)

predict nonp_cif_col_1, basecif

twoway line nonp_cif_col_* _t, connect(J J) sort lpattern(solid dash) lcolor(navy maroon) ///

legend(label(1 "Full Protections") label(2 "Reduced protections")) ///

graphregion(color(white)) ytitle("Cumulative Incidence") title("COL CIFs NONPAR")

***LEGAL***

stset date_endpoint, failure(cause_endpoint2==3) exit(failure) origin(time capture_date) id(wolf_ID)

stcrreg if lib_kill==0, compete(cause_endpoint2==2 4 5 6 7) nolog show vce(cluster wolf_ID)

predict nonp_cif_leg_0, basecif

stcrreg if lib_kill==1, compete(cause_endpoint2==2 4 5 6 7) nolog show vce(cluster wolf_ID)

predict nonp_cif_leg_1, basecif

twoway line nonp_cif_leg_* _t, connect(J J) sort lpattern(solid dash) lcolor(navy maroon) ///

legend(label(1 "Full Protections") label(2 "Reduced protections")) ///

graphregion(color(white)) ytitle("Cumulative Incidence") title("LEG CIFs NONPAR")

***LTF***

stset date_endpoint, failure(cause_endpoint2==4) exit(failure) origin(time capture_date) id(wolf_ID)

stcrreg if lib_kill==0, compete(cause_endpoint2==2 3 5 6 7) nolog show vce(cluster wolf_ID)

predict nonp_cif_ltf_0, basecif

stcrreg if lib_kill==1, compete(cause_endpoint2==2 3 5 6 7) nolog show vce(cluster wolf_ID)

predict nonp_cif_ltf_1, basecif

twoway line nonp_cif_ltf_* _t, connect(J J) sort lpattern(solid dash) lcolor(navy maroon) ///

legend(label(1 "Full Protections") label(2 "Reduced protections")) ///

graphregion(color(white)) ytitle("Cumulative Incidence") title("LTF CIFs NONPAR")

***NONHUMAN***

stset date_endpoint, failure(cause_endpoint2==5) exit(failure) origin(time capture_date) id(wolf_ID)

stcrreg if lib_kill==0, compete(cause_endpoint2==2 3 4 6 7) nolog show vce(cluster wolf_ID)

predict nonp_cif_non_0, basecif

stcrreg if lib_kill==1, compete(cause_endpoint2==2 3 4 6 7) nolog show vce(cluster wolf_ID)

predict nonp_cif_non_1, basecif

twoway line nonp_cif_non_* _t, connect(J J) sort lpattern(solid dash) lcolor(navy maroon) ///

legend(label(1 "Full Protections") label(2 "Reduced protections")) ///

graphregion(color(white)) ytitle("Cumulative Incidence") title("NONH CIFs NONPAR")

***POACHED***

stset date_endpoint, failure(cause_endpoint2==6) exit(failure) origin(time capture_date) id(wolf_ID)

stcrreg if lib_kill==0, compete(cause_endpoint2==2 3 4 5 7) nolog show vce(cluster wolf_ID)

predict nonp_cif_poa_0, basecif

stcrreg if lib_kill==1, compete(cause_endpoint2==2 3 4 5 7) nolog show vce(cluster wolf_ID)

predict nonp_cif_poa_1, basecif

twoway line nonp_cif_poa_* _t, connect(J J) sort lpattern(solid dash) lcolor(navy maroon) ///

legend(label(1 "Full Protections") label(2 "Reduced protections")) ///

graphregion(color(white)) ytitle("Cumulative Incidence") title("POA CIFs NONPAR")

***UNCERTAIN***

stset date_endpoint, failure(cause_endpoint2==7) exit(failure) origin(time capture_date) id(wolf_ID)

stcrreg if lib_kill==0, compete(cause_endpoint2==2 3 4 5 6) nolog show vce(cluster wolf_ID)

predict nonp_cif_unk_0, basecif

stcrreg if lib_kill==1, compete(cause_endpoint2==2 3 4 5 6) nolog show vce(cluster wolf_ID)

predict nonp_cif_unk_1, basecif

twoway line nonp_cif_unk_* _t, connect(J J) sort lpattern(solid dash) lcolor(navy maroon) ///

legend(label(1 "Full Protections") label(2 "Reduced protections")) ///

graphregion(color(white)) ytitle("Cumulative Incidence") title("UNC CIFs NONPAR")**Predicting CIF for simulation of 4/14/2012 wolves**

******************************************************************************

**CENSORING 4/14/2014 WOLVES*************************************************

******************************************************************************

use "Data S1 split.dta", replace

tab cause_endpoint2

***LTF***

**FINE & GRAY**

stset date_endpoint, failure(cause_endpoint2==4) exit(failure) origin(time capture_date) id(wolf_ID)

stcrreg i.lib_kill, compete(cause_endpoint2==2 3 5 6 7) nolog show vce(cluster wolf_ID)

estat ic

stcrreg i.lib_kill i.winter, compete(cause_endpoint2==2 3 5 6 7) nolog show vce(cluster wolf_ID)

estat ic

stcrreg i.lib_kill i.winter i.method_change, compete(cause_endpoint2==2 3 5 6 7) nolog show vce(cluster wolf_ID)

estat ic

stcrreg i.lib_kill i.winter, tvc(i.lib_kill i.winter) compete(cause_endpoint2==2 3 5 6 7) nolog show vce(cluster wolf_ID)

estat ic

stcrreg i.lib_kill i.winter, tvc(i.winter) compete(cause_endpoint2==2 3 5 6 7) nolog show vce(cluster wolf_ID)

estat ic

stcrreg i.lib_kill winter, compete(cause_endpoint2==2 3 5 6 7) nolog show vce(cluster wolf_ID)

estat ic

predict xb

predict cif_ltf_0, basecif

gen cif_ltf_1 = cif_ltf_0*exp(_b[1.lib_kill])*exp(_b[winter])

list wolf_ID cif_ltf_1 if year_last_contact==2012 & cause_endpoint2==1

drop cif_ltf_0**LTF HR distributions**

use "LTF HR distributions.dta", replace

*POLICY

drawnorm policy_coef,n(513) means(.1627905) sds(.1557285) clear

gen policy_hr=exp(policy_coef)

sum policy_hr, d

scalar polhr_mean = r(mean)

scalar polhr_sd = r(sd)

scalar list

kdensity policy_hr

generate z_polhr = (policy_hr - polhr_mean)/polhr_sd

generate below_polhr = normal(z_polhr)

generate above_polhr = 1-normal(z_polhr)

sort policy_hr

kdensity z_polhr

sum above_polhr if policy_hr>1

twoway kdensity policy_hr, range(0 2) recast(area) color(dknavy%30) ///

xline(1, lcolor(black)) xline(1.18, lcolor(maroon) lpattern(dash)) xtitle("Hazard ratio") ytitle("Density") ///

title("Liberalized killing effect on LTF HR distribution", color(black)) ///

graphregion(color(white)) text(.5 .75 "16%", place(n)) text(.5 1.60 "84%", place(n)) text(2 1.20 "mean HR = 1.18", place(ne) color(maroon)) ///

legend(off)

graph save "Policy-LTF HR distribution", replace

graph export "Policy-LTF HR distribution.pdf", replace

*WINTER

drawnorm winter_coef,n(513) means(1.141511) sds(.2313206)

gen winter_hr=exp(winter_coef)

sum winter_hr, d

scalar winhr_mean = r(mean)

scalar winhr_sd = r(sd)

scalar list

kdensity winter_hr

generate z_winhr = (winter_hr - winhr_mean)/winhr_sd

generate below_winhr = normal(z_winhr)

generate above_winhr = 1-normal(z_winhr)

sort winter_hr

kdensity z_winhr

sum above_winhr if winter_hr>1

twoway kdensity winter_hr, range(0 5.5) recast(area) color(dknavy%30) ///

xline(1, lcolor(black)) xline(3.13, lcolor(maroon) lpattern(dash)) xtitle("Hazard ratio") ytitle("Density") ///

title("Winter season effect on LTF HR distribution", color(black)) ///

graphregion(color(white)) text(.2 1.80 "2%", place(n)) text(.2 4.5 "98%", place(n)) text(.5 3.30 "mean HR = 3.13", place(ne) color(maroon)) ///

xlabel(0(1)6) legend(off)

graph save "Winter-LTF HR distribution", replace

graph export "Winter-LTF HR distribution.pdf", replace

twoway kdensity policy_hr, range(0 5.5) recast(area) color(dknavy%30) ///

|| kdensity winter_hr, range(0 5.5) recast(area) color(dkorange%30) xline(1, lcolor(black)) xline(1.18, lcolor(dknavy) lpattern(dash)) xline(3.13, lcolor(dkorange) lpattern(dash)) xtitle("Hazard ratio") ytitle("Density") ///

title("Policy and Winter season effects on LTF HR distribution", color(black)) ///

graphregion(color(white)) text(.5 .65 "15%", place(n) color(dknavy)) text(.5 1.70 "85%", place(n) color(dknavy)) text(2 1.20 "policy mean HR = 1.18", place(ne) color(dknavy)) text(.2 .65 "1%", place(n) color(dkorange)) text(.2 4.5 "99%", place(n) color(dkorange)) text(.5 3.30 "winter mean HR = 3.13", place(ne) color(dkorange)) ///

xlabel(0(1)6) legend(off)

graph save "Policy & Winter LTF HR distribution", replace

graph export "Policy & Winter LTF HR distribution.pdf", replace

**POA HR distributions**

use "POA HR distributions.dta", replace

*POLICY

drawnorm policy_coef,n(513) means(-.2113997) sds(.262348) clear

gen policy_hr=exp(policy_coef)

sum policy_hr, d

scalar polhr_mean = r(mean)

scalar polhr_sd = r(sd)

scalar list

kdensity policy_hr

generate z_polhr = (policy_hr - polhr_mean)/polhr_sd

generate below_polhr = normal(z_polhr)

generate above_polhr = 1-normal(z_polhr)

sort policy_hr

kdensity z_polhr

sum above_polhr if policy_hr>1

twoway kdensity policy_hr, range(0 2) recast(area) color(dknavy%30) ///

xline(1, lcolor(black)) xline(0.81, lcolor(maroon) lpattern(dash)) xtitle("Hazard ratio") ytitle("Density") ///

title("Liberalized killing effect on POA HR distribution", color(black)) ///

graphregion(color(white)) text(.5 .40 "79%", place(n)) text(.5 1.25 "21%", place(n)) text(1.5 0.15 "mean HR = 0.81", place(ne) color(maroon)) ///

legend(off)

save "POA HR distributions.dta", replace

graph save "Policy-POA HR distribution", replace

graph export "Policy-POA HR distribution.pdf", replace

*WINTER

drawnorm winter_coef,n(513) means(1.546519) sds(.3271005)

gen winter_hr=exp(winter_coef)

sum winter_hr, d

scalar winhr_mean = r(mean)

scalar winhr_sd = r(sd)

scalar list

kdensity winter_hr

generate z_winhr = (winter_hr - winhr_mean)/winhr_sd

generate below_winhr = normal(z_winhr)

generate above_winhr = 1-normal(z_winhr)

sort winter_hr

kdensity z_winhr

sum above_winhr if winter_hr>1

twoway kdensity winter_hr, range(0 13) recast(area) color(dknavy%30) ///

xline(1, lcolor(black)) xline(4.70, lcolor(maroon) lpattern(dash)) xtitle("Hazard ratio") ytitle("Density") ///

title("Winter season effect on POA HR distribution", color(black)) ///

graphregion(color(white)) text(.1 .5 "3%", place(n)) text(.1 7 "97%", place(n)) text(.25 5.2 "mean HR = 4.70", place(ne) color(maroon)) ///

xlabel(0(1)13) legend(off)

graph save "Winter-POA HR distribution", replace

graph export "Winter-POA HR distribution.pdf", replace

twoway kdensity policy_hr, range(0 13) recast(area) color(dknavy%30) ///

|| kdensity winter_hr, range(0 13) recast(area) color(dkorange%30) xline(1, lcolor(black)) ///

xline(0.81, lcolor(dknavy) lpattern(dash)) xline(4.70, lcolor(dkorange) lpattern(dash)) ///

xtitle("Hazard ratio") ytitle("Density") title("Policy and Winter season effects on POA HR distribution", color(black)) ///

graphregion(color(white)) text(.5 -.1 "79%", place(ne) color(dknavy)) text(.5 1.70 "21%", place(n) color(dknavy)) ///

text(1.5 1 "policy mean HR = 0.81", place(ne) color(dknavy)) text(.1 -.1 "3%", place(ne) color(dkorange)) ///

text(.1 7 "97%", place(ne) color(dkorange)) text(.3 4.8 "winter mean HR = 4.70", place(ne) color(dkorange)) ///

xlabel(0(1)13) legend(off)

graph save "Policy & Winter POA HR distribution", replace

graph export "Policy & Winter POA HR distribution.pdf", replace

*METHOD CHANGE

drawnorm methchg_coef,n(513) means(-1.049549) sds(.3979394)

gen methchg_hr=exp(methchg_coef)

sum methchg_hr, d

scalar methchghr_mean = r(mean)

scalar methchghr_sd = r(sd)

scalar list

kdensity methchg_hr

generate z_methchghr = (methchg_hr - methchghr_mean)/methchghr_sd

generate below_methchghr = normal(z_methchghr)

generate above_methchghr = 1-normal(z_methchghr)

sort methchg_hr

kdensity z_methchghr

sum above_methchghr if methchg_hr>1

twoway kdensity methchg_hr, range(0 2) recast(area) color(dknavy%30) ///

xline(1, lcolor(black)) xline(0.35, lcolor(maroon) lpattern(dash)) xtitle("Hazard ratio") ytitle("Density") ///

title("Monitoring method (period 2) effect on POA HR distribution", color(black)) ///

graphregion(color(white)) text(1 0.05 "100%", place(n)) text(1 1.1 "0%", place(ne)) text(2 0.35 "mean HR = 0.35", place(ne) color(maroon)) ///

xlabel(0(.5)2) legend(off)

graph save "Methchg-POA HR distribution", replace

graph export "Methchg-POA HR distribution.pdf", replace

twoway kdensity policy_hr, range(0 13) recast(area) color(dknavy%30) ///

|| kdensity winter_hr, range(0 13) recast(area) color(dkorange%30) ///

|| kdensity methchg_hr, range(0 13) recast(area) color(dkgreen%30) xline(1, lcolor(black)) ///

xline(0.81, lcolor(dknavy) lpattern(dash)) xline(4.70, lcolor(dkorange) lpattern(dash)) xline(0.35, lcolor(dkgreen) lpattern(dash)) ///

xtitle("Hazard ratio") ytitle("Density") title("Covariate effects on POA HR distribution", color(black)) ///

graphregion(color(white)) text(.5 -.1 "79%", place(ne) color(dknavy) size(small)) text(.5 1.70 "21%", place(n) color(dknavy) size(small)) ///

text(1.5 1 "policy mean HR = 0.81", place(ne) color(dknavy) size(small)) text(.1 -.1 "3%", place(ne) color(dkorange) size(small)) ///

text(.1 7 "97%", place(ne) color(dkorange) size(small)) text(.3 4.8 "winter mean HR = 4.70", place(ne) color(dkorange) size(small)) ///

text(1 -.1 "100%", place(ne) color(dkgreen) size(small)) text(1 1.70 "0%", place(n) color(dkgreen) size(small)) text(2 0.35 "monit method 2 mean HR = 0.35", place(ne) color(dkgreen) size(small)) ///

xlabel(0(1)13) legend(off)

graph save "All covariates POA HR distribution", replace

graph export "All covariates POA HR distribution.pdf", replace
